# Abrogation of aberrant glycolytic interactions eliminates senescent cells and alleviates aging-related dysfunctions

**DOI:** 10.1038/s41392-025-02502-6

**Published:** 2025-12-15

**Authors:** Takumi Mikawa, Masahiro Kameda, Sumiko Ikari, Eri Shibata, Shuyu Liu, Sawa Miyagawa, Koh Ono, Tomiko Ito, Akihiko Yoshizawa, Masataka Sugimoto, Shuichi Shibuya, Takahiko Shimizu, Julio Almunia, Noboru Ogiso, Gwladys Revêchon, Alberta Palazzo, David Bernard, Hiroaki Kanda, Tomoyoshi Soga, Keiyo Takubo, Shin Morioka, Junko Sasaki, Takehiko Sasaki, Akihiro Itamoto, Takayuki Fujii, Hiroshi Seno, Nobuya Inagaki, Hiroshi Kondoh

**Affiliations:** 1 Geriatric Unit, Graduate School of Medicine, Kyoto University, Kyoto, Japan; 2 Department of Diabetes, Endocrinology and Nutrition, Graduate School of Medicine, Kyoto University, Kyoto, Japan; 3 Department of Cardiovascular Medicine, Graduate School of Medicine, Kyoto University, Kyoto, Japan; 4 Department of Diagnostic Pathology, Nara Medical University, Nara, Japan; 5 Department of Cellular Pathology, National Center for Geriatrics and Gerontology, Obu, Aichi Japan; 6 Aging Stress Response Research Project Team, National Center for Geriatrics and Gerontology, Obu, Aichi Japan; 7 Department of Regenerative Medicine, Faculty of Pharmacy, Sanyo-Onoda City University, Yamaguchi, Japan; 8 Department of Food and Reproductive Function Advanced Research, Juntendo University Graduate School of Medicine, Tokyo, Japan; 9 Department of Laboratory of Experimental Animals, National Center for Geriatrics and Gerontology, Obu, Aichi Japan; 10 Faculty of Health and Medical Sciences, Aichi Shukutoku University, Obu, Aichi Japan; 11 Team Labellisée la Ligue Contre le Cancer, Cancer Research Center of Lyon, Inserm U1052, CNRS UMR 5286, Centre Léon Bérard, Lyon University, Lyon, France; 12 Department of Pathology, Saitama Cancer Center, Saitama, Japan; 13 Human Biology-Microbiome-Quantum Research Center (WPI-Bio2Q), Keio University, Tokyo, Japan; 14 Department of Cell Fate Biology and Stem Cell Medicine, Tohoku University Graduate School of Medicine, Sendai, Miyagi Japan; 15 Department of Stem Cell Biology, National Institute of Global Health and Medicine, Japan Institute for Health Security, Tokyo, Japan; 16 Department of Biochemical Pathophysiology, Medical Research Laboratory, Institute of Integrated Research, Institute of Science Tokyo, Tokyo, Japan; 17 Department of Orthopedic Surgery, Graduate School of Medicine, Kyoto University, Kyoto, Japan; 18 Department of Advanced Medicine for Rheumatic Diseases, Graduate School of Medicine, Kyoto University, Kyoto, Japan; 19 Department of Gastroenterology and Hepatology, Graduate School of Medicine, Kyoto University, Kyoto, Japan

**Keywords:** Senescence, Translational research

## Abstract

Cellular senescence is deeply involved in physiological homeostasis, development, tissue repair, aging, and diseases. Senescent cells (SnCs) accumulate in aged tissues and exert deleterious effects by secreting proinflammatory molecules that contribute to chronic inflammation and aging-related diseases. We revealed that an aberrant interaction between glycolytic PGAM1 and Chk1 kinase is augmented in SnCs associated with increased glycolysis, whose byproduct, lactate, promotes this binding in a noncell autonomous manner. The pseudo-Warburg effect of SnCs with enhanced PPP (pentose phosphate pathway) activity is maintained by HIF-2α phosphorylation by Chk1 and subsequent upregulation of glycolytic enzymes, creating a vicious cycle reprogramming the glycolytic pathway in SnCs. HIF-2α also activates FoxM1 expression, which transcriptionally suppresses proapoptotic profiles, including BIM, and upregulates DNA repair machineries in SnCs. FoxM1 thus supports the genomic integrity and survival capacity of SnCs during their glycolytic changes. Chemical abrogation of PGAM1-Chk1 binding reverts these phenotypes and eliminates SnCs through senolysis. Inhibition of the PGAM1-Chk1 interaction improves physiological parameters during aging and inhibits lung fibrosis in mouse models. Our study highlights a novel pathway contributing to the metabolic reprogramming of SnCs and how the use of a new senolytic molecule that targets the PGAM-Chk1 interaction creates a specific vulnerability of those cells to potentially fight age-related diseases.

## Introduction

During their life course, normal tissues are frequently exposed to diverse stresses, the damaging effects of which cannot be neglected. The ability of resilience to maintain physiological homeostasis comprises adaptative reactions, repair, apoptosis, or defensive responses, which allow tissues and cells to adapt to various stimuli and recover from damage or injury. A sufficient capacity for resilience restores cellular and tissue function, while gradual declines or age-related alterations in resilience are concomitant with or followed by tissue and organismal aging.^1^ Senescent cells (SnCs) suffer irreversible cell cycle arrest with functional decline or changes caused by senescence-inducing stresses (DNA damage, oncogenic insults, e.g., activation of oncogenic Ras signaling, telomeric erosion, etc.).^2^ SnCs are implicated both in health and disease. These cells can exert beneficial functions in vivo, comprising a protective barrier against tumorigenesis, and serve physiological functions, including tissue repair^3^ and promoting tissue remodeling during development.^4^ However, the accumulation of SnCs can elicit deleterious effects via the secretion of proinflammatory factors,^5^ as inflammatory signals are hyperactivated due to DNA damage, oxidative stress, or other hallmarks of senescence.^6^ This persistent secretome, named the senescence-associated secretory phenotype (SASP), induces chronic sterile inflammation in tissues. Thus, chronic inflammation provoked by the accumulation of SnCs impairs resilience ability and tissue functions in aged organisms, which in turn can expedite the progression of aging-related diseases.^7^

Metabolic flexibility is one of the resilience properties and is well known to occur under several stresses. For example, to meet energy demand, physical exercise increases lipid oxidation and glucose uptake,^8^ whereas long-term fasting provokes diverse catabolic reactions after glycogen storage is depleted.^9^ Thus, glycolysis, a catalytic reaction of glucose to generate energy sources and macromolecules, maintains the physiological energy supply and is strongly modulated as adaptive responses. For example, the hypoxic response involves a metabolic shift toward enhanced glycolysis, which is regulated transcriptionally and posttranscriptionally. Locally enhanced glycolysis, known as the Warburg effect, was first reported in many cancers.^10^ However, the globally concerted shift to glycolysis in vivo is less likely to occur as a physiological resilience response, as such a reaction in the whole body causes prompt depletion of glucose and severe lactic acidosis.^11^ Interestingly, aged *Drosophila*^12^ and mice^13^ display mildly increased glycolysis in vivo. Compared with their nonsenescent counterparts, human primary SnCs also exhibit enhanced glycolysis in vitro,^14^ which partly constitutes in vivo glycolytic features in aged organisms. The increase in glycolysis in SnCs could be explained by their metabolic adaptation to meet their high energy demands or by dysregulated metabolism, including mitochondrial activity.^15,16^ However, a mechanistic understanding of its ability to maintain such a glycolytic shift during senescence and how glycolytic maladaptation is coupled with aberrant survival of SnCs is still lacking.

Senotherapy, which involves the clearance of SnCs (senolysis) or the suppression of the SASP (senomorphics), is an emerging approach to resolve aging-related disorders.^7^ Genetic, chemical, or immunological elimination of SnCs has beneficial effects on experimental models.^7,17^ Senolysis is associated with antiapoptotic activity,^18^ dysregulated proteostasis^7^ and epigenetic changes^19^ in SnCs. Dysregulated metabolism is also observed in SnCs^6^ and whether it can be targeted for senolysis, especially glycolysis, which is increased in SnCs, is largely unknown. Senescent cancer cells induced by anticancer therapy were previously reported to be vulnerable to glycolytic inhibition.^20^ However, careful design is needed to target glycolysis as a therapeutic approach, as glycolysis, a vital metabolic pathway, is also augmented in rapidly proliferating standard cells.^21^ Among glycolytic enzymes, phosphoglycerate mutase (PGAM) is a key factor that regulates glycolysis in a cellular context-dependent manner.^10,22^ Recently, we identified a nonenzymatic role of PGAM1 in cancers.^23^ In cancerous cells with oncogenic Ras signaling, but not in standard cells, PGAM1 interacts with Chk1 kinase, a checkpoint regulator of the cell cycle, and increases glycolysis.^23^

Here, we demonstrated that PGAM1-Chk1 binding is also augmented in SnCs, accompanied by increased glycolysis, whose inhibition accelerates the accumulation of DNA damage in these cells. We found that Nutlin 3b, an isomer of the well-known Nutlin 3a, efficiently interferes with PGAM1-Chk1 binding in cancers and SnCs, similar to Nutlin 3a, whereas Nutlin-3b was less effective (~150-fold) at inhibiting the p53-Mdm2 interaction than Nutlin 3a.^24^ Both Nutiln 3b and 3a induce apoptosis in SnCs. However, Nutlin 3b, not Nutlin 3a, can serve as a senolytic, as Nutlin 3b does not activate p53 in young cells. The accumulation of HIF-2α via the PGAM1-Chk1 interaction confers glycolytic features in SnCs, whose degradation is facilitated by the inhibition of PGAM1-Chk1 binding. Lactate, a byproduct of glycolytic metabolism, enhanced the PGAM1-Chk1 interaction in a noncell autonomous manner. We identified FOXM1 as a target of PGAM1-Chk1 interference via transcriptome analysis. In SnCs, activation of the FOXM1/HIF-2α axis by PGAM1-Chk1 binding modulates transcriptional profiles for the proapoptotic response, including BIM, in addition to the DNA repair machinery. As FOXM1 accumulates in aged tissues, chemical or genetic abrogation of PGAM1-Chk1 binding impedes the protective role of FOXM1 in SnCs, inducing the death of senescent cells and alleviating dysfunction, including lung fibrosis, in vivo, indicating its potential efficacy as senotherapy.

## Results

### Enhancement of the PGAM1-Chk1 interaction and glycolytic features in senescent cells (SnCs)

**Fig. 1:**
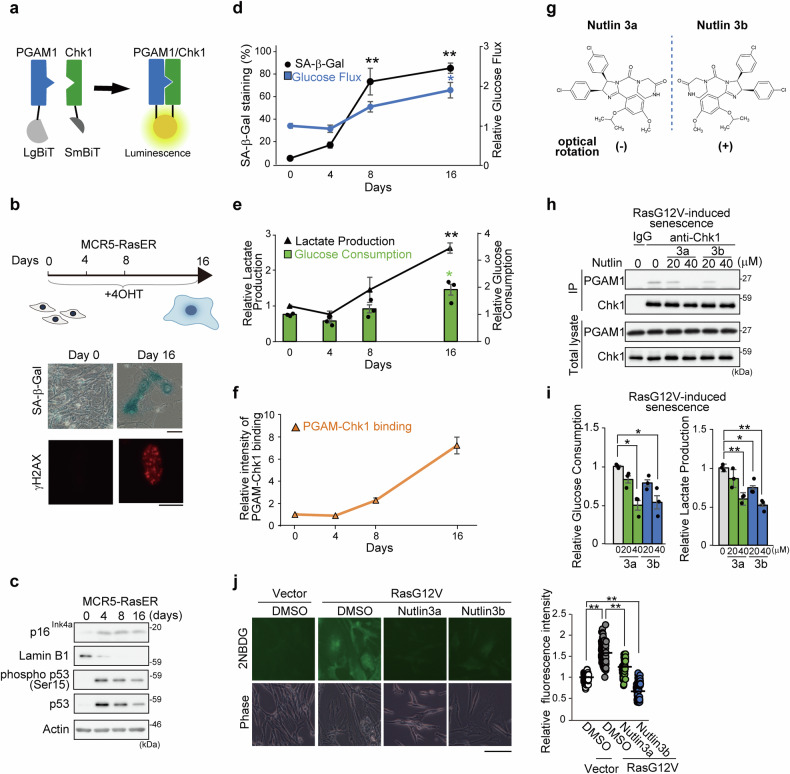
Senescent cells (SnCs) display increased interaction between PGAM1 and Chk1 and increased glycolysis. Evaluation of the PGAM1-Chk1 interaction and glycolytic activity during cellular senescence (**a**–**f**, *n* = 3, biological replicates). **a** Diagram of the NanoBiT assay used to detect PGAM1-Chk1 interactions. LgBiT-tagged PGAM1 and SmBiT-tagged Chk1 were introduced into MCR5-RasER cells. **b** MCR5-RasER cells were treated with 20 nM 4-hydroxytamoxifen (4-OHT). The cells were cultured for 16 days. SA-β GAL and γH2AX staining are shown in the upper right and lower panels, respectively. The bars indicate 100 μm and 10 μm, respectively. **c**. Senescent profiles were assessed during 4-OHT treatment. Immunoblotting for several senescence markers is shown: p16^Ink4a^, Lamin B1, p53 and phospho-p53 (Ser15) protein. **d** Evaluation of SA-β GAL staining (black) and glucose flux (blue) in MCR5-RasER cells after tamoxifen treatment. The cells were collected at the indicated time points. **e** Assessment of glycolytic activity during cellular senescence. On the indicated days, the following glycolytic parameters were assessed: lactate production (black) and glucose consumption (green bar). **f** Evaluation of the PGAM1-Chk1 interaction via the NanoBiT assay (orange) in MCR5-RasER cells after tamoxifen treatment. (**g**–**j** Effects of Nutlin 3a and 3b on the PGAM1-Chk1 interaction and glycolytic features in SnCs. Senescence was provoked in IMR90 cells by the ectopic expression of Ras G12V. **g** Diagram of the chemical structure of Nutlin isomers 3a and 3b (left and right panels, respectively). **h** Immunoprecipitation was used to detect PGAM1-Chk1 interactions in SnCs. SnCs were treated with Nutlin 3a or 3b at 20 or 40 μM for 48 h. Controls received neither compound. The data are representative of two independent experiments. **i** Glucose consumption and lactate production were measured in SnCs treated with Nutlin 3a (green) or 3b (blue) (*n* = 3 biological replicates). **j** Evaluation of 2NBDG (2-(N-(7-nitrobenz-2-oxa-1,3-diazol-4-yl)amino)-2-deoxyglucose) uptake in SnCs after Nutlin 3a (green) or 3b (blue) treatment. 2NBDG was detected as green fluorescence (left panel). The bar indicates 100 μm. Fluorescence intensities were assessed in each cell (right panel). The data represent two independent experiments. The data represent the means ± SEMs of three biological replicates. Single (*) and double (**) asterisks indicate statistical significance at *p* < 0.05 and *p* < 0.01, respectively. Statistical analyses were performed with one-way analysis of variance (ANOVA) and Dunnett’s multiple comparison test. The part of the image was created with BIORENDER

We investigated whether the PGAM1-Chk1 interaction might be involved in the oncogenic Ras-induced senescence (OIS) of human cells on the basis of our previous findings that oncogenic stimuli provoked this interaction in cancer cells.^23^ PGAM1, one of two isoforms of PGAM, is expressed predominantly in human primary fibroblasts (Supplementary Fig. 1a).^23^ For this purpose, we utilized the NanoLuc Binary Technology (NanoBiT) system to detect the PGAM1-Chk1 interaction (Fig. 1a). LgBiT-PGAM1 and Chk1-SmBiT were introduced into senescence-inducible human fibroblasts (MCR5-RasER). The growth curves and senescence of these cells are quite similar to those of the controls (Supplementary Fig. 1b). After 4-hydroxy tamoxifen treatment, several features of senescence were initiated, i.e., p16^Ink4a^ accumulation, a decrease in Lamin B1, an increase in γH2AX foci, a marker of DNA damage, and positive staining for senescence-associated beta-galactosidase (SA-β Gal) (Fig. 1b–d). Consistent with previous reports, the protein level of the tumor suppressor p53 increased immediately after oncogenic Ras induction, followed by a partial reduction at Days 8 and 16.^25^ Compared with non-SnCs, these SnCs presented enhanced glycolytic features (Fig. 1d, e).^14^ In this setting, the PGAM1-Chk1 interaction is enhanced in SnCs, as shown by the NanoBiT system (Fig. 1f). An immunoprecipitation assay also revealed increased interactions between endogenous PGAM1 and Chk1 both in OIS (Supplementary Fig. 1c) and in DNA damage-induced senescence (Supplementary Fig. 1d–f) of IMR90 cells, whereas Chk2 kinase, which is structurally unrelated yet functionally overlapping with Chk1, was not coimmunoprecipitated with PGAM1 in SnCs (Supplementary Fig. 1c) or cancerous cells.^23^ Thus, SnCs exhibit increased glycolysis along with increased binding of PGAM1-Chk1.

Ablation of the PGAM1-Chk1 interaction by Nutlin 3a, initially identified as an antagonist of p53-Mdm2 binding, attenuates the Warburg effect in cancer cells.^22^ In addition to Nutlin 3a, its optical isomer Nutlin 3b also blocks the PGAM1-Chk1 interaction in human cancer cells (Fig. 1g and Supplementary Fig. 1g), whereas Nutlin 3b scarcely inhibits p53-Mdm2 binding (Supplementary Fig. 1h).^24^ Both Nutlin 3a and 3b preserve the enzymatic activity of PGAM (Supplementary Fig. 1i).^23^ However, both Nutlin 3a and 3b effectively inhibited PGAM1-Chk1 binding and glycolytic activity in stress-induced or oncogene-induced SnCs (Fig. 1h–j and Supplementary Fig. 1j–m). Thus, although SnCs suffer permanent cell cycle arrest, they exhibit pseudo-Warburg features, which are suppressed by the inhibition of the PGAM1-Chk1 interaction.

### Blockade of the PGAM1-Chk1 interaction by Nutlin 3b induced senolysis and improved physiological aging

**Fig. 2:**
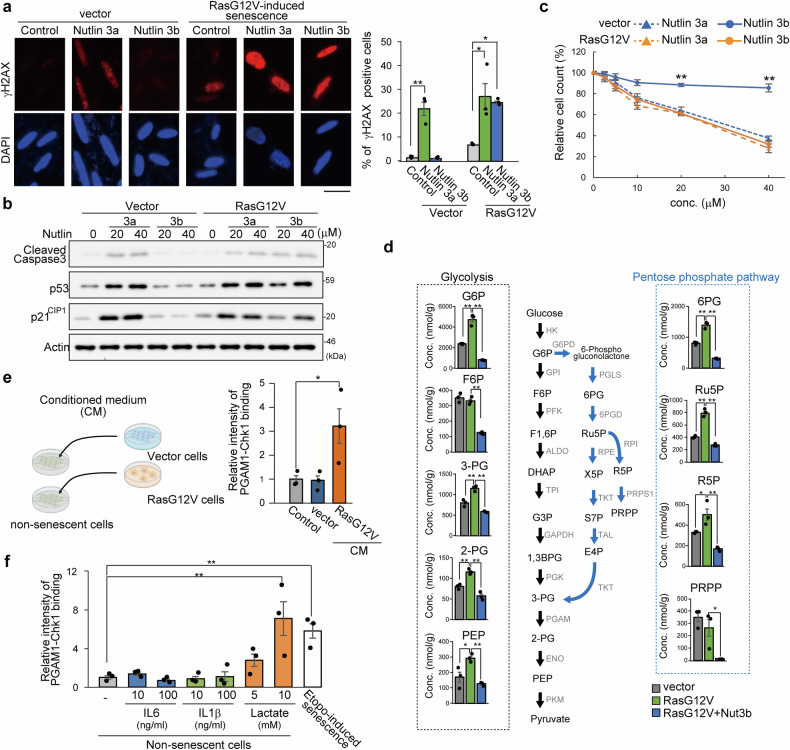
Abrogation of the PGAM1-Chk1 interaction eliminated senescent cells. **a**–**c** Senolytic effect of Nutlin 3b. Ras G12V-expressing senescent IMR90 cells were prepared. **a** γH2AX staining of control and SnCs. The indicated cells were treated with Nutlin 3a or 3b. The ratio of γH2AX foci-positive cells is shown in the right panel. **b** Cleavage of caspase 3, p53, and p21^CIP1^ in stress-induced senescent IMR90 cells treated with Nutlin 3a or 3b. The data represent two independent experiments. **c** Relative counts of senescent and control cells under Nutlin 3a or 3b treatment. The control and SnCs were treated with Nutlin 3a or 3b at different concentrations (0–40 μM) for 96 h. The blue and orange lines indicate the control and SnCs, respectively. Triangle; Nutlin 3a, Circle; Nutlin 3b. (*n* = 3, biological replicates). **d** Metabolomic analysis of control and SnCs with or without Nutlin 3b treatment. Metabolites in the glycolytic pathway are shown in black boxes, including G6P glucose-6-phosphate, F6P fructose 6-phosphate, 3-PG 3-phosphoglyceric acid, 2-PG 2-phosphoglyceric acid, and PEP phosphoenolpyruvate. Metabolites in the pentose phosphate pathway (PPP) are shown in the blue box, including 6GP 6-phospho-gluconate, Ru5P ribulose 5-phosphate, R5P ribose 5-phosphate, and PRPP phosphoribosyl diphosphate. **e** Effects of CM on the PGAM1-Chk1 interaction. Conditioned medium was collected from either control or SnCs. IMR90 cells expressing LgBiT-PGAM1 or Chk1-SmBiT were exposed to each conditioned medium. A NanoBit assay was performed to detect the interaction between PGAM and Chk1 (*n* = 3, biological replicates). This image was created with BIORENDER. **f** The impact of lactate on the PGAM1-Chk1 interaction. IMR90 cells expressing LgBiT-PGAM1 and Chk1-SmBiT were exposed to increasing amounts of IL6, IL1β, or lactate. The NanoBit signal was normalized against the protein content (*n* = 3, biological replicates). The data represent the means ± SEMs of three biological replicates. Single (*) and double (**) asterisks indicate statistical significance at *p* < 0.05 and *p* < 0.01, respectively. Statistical analyses were performed with one-way analysis of variance (ANOVA) and Dunnett’s multiple comparison test

Interestingly, 2-deoxy-D-glucose (2-DG), a glycolytic inhibitor that functions as a glucose analog, increased the number of γH2AX foci in SnCs (Supplementary Fig. 2a), which is consistent with the notion that increased glycolysis is essential for DNA integrity and repair.^26^ We observed that Nutlin 3a downregulated glycolysis in both non-SnCs and SnCs, whereas Nutlin 3b did so in SnCs but not in non-SnCs (Fig. 1i, Supplementary Fig. 1k, m and 2b). In this context, Nutlin 3a upregulated γH2AX foci and activated p53, followed by an apoptotic response in both non-SnCs and SnCs (Fig. 2a–c, and Supplementary Fig. 2c). In contrast, p53, γH2AX foci, and the apoptotic response were scarcely activated by Nutlin 3b in non-SnCs, whereas Nutlin 3b induced an apoptotic response in OIS cells (Fig. 2a–c, and Supplementary Fig. 2c) and in DNA damage-induced SnCs (Supplementary Fig. 2d, e). Thus, Nutlin 3b restored the viability of early-passaged cells, in contrast to Nutlin 3a. QVD-Oph, an apoptosis inhibitor, but not necrostatin-1, a necrosis inhibitor, restored the viability of SnCs under Nutlin 3b treatment (Supplementary Fig. 2f). These findings imply that Nutlin 3b, but not Nutlin 3a, exerts senolytic activity in vitro, comparable to that of senolytic ABT263 (an antagonist of the Bcl-2 family) (Supplementary Fig. 2g).^18^ In addition, Nutlin 3b effectively induced senolysis both in irradiation (IR)-induced and replicative SnCs of fibroblasts (Supplementary Fig. 2h–p). However, the SnCs provoked by inactivation of PGAM1 or Chk1 restored their viability under Nutlin 3b treatment (Supplementary Fig. 3a–j), suggesting that Nutlin 3b functions as a senolytic through the PGAM-Chk1 axis. As p53 inactivation restored the viability of Nutlin 3a-treated young cells but not of SnCs under Nutlin 3b treatment, Nutlin 3b induced senolysis in a p53-independent manner (Supplementary Fig. 3k–m). Interestingly, Nutlin 3a also provoked p53-independent apoptosis of SnCs, suggesting that the maintenance of the PGAM-Chk1 interaction is crucial for the survival of SnCs. Owing to the complexity and heterogeneity of SnCs in vivo,^27^ we evaluated the effects of Nutlin 3b on SnCs of different cell types. Senescent macrophages were largely vulnerable to senolytic Nutlin 3b (Supplementary Fig. 4i–l), whereas senescent vascular endothelial cells, EA.hy926 cells or HUVECs, were not (Supplementary Fig. 4a–h). These findings suggest that Nutlin-3b exerts senolytic effects in a cell context-dependent manner.

To further evaluate the metabolic impact of Nutlin 3b in SnCs, we performed metabolomic analysis. We found that the upregulation of glycolysis-related metabolites in SnCs was reversed by Nutlin 3b treatment (Fig. 2d). The pentose phosphate pathway (PPP), a branching pathway from glycolysis, involves the production of NADPH and nucleotide pools, which are essential for DNA replication and repair.^28^ In addition to glycolytic inhibition, Nutlin 3b effectively downregulated PPP-relevant metabolites, which are precursors for purine/pyrimidine synthesis (Fig. 2d), suggesting that Nutlin 3b causes exhaustion of nucleotide pools. Together, these results are in line with a senolytic effect of Nutlin 3b that is not dependent on Mdm2-p53 but is dependent on disruption of the PGAM1/Chk1 interaction and metabolic effects.

Impaired mitochondria are one of the hallmarks of aging and senescence.^29^ Given that mitochondrial dysfunction increases glycolysis, we investigated whether impaired mitochondria play a causal role in the PGAM1-Chk1 interaction. Human primary cells were treated with inhibitors of the mitochondrial electron transport chain, rotenone or antimycin. However, the PGAM1-Chk1 interaction was not affected by mitochondrial inhibition (Supplementary Fig. 5a). To further explore the physiological relevance of enhanced glycolysis in senescence, we prepared conditioned medium from SnCs and non-SnCs and replaced the medium of primary fibroblasts with medium from early-passage cells (Fig. 2e). The PGAM1-Chk1 interaction was augmented by conditioned medium from SnCs but not by that from non-SnCs. As SnCs secrete inflammatory factors, we evaluated the effects of IL6 and IL1β, which are representative SASP factors, on the PGAM1-Chk1 interaction. The addition of IL6 or IL1β proteins to the medium at physiological concentrations, comparable to those secreted by SnCs, had no significant effect on the PGAM1-Chk1 interaction (Fig. 2f and Supplementary Fig. 5b). Further analysis of other SASP factors, including chemokines (CCL2 and CXCL12), growth factors (AREG and OPG), and glycolytic modulators (IGFBP1-6), did not reveal a positive effect on PGAM1-Chk1 binding (Supplementary Fig. 5c). On the other hand, consistent with the increase in lactate in the medium from SnCs (Fig. 1e and Supplementary Fig. 5d), lactate treatment of primary human fibroblasts increased the PGAM1-Chk1 interaction (Fig. 2f). Thus, SnCs exhibit intriguing correlations with the PGAM1-Chk1 interaction, with glycolytic upregulation leading to lactate production.

To examine the in vivo effect of Nutlin 3b, we performed a toxicity assessment. The IC50 of Nutlin 3b in vitro was expected to be 69.51 μM (Supplementary Fig. 6a). We confirmed that Nutlin 3b does not interfere with the DNA-sensing immune pathway in standard cells (Supplementary Fig. 6b). A comparison of the effects of short-term treatment with Nutlin 3a and 3b at high dosages (100 or 200 mg/kg) in young mice revealed lethal and harmful toxicity of Nutlin 3a (Supplementary Fig. 6c–g), whereas one-shot injection of Nutlin 3b at 50 or 100 mg/kg preserved body weight and blood parameters (Supplementary Fig. 6h–j). By a quantitative method to detect the in vivo pharmacokinetics of Nutlin 3b via liquid chromatography–tandem mass spectrometry (LC‒MS/MS) (Supplementary Fig. 7a, b), we estimated the in vivo half-life of plasma Nutlin 3b to be 1.1 h (Supplementary Fig. 7c). The peak concentration of Nutlin 3b in the liver was much greater than that in the plasma, while the obvious peak of Nutlin 3b was not detected in the brain (Supplementary Fig. 7d, e). The long-term toxicity assessment was performed by weekly injection of Nutlin 3b at 11.62 mg/kg for three months (Supplementary Fig. 8a). Blood tests and body weights confirmed the preserved health of the young mice (Supplementary Fig. 8b–d). There was no apparent inflammation or degeneration in the liver, kidney, lung, heart or brain in either group (Supplementary Fig. 8e–g).

**Fig. 3:**
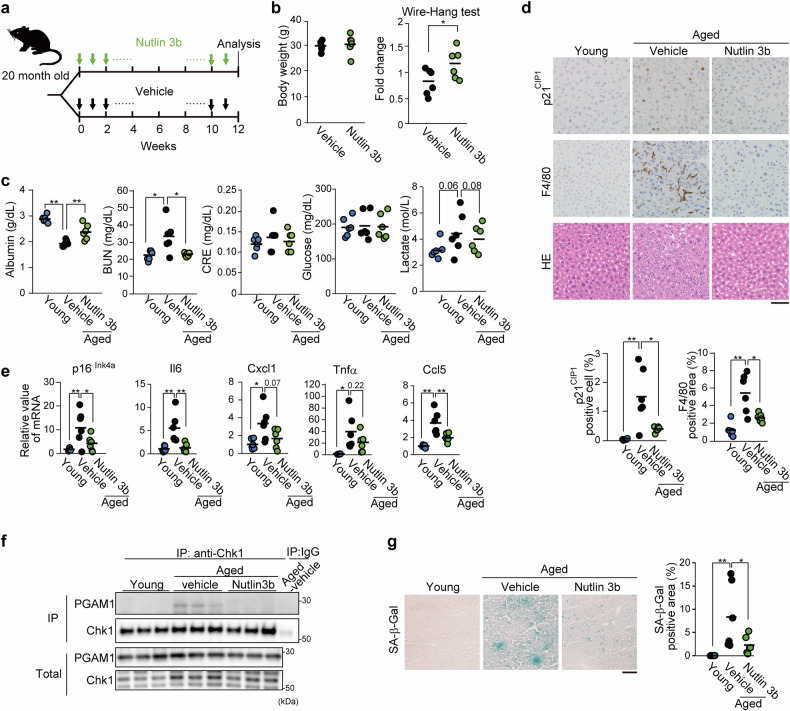
Aging-related dysfunctions in vivo were alleviated by Nutlin 3b treatment. Administration of Nutlin 3b to aged mice. Twenty-month-old female mice were treated with weekly *i.p*. injections of Nutlin 3b for three months. One week later, the physiological impact of Nutlin 3b in the mice was assessed (*n* = 6, mice). **a** Schematic diagram of the in vivo protocol for Nutlin-3b treatment. **b** Wire-hang test for the evaluation of muscle strength. **c** Blood parameters of young or aged mice with or without Nutlin-3b treatment. **d** Immunohistochemical examination of the liver for p21 ^CIP1^ and F4/80 staining. Representative images of stained samples from the indicated mice are shown (upper panels). The ratio of p21 ^CIP1^- or F4/80-positive cells was assessed (lower panel). The bar indicates 50 μm. **e** Comparison of the mRNA levels of p16^Ink4a^ and SASP factors (Il6, Cxcl1, Tnfα, and Ccl5). Liver extracts from the indicated mice were analyzed via RT‒PCR. **f** Immunoprecipitation was used to detect PGAM1-Chk1 interactions in aged livers with or without Nutlin 3b treatment and in young livers. **g** Evaluation of SA-β GAL staining in aged livers. Representative pictures (left panels) and positivity of SA-β GAL staining in the indicated samples. Each group comprised female (*n* = 3) and male (*n* = 3) mice. The data represent the means ± SEMs. Single (*) and double (**) asterisks indicate statistical significance at *p* < 0.05 and *p* < 0.01, respectively. Statistical analyses were performed via unpaired Student’s two-tailed *t* tests (**b**) or one-way analysis of variance (ANOVA) and Dunnett’s multiple comparison test (**c**–**e** and **g**)

Next, we evaluated the in vivo effect of Nutlin 3b in aged mice (female in Fig. 3 and male in Supplementary Fig. 9). Twenty-month-old mice were injected *i.p*. with Nutlin 3b weekly for three months (Fig. 3a). The body weights of the control and Nutlin 3b-treated groups were comparable (Fig. 3b and Supplementary Fig. 9a). As blood glucose homeostasis is closely linked to body weight and adiposity during aging, elevated glucose levels are an indicator of mortality in elderly human populations.^30^ In contrast, this is not the case in aged mice (Fig. 3c),^30^ whereas Nutlin 3b mildly ameliorated blood lactate levels (*p* = 0.08) (Fig. 3c). Thus, it is still not clear whether blood glucose levels could be effective biomarkers of aging. Strikingly, Nutlin 3b improved physiological parameters in aged mice, including muscle strength (wire-hang test), plasma albumin, and blood urea nitrogen (BUN, a renal marker), which are also known as biomarkers for frailty^31,32^ (Fig. 3b, c, and Supplementary Fig. 9a, b), one of the representative indices for biological aging in humans.

Histochemical analysis of the liver, which is responsible for albumin production and lactate clearance,^33^ revealed that p21^CIP1^-positive cell accumulation in aged Nutlin 3b-treated aged mice was significantly reduced (Fig. 3d), accompanied by reduced p16^Ink4a^ levels (Fig. 3e). Moreover, the number of F4/80-positive cells, indicating infiltration of macrophages in aged tissues, was also reduced by Nutlin 3b treatment, which was consistent with the decreases in several SASP factors (Il6, Cxcl1, Tnfα and Ccl5) (Fig. 3d, e and Supplementary Fig. 9c). We observed greater PGAM1-Chk1 interaction and positive SA-β GAL staining in aged livers than in young mouse livers, and these effects were suppressed by Nutlin 3b treatment (Fig. 3f, g). Overall, blockade of the PGAM1-Chk1 interaction with Nutlin 3b induced senolytic effects in vitro and in vivo.

### The HIF-2α protein accumulates in senescent cells but is downregulated by inhibition of the PGAM1-Chk1 interaction

**Fig. 4:**
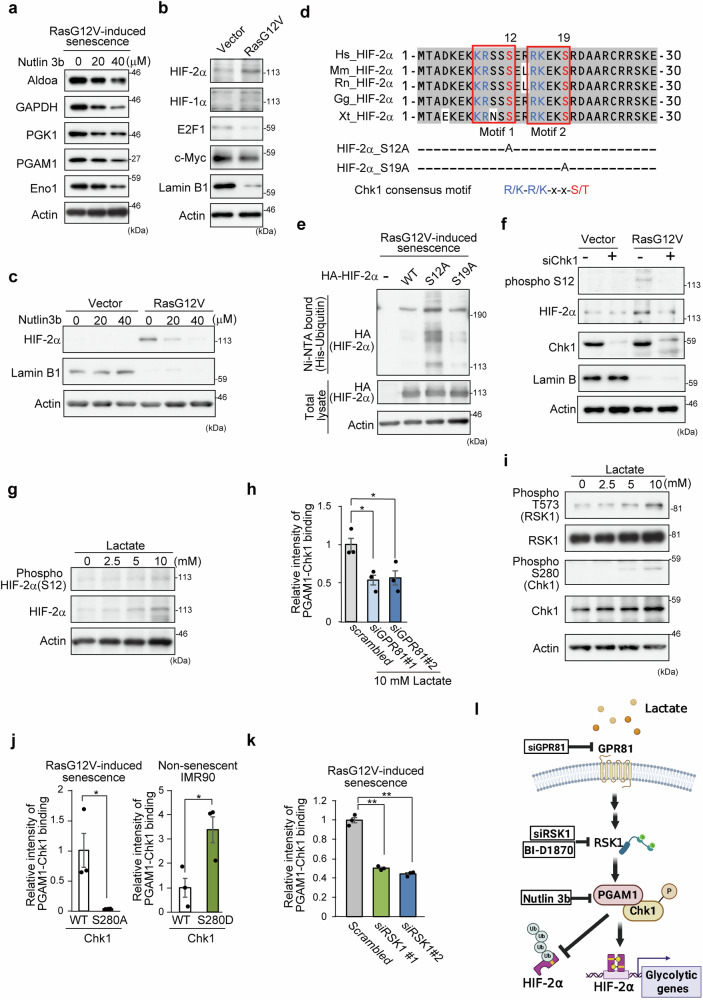
The HIF-2α protein accumulates in senescent cells and is downregulated by inhibiting the PGAM1-Chk1 interaction. **a** Protein levels of glycolytic enzymes after Nutlin 3b treatment. SnCs induced by oncogenic Ras were exposed to different concentrations of Nutlin 3b (0–40 μM). Aldo aldolase, GAPDH glyceraldehyde 3-phosphate dehydrogenase, PGK phosphoglycerate kinase, ENO enolase. **b** Transcription factors for glycolysis were evaluated in control and Ras-induced SnCs. Western blot with antibodies against HIF-2α, HIF-1α, E2F1, c-Myc and Lamin B1 in the indicated cells. **c** Effect of Nutlin 3b on the HIF-2α protein. The protein levels of HIF-2α are shown via western blotting. Senescent and control cells were treated with Nutlin 3b for 48 h. **d** Diagram of the amino-terminal sequence of HIF-2α in several organisms. Conserved amino acids are highlighted. Conserved motifs comprise the consensus sequence for phosphorylation by Chk1 (K/R-K/R-x-x-S/T), which is surrounded by a red box. Motifs 1 and 2 include Ser-12 and Ser-19, respectively. Hs *Homo sapiens*, Mm *Mus musculus*, Rn *Rattus norvegicus*, Gg *Gallus gallus*, Xt *Xenopus tropicalis*. **e** Ubiquitination assay for the HIF-2α protein. Extracts were collected from SnCs expressing His-ubiquitin and various versions of HA-HIF-2α. Purification on Ni-NTA agarose beads was followed by immunoblotting with an HA antibody. The data are representative of two independent experiments. **f** Phosphorylation status of HIF-2α at Ser12 in SnCs. Control or SnCs were transfected with Chk1 siRNA. The resulting extracts were probed with an anti-phospho-S12-HIF-2α antibody. **g** The impact of lactate on HIF-2α status. IMR90 cells were exposed to increasing amounts of lactate. Western blotting was performed. **h** NanoBit assay for assessing the PGAM1-Chk1 interaction after ablation of GPR81 (*n* = 3, biological replicates). IMR90 cells expressing LgBiT-PGAM1 and Chk1-SmBiT were transfected with the indicated siRNAs. **i** Evaluation of Chk1 and Rsk1 activation after lactate treatment by western blotting. Antibodies against phospho-Chk1-S280 and phospho-RSK1-Thr573 were applied. **j** Impact of the Chk1-S280 status on the PGAM1-Chk1 interaction. Several retroviruses bearing LgBiT-PGAM1, Chk1WT-SmBiT, or Chk1-SmBiT (S280A or S280D) mutants have been produced. The indicated retroviruses were introduced into IMR90 cells. Senescence was induced by Ras G12V expression. NanoBit assays detected interactions between PGAM1 and Chk1-WT or Chk1- S280A in SnCs (left panel) and between PGAM1 and Chk1-WT or Chk1- S280D in non-SnCs (right panel) (*n* = 3, biological replicates). **k** Effect of RSK1 knockdown on the PGAM1-Chk1 interaction in oncogene-induced SnCs. A NanoBit assay was performed to detect the interaction between PGAM1 and Chk1 (*n* = 3, biological replicates). **l** A schematic diagram of the mechanism that regulates the interaction between PGAM1 and Chk1. This image was created with BIORENDER. The data represent the means ± SEMs. Single (*) and double (**) asterisks indicate statistical significance at *p* < 0.05 and *p* < 0.01, respectively. Statistical analyses were performed via unpaired Student’s two-tailed *t* tests (**j**) or one-way analysis of variance (ANOVA) and Dunnett’s multiple comparison test (**h** and **k**)

To gain mechanistic insight into the metabolic and senolytic effects of Nutlin 3b, profiles of glycolytic enzymes were assessed. In Nutlin 3b-treated SnCs, the protein and mRNA levels of glycolytic enzymes were decreased (Fig. 4a and Supplementary Fig. 10a). Next, we evaluated the protein profiles of several glycolytic regulators, c-myc, E2F1, HIF-1α, and HIF-2α, in SnCs.^10,34^ Among these proteins, the level of HIF-2α protein increased prominently in SnCs, whereas the other proteins, namely, HIF-1α, E2F1, and c-myc, were rather constant or reduced during senescence (Fig. 4b). Strikingly, Nutlin 3b effectively downregulated HIF-2α protein expression in SnCs but not in the control (Fig. 4c and Supplementary Fig. 10b). The proteasomal inhibitor MG132 restored HIF-2α protein levels in Nutlin 3b-treated SnCs, but its mRNA levels were less affected (Supplementary Fig. 10c, d), suggesting the possible involvement of posttranscriptional regulation of the HIF-2α protein during Nutlin 3b treatment.

Posttranscriptional modifications, including phosphorylation, of target proteins render them susceptible to ubiquitination. Notably, the consensus sequences for phosphorylation by Chk1 (K/R-K/R-x-x-S/T) are conserved and located in the N-terminus of HIF-2α. These sequences, designated Motifs 1 and 2, involve Ser-12 and Ser-19, respectively (Fig. 4d). Ser12 of HIF-2α is reportedly phosphorylated in vitro, according to the database of global phosphoproteins (https://www.phosphosite.org). We found that the S12A mutant of HIF-2α, but not S19A, was very vulnerable to ubiquitination in SnCs (Fig. 4e and Supplementary Fig. 10e). We generated an anti-phospho antibody against Ser-12 of HIF-2α (Supplementary Fig. 10f), which detected its phosphorylation of Ser-12 in SnCs in a Chk1-dependent manner (Fig. 4f). These data suggest that the stability of the HIF-2α protein is a target of the PGAM1-Chk1 interaction during senescence.

Consistent with the finding that lactate facilitates the PGAM1-Chk1 interaction (Fig. 2f), lactate-treated human primary cells accumulated HIF-2α, which was phosphorylated at Ser12 (Fig. 4g). Lactate plays multiple roles, including energy production, the induction of an acidic environment, the stimulation of tumor growth, acidification via its uptake into cells, the production of byproducts of glutaminolysis, and signal transduction.^35^ However, inactivation of the lactate uptake transporter MCT4 or glutaminase 1 (GLS1) in SnCs did not affect the PGAM-Chk1-NanoBiT values (Supplementary Fig. 11a–c). The cellular membraneous apparatuses GPR81 and GPR132 are reported as cell-surface receptors for lactate.^35,36^ Only GPR81 was upregulated in SnCs (Supplementary Fig. 11d), and its knockdown abolished the lactate-induced increase in the PGAM1-Chk1 interaction (Fig. 4h and Supplementary Fig. 11e). GPR81 signaling is involved in oncogenic modules, including the Ras pathway.^37^ In both senescent and lactate-treated cells, RSK1 kinase, which is downstream of the Ras pathway, is activated, accompanied by the phosphorylation of Ser280 in Chk1, a known target of RSK1^38^ (Fig. 4i and Supplementary Fig. 11f). Our mutagenesis analysis suggested that the S280A mutation in Chk1 abolished PGAM-Chk1 binding, whereas the S280D mutation, a mimic of phosphorylation, accelerated it (Fig. 4j). In SnCs, inactivation of RSK1 by a specific siRNA or its chemical inhibitor, BI D1870, attenuated the PGAM1-Chk1 interaction and reduced the HIF-2α protein level and phosphorylation of Ser280 in Chk1 (Fig. 4k and Supplementary Fig. 11g, h). These results indicate that the phosphorylation of S280 in Chk1 by the RSK1 pathway, which is downstream of Ras and GPR81 signaling, is required for the binding of PGAM1 and Chk1 (Fig. 4l).

### PGAM1-Chk1 binding antagonists suppress FOXM1 during senescence

**Fig. 5:**
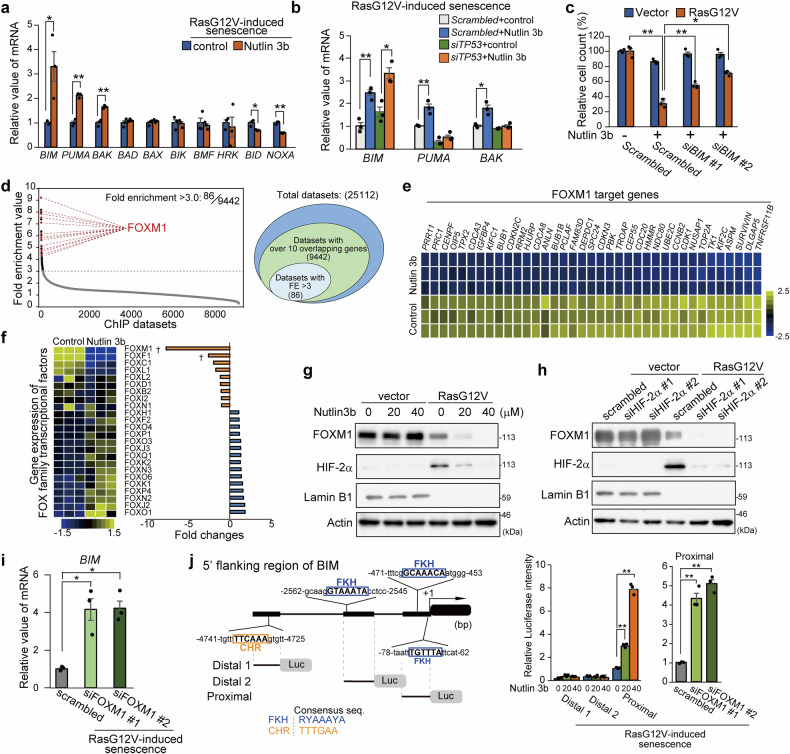
A PGAM1-Chk1 antagonist suppresses FOXM1 during senescence. **a**, **b** Assessment of the proapoptotic BH family in SnCs after Nutlin 3b treatment. **a** RT‒PCR analysis of SnCs with or without Nutlin 3b. (*n* = 3, biological replicates). **b** p53 siRNA-transfected SnCs were exposed to Nutlin 3b. RT‒PCR analysis of the indicated cells. **c** Knockdown of BIM in SnCs. SnCs were transfected with the indicated siRNA, siBIM, or scramble control RNA. The transfectants were then subjected to Nutlin 3b treatment. **d**–**f** Comparison of the results of the microarray analysis between the control and Nutlin 3b-treated SnCs (*n* = 3, biological replicates). **d** Fold enrichment analysis by comparison between 1168 genes downregulated by Nutlin 3b in SnCs and 9442 datasets of chromatin immunoprecipitation (ChIP) information reported in the National Center for Biotechnology Information (NCBI), European Bioinformatics Institute (EBI) and DNA Data Bank of Japan (DDBJ). Eighty-six of the 9442 datasets displayed high enrichment (>3-fold). Twenty-one datasets for FOXM1 are shown in red (left panel). **e** Heatmap of 37 FOXM1 target genes downregulated by Nutlin 3b. SnCs with or without Nutlin 3b were compared. **f** Gene expression of the FOX family in SnCs after Nutlin 3b treatment, as determined via microarray data. Left panel, heatmap analysis of the FOX family. Right panel: Red and blue bars indicate reduced and increased expression, respectively. Dagger (†) indicates |FC|≥2 and Lpe. *P* < 0.05 is presented in Supplementary Fig. 12c. Evaluation of FOXM1 protein levels in SnCs after Nutlin 3b treatment (**g**) and after transfection with HIF-2α siRNA (**h**). The data are representative of two independent experiments. **i** Evaluation of BIM mRNA in SnCs after FOXM1 knockdown. FOXM1 siRNA was transfected into SnCs (*n* = 3, biological replicates). **j** Schematic diagram of the promoter region in the human BIM gene. Several cis-elements for FKH are shown. The indicated regions were subsequently cloned and inserted into luciferase reporter plasmids (left lower panel). Luciferase reporter assays were performed in SnCs after Nutlin 3b treatment (middle panel). A reporter assay for the proximal region was conducted in SnCs after FOXM1 knockdown (right panel) (*n* = 3, biological replicates). The data represent the means ± SEMs. Single (*) and double (**) asterisks indicate statistical significance at *p* < 0.05 and *p* < 0.01, respectively. Statistical analyses were performed via unpaired Student’s two-tailed *t* test (**a**) or one-way analysis of variance (ANOVA) and Dunnett’s multiple comparison test (**b**, **c**, **i** and **j**)

To elucidate the apoptotic factors involved in senolysis by Nutlin 3b, we explored the profiles of 10 proapoptotic BH proteins in Nutlin 3b-treated SnCs. Among the BH genes tested, in addition to PUMA and BAK, BIM was the most highly upregulated by Nutlin 3b in SnCs but not in controls (Fig. 5a and Supplementary Fig. 12a). As p53 is activated in SnCs by Nutlin 3b (Fig. 2b), we suspect that the transcriptional activity of p53 is responsible for the upregulation of these proapoptotic BH proteins by Nutlin 3b. PUMA and BAK, known p53 targets,^39^ were suppressed by p53 siRNA transfection in Nutlin 3b-treated SnCs while BIM were not (Fig. 5b). Moreover, siRNA against BIM partially restored the survival of Nutlin 3b-treated SnCs, indicating the causal involvement of BIM in Nutlin 3b-mediated senolysis (Fig. 5c and Supplementary Fig. 12b). Thus, BIM, which is not regulated by p53, is induced by PGAM-Chk1 binding inhibition by Nutlin 3b in SnCs.

To elucidate the molecular mechanism of senolysis by the inhibition of PGAM1-Chk1 binding, transcriptomic analysis was performed (Supplementary Fig. 12c). A total of 168 genes downregulated >2-fold by Nutlin 3b in SnCs (Supplementary Fig. 12d) were compared for enrichment analysis with the ChIP (chromatin immunoprecipitation) information reported in NCBI, EBI and DDBJ. The latter information comprises 25,112 ChIP datasets involving 903 antigens (transcriptional and epigenetic factors). Consequently, 9442 ChIP datasets revealed more than 10 overlapping genes, and 86 datasets showed high enrichment >3-fold (Fig. 5d). Among the top 20 genes in these 86 datasets, 11 contained ChIP data analyzed with FOXM1 as the antigen, which presented the highest enrichment score (9.19) (Fig. 5d, Supplementary Table 1). Consistently, among the 150 genes most downregulated (<−8.1-fold) by Nutlin 3b, 24.6% (37 genes) were identical to FOXM1 targets, including cell cycle regulators and components of the mitotic apparatus (Fig. 5e, Supplementary Table 2).^40^ In addition, Nutlin 3b also downregulated DNA repair machinery regulated by FOXM1, including XRCC1, BRCA1, TOP2A, EXO1 and Survivin (also known as antiapoptotic factor^41^), in SnCs but not in nonsenescent controls (Supplementary Fig. 12e, f). Among the 24 FOX family genes, FOXM1 mRNA was the most drastically downregulated by Nutlin 3b (Fig. 5f). Gene set enrichment analysis (GSEA) of the microarray data revealed that Nutlin 3b most significantly suppressed FOXM1 targets (FDR *q* < 0.001) compared with the other FOX family genes (Supplementary Fig. 12g, h). Nutlin 3b or inactivation of HIF-2α downregulated the FOXM1 protein in SnCs but not in control cells (Fig. 5g, h). Finally, a chromatin immunoprecipitation (ChIP)-qPCR assay was performed. HIf-2α binding to the FoxM1 promoter was enhanced in SnCs but was suppressed by Nutlin 3b (Supplementary Fig. 13c). These data suggest that the HIF-2α/FOXM1 axis actively operates in SnCs. Consistently, Nutlin 3b downregulated the FOXM1 protein, which accumulated in the livers of aged mice (Supplementary Fig. 13a, b).

### FOXM1 inactivation induced the expression of the proapoptotic BIM protein during treatment with the senolytic PGAM1-Chk1 antagonist

However, little is known about how BIM is regulated in SnCs. We found that FOXM1 knockdown upregulated BIM mRNA in SnCs (Fig. 5i and Supplementary Fig. 13d). Notably, several Forkhead-responsive elements (FKH, RYAAAYA) are located in the promotor of BIM at −2550, −450, and −70 bp upstream (Fig. 5j). In addition, the cell cycle gene homology region (CHR, TTTGAA) is located at −4730 bp and is recognized by the Dream complex, which contains E2F4, p130, or FOXM1. Several regions in this promoter with these elements were designated distal1 (−5197 ~ −4397 bp), distal2 (−2563 ~ −1560 bp) and proximal (−925 ~ −1 bp) and were evaluated via a luciferase reporter assay in SnCs (Fig. 5j). The reporter in the proximal region, but not at distal1 or distal2, was transactivated in SnCs by Nutlin 3b (Fig. 5j). Similarly, FOXM1 siRNA enhanced the reporter activity of the proximal region in SnCs (Fig. 5j). Abolishment of the FKH regions in the proximal promoter significantly enhanced reporter activity in SnCs (Supplementary Fig. 13e). Overall, proapoptotic BIM, which is suppressed by FOXM1 during senescence, is provoked by senolytic PGAM1-Chk1 inhibition.

### Aged FOXM1-accumulating tissues, such as the liver, lung and kidney, are targets of the senolytic Nutlin 3b

**Fig. 6:**
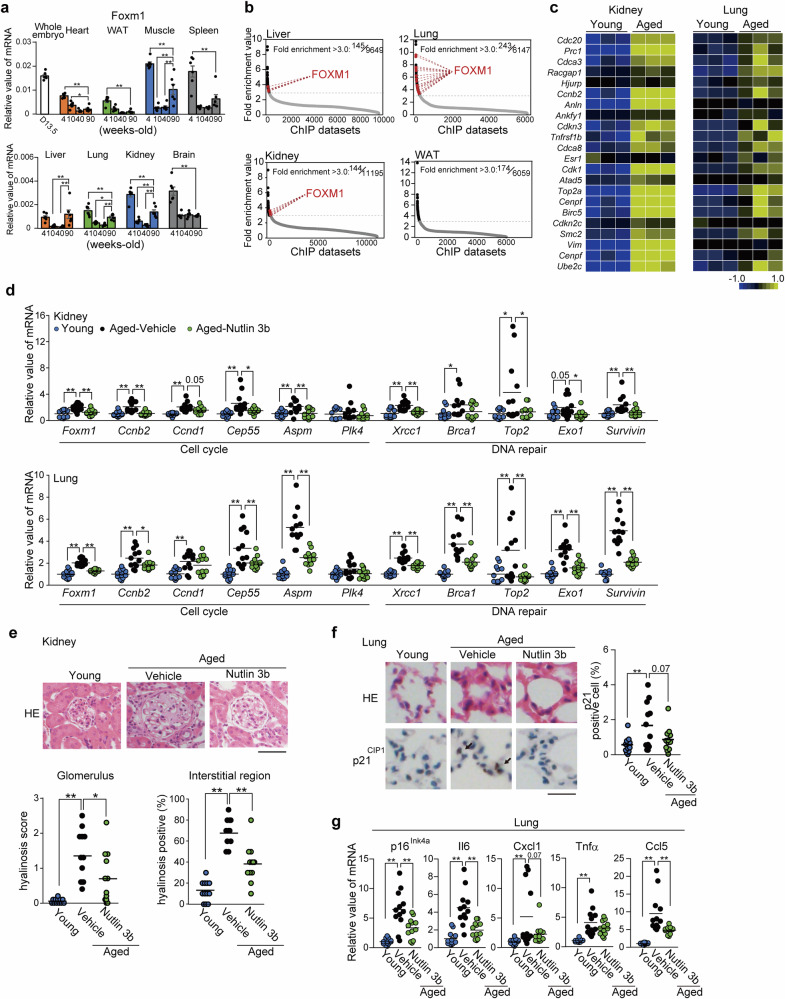
In vivo FOXM1 accumulation in aged tissues is targeted by Nutlin 3b. **a** In vivo profiles of FOXM1 mRNA in several tissues of 4-, 10-, 40- and 90-week-old mice, in addition to embryos on day 13.5. The indicated tissues were collected for RNA extraction. **b** Fold enrichment analysis comparing upregulated genes in 90-week-old mice (liver: 1039; kidney: 1882; lung: 386; and WAT: 385) and ChIP datasets (liver: 9649; kidney: 11,195; lung: 6147; and WAT: 6059). Comparative transcriptomic analysis of the indicated tissues, liver, lung, kidney, and WAT, was performed between 10-week-old and 90-week-old mice. **c** Heatmap analysis of FOXM1 target genes in the kidney and lung (left and right panels) of young (10-week-old) and aged (90-week-old) mice. **d** Assessment of FOXM1 target genes involved in the cell cycle and DNA repair genes in aged kidneys and lungs (upper and lower panels, respectively) after Nutlin 3b treatment via RT‒PCR analysis. Tissues were collected from young (10-week-old), aged (90-week-old) and Nutlin 3b-treated aged mice. **e** The impact of Nutlin-3b on aged kidneys. Representative pictures of the glomerulus after Nutlin 3b treatment (upper panels). Samples from young, aged and Nutlin 3b-treated aged mice. The bar indicates 50 μm. Hyalinosis scores were assessed in aged mice with or without Nutlin 3b treatment. Glomerulus region (lower left) and interstitial region (lower right). **f** Immunohistochemical examination of the lung for p21 ^CIP1^. Representative images of stained samples from the indicated mice are shown (left panels). The bar indicates 20 μm. The ratio of p21 ^CIP1^-positive cells was assessed (right panel). **g** Comparison of the mRNA levels of p16^Ink4a^ and SASP factors (Il6, Tnfα, Cxcl1, and Ccl5). Lung extracts from the indicated mice were analyzed via RT‒PCR. The data represent the means ± SEMs. Single (*) and double (**) asterisks indicate statistical significance at *p* < 0.05 and *p* < 0.01, respectively. Statistical analyses were performed via one-way analysis of variance (ANOVA) and Dunnett’s multiple comparison test

Next, we evaluated in vivo profiles of Foxm1 mRNA in several tissues (heart, WAT, muscle, spleen, liver, lung, kidney, and brain) at several stages of the life course in mice; from 4, 10, 40 to 90 weeks of age, in addition to embryos on day 13.5, p16^Ink4a^ levels were increased the most in the samples from all the tested tissues at 90 weeks of age (Supplementary Fig. 14a). Consistent with the notion that Foxm1 is a master regulator of cell proliferation,^42^ it was highly expressed in the embryo (Fig. 6a). In growing mice (4 weeks old), the Foxm1 mRNA level was also high, but its level gradually decreased in young and middle-aged mice (10 and 40 weeks old). However, in aged mice (90 weeks old), Foxm1 levels were significantly increased in the liver, lung, kidney, muscle and spleen but not in the heart, WAT or brain (Fig. 6a). Thus, Foxm1 mRNA in aged mice was increased compared with that in young and middle-aged mice in a tissue context-dependent manner. Next, comparative transcriptomic analysis was performed on several tissues (liver, lung, kidney, and WAT) from young and aged mice (10 and 90 weeks old, respectively). Heatmap analysis revealed enhanced SASP profiles in these old tissues (Supplementary Fig. 14b). Chromatin immunoprecipitation (ChIP) data (29,194 datasets) were utilized for enrichment analysis of these transcriptomic data. Among the transcription factors with greater than 3.0-fold enrichment (FEs), the fold enrichment analysis revealed overlapping molecules as the top 5 with high scores in the hit datasets of the tested tissues (liver, lung, kidney, and WAT) (Supplementary Fig. 14c). These factors include inflammation-relevant transcription factors, including RelA (a subunit of NF-κB), the STAT family, Spi1, and Runx1,^43^ which is consistent with the SASP property of SnCs. In these top 5 lists, Foxm1 is included in the aged liver, lung, and kidney but not in the WAT (Supplementary Fig. 14c). Foxm1 was detected as having high FE scores in samples from the liver, lung and kidney but not in those from the WAT (Fig. 6b). Consistently, heatmap analysis indicated that the expression of transcriptional targets of Foxm1 is upregulated in the aged liver, lung and kidney (Fig. 6c and Supplementary Fig. 15a). Interestingly, in aged tissues with Foxm1 activation (lung, liver, and kidney), several DNA repair machineries, known as targets of FOXM1, are upregulated; Survivin, Xrcc1, Brca1, Top2, and Exo1, which are effectively alleviated by Nutlin 3b treatment, similar to other senescent markers (Fig. 6d and Supplementary Fig. 15b). Thus, Nutlin 3b targets FOXM1-upregulated SnCs in vitro and tissues in vivo. Indeed, in addition to its effect on aged livers (Fig. 3d–g), Nutlin 3b alleviated dysfunctional damage in aged kidneys and lungs, where Foxm1 accumulated during aging (Fig. 6e–g). Consistent with improved blood BUN levels (Fig. 3c), Nutlin 3b relieved hyalination, a sign of renal sclerosis, SASP (Tnfα, Cxcl1, Ccl5) and the senescent marker p16^Ink4a^ in aged kidneys (Fig. 6e and Supplementary Fig. 15c). Nutlin 3b also alleviated SASP and p21 accumulation in aged lungs (Fig. 6f, g). Thus, Nutlin 3b is an effective senolytic approach against FOXM1-accumulating aged tissues in vivo.

### Chemical or genetic interference with the PGAM1-Chk1 interaction alleviates pulmonary fibrosis in mice

**Fig. 7:**
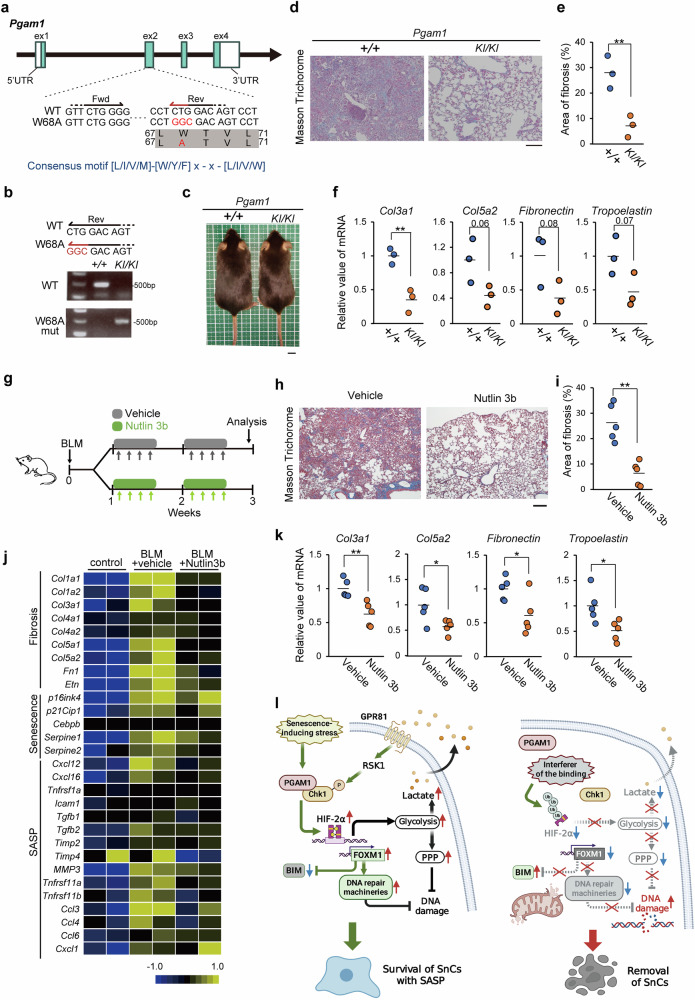
Chemical or genetic interference with PGAM1-Chk1 binding alleviated lung fibrosis in vivo. Generation of *Pgam1 W68A* knock-in (KI) mice via the CRISPR/Cas9 system (**a**–**c**). Bleomycin (BLM)-induced lung fibrosis was evaluated in *Pgam1* ^*KI/KI*^ mice (**d**–**f**). **a** Schematic diagram of *Pgam1* (exons 1–4) alleles; the Nutlin-consensus motif ([L/I/V/M]-[W/Y/F]-X-X- [L/I/V/M]) is located in exon 2. The *W68A* mutation was introduced via CRISPR/Cas9. **b** Genomic PCR was used to detect the *W68A* mutation in *Pgam1* ^*KI/KI*^ mice. **c** Representative images of *Pgam1* ^*+/+*^ and ^*KI/KI*^ mice. The bar indicates 1 cm. **d** Histological images of BLM-induced lung fibrosis in *Pgam1* ^*KI/KI*^ mice obtained via Masson’s trichrome (MT) staining. Lung tissues were collected on day 18 after 2.5 mg/kg BLM injection. The bar indicates 100 μm. **e** Evaluation of fibrotic areas in *Pgam1* ^*KI/KI*^ mice via MT staining. **f** Assessment of fibrotic markers in lungs from *Pgam1* ^*KI/KI*^ mice via RT‒PCR analysis. Administration of Nutlin 3b to a mouse model of lung fibrosis (**g**–**k**). BLM was intratracheally instilled into the lungs of 12-week-old CD1 mice, resulting in DNA damage-induced pulmonary fibrosis. **g** Schematic diagram of the in vivo protocol of BLM-induced pulmonary fibrosis induced by Nutlin 3b treatment or vehicle. Lung tissues were collected on day 18 after BLM injection. **h** Representative images of MT staining of the lungs of Nutlin 3b-treated mice. The bar indicates 100 μm. **i** Evaluation of fibrotic areas via MT staining. **j** Heatmap analysis of fibrosis markers, senescence and SASP among the control, BLM + vehicle, and BLM + Nutlin 3b groups. **k** Assessment of the expression of the fibrotic markers col3a1, col5a2, fibronectin, and tropoelastin in BLM-induced lung fibrosis after Nutlin 3b treatment via RT‒PCR analysis. **l** A schematic diagram of the mechanism by which a novel senolytic pathway targets aberrant PGAM1-Chk1 interactions. This image was created with BIORENDER

Previously, we reported that the Nutlin consensus motif resides at the amino terminus of PGAM1 and identified a *W68A* substitution in this motif as a genetic interference agent against its binding to Chk1 in vitro, although its enzymatic activity is conserved in this mutant.^22^ To assess the in vivo ability of the *W68A* mutation to inhibit PGAM-Chk1 binding, *Pgam1 W68A* knock-in mice were generated via CRISPR/Cas9 (Fig. 7a, b). The crossing of *Pgam1* ^*KI/+*^ mice generated *Pgam1* ^*KI/KI*^ progenies, according to Mendelian laws (Supplementary Fig. 16a). *Pgam1* ^*KI/KI*^ mice developed normally, and their physiological parameters were comparable to those of wild-type mice (Fig. 7c, and Supplementary Fig. 16b, c). First, we characterized the cytological properties of MEFs from *Pgam1* ^*KI/KI*^ mice. The proliferative capacity of these MEFs was comparable to that of wild-type MEFs, as both reached senescence similarly (Supplementary Fig. 16d, e). However, the immunoprecipitation assay revealed impaired PGAM1-Chk1 interaction, as expected, in the senescent *Pgam1* ^*KI/KI*^ MEFs (Supplementary Fig. 16f). In this context, the expression of senescence and SASP markers (p16^Ink4a^, Il6 and Cxcl1) was partially decreased in senescent *Pgam1* ^*KI/KI*^ MEFs (Supplementary Fig. 16g). Moreover, under DNA damage, the viability of senescent *Pgam1* ^*KI/KI*^ MEFs was lower than that of control MEFs (Supplementary Fig. 16h), suggesting that the genetic abrogation of PGAM1-Chk1 binding attenuated the survival capacity of senescent MEFs.

Next, we evaluated the therapeutic efficacy of interfering with the PGAM1‒Chk1 interaction in aging-related disorders, as several other senolytic approaches, e.g., ABT263 or D + Q, reportedly alleviate these disorders.^7^ For this purpose, we focused on lung fibrosis models for several reasons. First, human pulmonary fibrosis is an etiologically age-related disease, with an average age of onset of ~64–68 years.^44^ Second, mouse models of pulmonary fibrosis exhibit the accumulation of SnCs in their lesions, whose removal by senolysis is reported to restore the physiological capacity of the lungs.^45^ Third, a recent study identified FOXM1 as a crucial driver of the pathogenesis of pulmonary fibrosis.^46^ Bleomycin (BLM) was intratracheally instilled into the lungs of the mice to induce pulmonary fibrosis. Notably, during this protocol, the fibrotic area in the lung was significantly decreased in *Pgam1* ^*KI/KI*^ mice, as assessed by Masson’s trichrome staining (Fig. 7d, e). Moreover, several fibrosis markers (Col3a1, Col5a2, fibronectin and tropoelastin) were significantly downregulated in *Pgam1* ^*KI/KI*^ mice, which correlates with the observed histological findings (Fig. 7f).

Finally, we evaluated the therapeutic efficacy of Nutlin 3b in treating lung fibrosis. Nutlin 3b or vehicle was administered on experimental day 8 to investigate its biological effects on fibrosis progression (Fig. 7g).^47^ Nutlin 3b treatment significantly decreased the fibrotic area in the lung in a dose-dependent manner (Fig. 7h, i and Supplementary Fig. 17a, b) and downregulated several fibrotic markers (Col3a1, Col5a2, fibronectin and tropoelastin) and SASP (Fig. 7j, k), indicating that 20 mg/kg Nutlin 3b was most effective. We observed greater PGAM1-Chk1 interaction and FOXM1 protein accumulation in fibrotic lungs than in control lungs, both of which were suppressed by Nutlin 3b treatment (Supplementary Fig. 17c, d). In accordance with the histological data (Supplementary Fig. 18a), 20 mg/kg Nutlin 3b, but not Nutlin 3a, significantly downregulated the expression of fibrotic markers, positive SA-β GAL staining, and downstream targets of FOXM1 (Supplementary Fig. 18b–e). Consistently, thiostrepton, a FoxM1 inhibitor, exerted a mild senolytic effect on SnCs and significantly suppressed fibrotic areas and positive SA-β GAL staining in mouse lung fibrosis (Supplementary Fig. 19a–e). Interestingly, in the lung fibrosis model, the number of macrophage populations was largely reduced by Nutlin 3b (Supplementary Fig. 19f), similar to the findings in aged livers (Fig. 3d). We performed a coculture assay with macrophages and senescent fibroblasts derived from WT mice or *Pgam1* ^*KI/KI*^ mice (Supplementary Fig. 19g). Compared with young MEFs, macrophages cocultured with senescent WT MEFs displayed increased infiltration, while the PGAM *W68A* mutation in SnCs significantly impaired the activities of these macrophages (Supplementary Fig. 19h, i). Overall, both chemical and genetic interference with the PGAM1-Chk1 axis alleviated pulmonary fibrosis in vivo, which is an aging-related disease.

## Discussion

Our study revealed that aberrant glycolytic profiles resulting from increased PGAM1-Chk1 interaction occur in senescent cells, as in cancer cells.^23^ Posttranscriptional activation of HIF-2α supported enhanced glycolysis in SnCs, whose lactate byproduct also restored the PGAM1-Chk1 interaction. Strikingly, interference with PGAM1-Chk1 binding by Nutlin 3b essentially eliminated glycolytic SnCs but not nonsenescent controls (Fig. 7l). Nutlin 3b effectively alleviated in vivo functional declines in the aged liver, kidney, and lung, indicating the accumulation of FOXM1, a downstream module of HIF-2α involved in senescence. Consistently, chemical inhibition or genetic manipulation of PGAM1-Chk1 binding mitigated lung fibrosis, indicating that PGAM1-Chk1 interference is a promising senolytic for relevant disorders in aging.

The antiapoptotic properties of SnCs are regulated by several factors, including NF-κB,^48^ epigenetics,^19^ and the membranous apparatus.^49^ In addition, abnormal protein‒protein interactions (e.g., FOXO‒p53 binding)^50^ or contacts^51^ are also observed in SnCs, which we designate aberrant protein‒protein interactions in senescence (APIS). Here, we identified the enhanced PGAM1-Chk1 interaction as APIS. Several lines of evidence suggest that metabolic shifts in surviving SnCs can be attributed in part to adaptive responses due to DNA damage, exposure to proteotoxic stress caused by the SASP or dysregulated metabolism.^15,16^ Here, we elucidated the mechanism by which the PGAM1-Chk1 interaction enhances glycolysis during senescence. As enhanced glycolysis supports DNA integrity and repair,^26^ glycolytic inhibition by Nutlin 3b augments DNA damage in SnCs due to a decrease in PPP metabolites, which are essential substrates for the generation of nucleotide pools,^28^ and the suppression of the expression of DNA repair machinery, which is regulated by FOXM1. Interestingly, some senolytic drugs, e.g., ARV825 (an epigenetic modulator), also induce severe and lethal DNA damage in SnCs.^19^ These findings underscore the importance of enhanced glycolysis in senescence for the maintenance of DNA integrity, which is significantly impaired by Nutlin 3b.

Our findings strongly support the idea that the increase in PGAM1-Chk1 binding represents a potential pathological state of SnC accumulation. Similarly, Nutlin 3b, a PGAM1-Chk1 interference agent, improved several in vivo health parameters in aged mice, including chronic inflammation in tissues and biomarkers for frailty, similar to other senolytic drugs.^7^ In addition, Nutlin 3b partially decreased the blood levels of lactate, a byproduct of glycolysis, but not the fasting glucose levels in aged mice. Increased lactate is clinically viewed as a mortality indicator, mainly in patients with acute illness,^52^ who suffer from both hypoxic and pseudohypoxic conditions with activation of HIF-1α, a key factor in acute responses.^53^ Our study suggests that lactate is a mediator of senescence signaling. Lactate-GPR81 signaling is increased in SnCs, leading to the activation of RSK1 kinase to maintain PGAM1-Chk1 binding. While SASP factors include several senescence-associated metabolites, e.g., prostaglandins, lactate could be added to the list, which is supported by the recent concept that the establishment phase of senescence is affected by temporospatial factors and nonautonomous cellular mechanisms.^54^ Nutlin-3b could be effective as a senomorphic agent, partly because of the attenuation of lactate senescence signaling, and as a senolytic.

In addition to several resilience activities, responses to acute or intermittent hypoxia reportedly play protective roles in damaged or injured organs by restraining inflammation, promoting barrier functions, and enhancing tissue resilience,^55,56^ whereas chronic hypoxic stress exacerbates diseased and inflammatory states, e.g., increasing NF-κB.^55^ It has been controversial whether HIF-1α, a responder to acute hypoxia,^53^ mediates its positive or negative effect on aging,^57^ as either the ectopic expression or inactivation of HIF-1α extends the organismal lifespan of experimental models, such as *C*. elegans. Here, we demonstrated that senescence-induced stress activates the PGAM1-Chk1 interaction, resulting in the activation of HIF-2α (a homolog of HIF-1α) and increased glycolysis. Consistent with the notion that proteolytic responses are disorganized in SnCs, known as senescence-associated protein degradation (SAPD),^58^ senescence-inducing stress and lactate signaling stabilize HIF-2α to restore glycolytic features. Interestingly, recent studies suggest that HIF-2α plays opposing roles against HIF-1α in the pathogenesis of several diseases in the lung, kidney and other organs, as HIF-2α inactivation alleviates relevant symptoms and diseases.^59,60^ However, as *HIF-2α*^−/−^ mice display a progeroid-like phenotype,^61^ direct targeting of HIF-2α should be carefully designed, regarding with its timing, tissue context and relevant dysfunctions.

Another outcome of HIF-2α accumulation in SnCs is the activation of FOXM1. Among members of the FOX transcriptional family, FOXO proteins are strongly implicated in aging, although FOXM1, an antagonist of FOXOs, is reported to serve as a master driver of the cell cycle and cell proliferation.^42^ While constitutively FOXM1-expressing mice exhibit a cancer-prone phenotype,^40^ unbiased screening via genome-wide CRISPR-Cas9 identified FOXM1 as an apoptosis driver under the inhibition of Chk1,^62^ indicating its double-edged sword behavior in cancers. However, it is unclear why and how FOXM1 is required for the survival capacity of SnCs. Our findings indicate that FOXM1 dictates the ability of SnCs to survive by functioning in multiple ways, as a suppressor of the proapoptotic BH protein BIM, as an inducer of antiapoptotic Survivin, and as an enhancer of the DNA repair machinery. Thus, the combination of high glycolysis and FOXM1 activation restored the survival capacity of SnCs. As cellular fates for survival are largely based on the balance between proapoptotic and antiapoptotic factors, it is possible that the enhanced proapoptotic responses induced by Nutlin 3b might synergistically function with the suppression of antiapoptotic factors, e.g., ABT263 or dasatinib plus quercetin (D + Q), to induce senolysis. A recent study suggested that preserved endothelial functions are crucial for restoring the health of mice after senolytic treatment, e.g. D + Q.^63^ The distinct senolytic role of Nutlin 3b among several cell types, which is effective against senescent fibroblasts and macrophages, suggests its expected clinical potential, in addition to the results of long-term toxicity assessment of Nutlin 3b.

While senotherapy targets the molecular mechanisms by which aging per se reinforces the risk of developing aging-relevant diseases, its efficiency via chemical or genetic manipulation varies greatly among different tissues.^17^ Accordingly, the clinical application of senolysis should be carefully designed on the basis of the target profiles in aged organs. FOXM1 mRNA levels peak in the embryonic stage, followed by its gradual decline in many tissues tested until middle age, which is consistent with the notion that FOXM1 drives the cell cycle and proliferation.^42^ Nevertheless, in aged mice (90 wks), several organs (liver, lung, and kidney), but not others (WAT, heart and brain), presented increased FOXM1 expression compared with that in young or middle-aged mice. Interestingly, in vivo chronic stress caused by mild hypoxia enhances glycolysis to meet high energy demands, not in every organ but in distinct tissues, including the liver, lung, and kidney.^64^ It is possible that such organs vulnerable to energy adjustment under chronic stress are also sensitive to the accumulation of glycolytic SnCs in aged mice, which are exposed to chronic pseudohypoxic stress.^15^ Notably, the upregulation of FOXM1 in aged liver, lung and kidney tissues is efficiently suppressed by Nutlin 3b, which is consistent with the observations that APIS is a potential target for senotherapy.^50^ As senolysis alleviates aging-related dysfunctions in a tissue context-dependent manner,^17^ senolytic Nutlin 3b could be more effective for aged tissues that display increased activity of FOXM1. Dysregulated expression of FOXM1 is implicated not only in cancers but also in several aging-related diseases,^65^ e.g., pulmonary fibrosis, a disease with upregulated FOXM1,^46^ is mitigated by chemical or genetic inhibition of PGAM1-Chk1 binding. Collectively, the metabolic adaptation of SnCs into the glycolytic state is intriguingly coupled with their aberrant survival capacity and renders these SnCs susceptible to apoptotic stimuli caused by Nutlin 3b, an inhibitor of APIS.

Several limitations of the present study should be noted. First, the following “enzymatic” roles of glycolytic PGAM have been reported: tumorigenesis, immune response, stem cell or tissue repair, senescence and others.^10^ In contrast to the dynamic complexity of enzymatic PGAM, we revealed the impact of the “nonenzymatic” role of PGAM in senescence, as its enzymatic activity is not affected by Nutlin 3a or 3b, which interferes with PGAM-Chk1 binding. Thus, it remains to be clarified whether the modulation of “enzymatic” PGAM activity is effective for senotherapy. Second, recent studies reported that the periodic activation of transcriptional programs beginning in the very early life stage, including FOXM1 or Yamanaka factors, extends organismal lifespans.^66,67^ Thus, fine-tuning FOXM1 affects organismal health and aging. We observed the senolytic effect of the FoxM1 inhibitor thiostrepton on bleomycin-induced lung fibrosis in mice. However, the effects of FoxM1 inhibitors on aging or other aging-related diseases are still not fully understood. Third, the different therapeutic effects of two optical isomers, Nutlin 3a and 3b, are not exceptional and potentially important for clinical application, as reported previously.^68–71^ However, future clinical trials remain challenging, as our study on Nutlin 3b is limited to mouse models.

## Materials and methods

### Cell culture

Normal human fibroblasts IMR90 and MCR5 were obtained from the JCRB Cell Bank and ATCC, respectively. The PLAT-A packaging cell line was a kind gift from Dr. Toshio Kitamura (University of Tokyo). The non-small cell lung cancer cell line H1299 and the human vascular endothelial cell line EA-hy926 were obtained from ATCC. Human umbilical vein endothelial cells (HUVECs) were acquired from RIKEN BRC (RCB5489: HUE102). MCR5-ER:Ras, H1299, EA-hy926 and PLAT-A cells were cultured in Dulbecco’s modified Eagle’s medium (DMEM) supplemented with 10% fetal bovine serum (FBS). IMR90 cells were cultured in MEM supplemented with 10% FBS. HUVECs were cultured in complete medium (MCDB131 (Thermo Fisher), 20 mM HEPES buffer, 10 U/ml heparin, and 30 µg/ml ECGS (Corning)) supplemented with 10% FBS. All the cell lines used in this study were confirmed to be mycoplasma negative.

### Preparation of senescent cells (SnCs)

For preparation of oncogene-induced, senescent MRC5-ER:Ras cells, MCR5-ER: Ras cells were treated with 20 nM 4-hydroxytamoxifen (4-OHT) for 16 days. For preparation of oncogene-induced senescent IMR90 cells (OIS), empty or oncogenic H-Ras G12V was introduced into IMR90 cells with a retroviral vector (pBabepuro). After retroviral infection, cells were selected with 1 μg/mL puromycin. For preparation of DNA damage-induced senescence, IMR90 cells and human vascular endothelial cells (EA.hy926 and HUVEC) were treated with 100 μM Etoposide for 48 h, and then media were changed and cells were cultured under non-stressful conditions. Senescence induction was confirmed with SA-β-gal staining or decreased expression of Lamin B1 protein. IMR90 Cells were irradiated at dose of 10 Gy by Cs-137 Gammacell 40 Exactor (Best Theratronics Ltd., Canada) for senescence-induction. IMR90 cells were continuously cultured until they reach replicative senescence, as reported previously.^72^ Premature Senescence was provoked by inactivation of PGAM1 or Chk1, as previously reported.^22,73^ For collection of primary mice macrophage cells, three mL of 4% thioglycollate (BD) was injected intraperitoneally into 12-weeks old male C57BL/6 mice. Four days later, mice were euthanized and peritoneal macrophages (PMs) were harvested by lavaging the peritoneal cavity with 10 mL of ice-cold PBS. Isolated PMs were counted, resuspended in fresh medium, and used for subsequent experiments. For the culture of PMs, PMs were seeded at one million cells/mL in RPMI medium (Nakalai) supplied with 10% FBS, 1% of penicillin and gentamicin (Nakalai). Culture medium was changed every 2 days until they reached replicative senescence.

### Transfection and retroviral infection

Lipofectamine siRNA MAX (Invitrogen, USA) was utilized for siRNA transfection. siRNAs used in this study are presented in Supplementary Table 3. Transfection of vectors was performed with Lipofectamine2000 (Invitrogen). Retrovirus was produced using the PLAT-A packaging cell line.^74^ IMR90 cells were co-incubated with retrovirus for infection. Infected IMR90 cells were treated with hygromycin (75 µg/mL), or puromycin (1 µg/mL) for drug selection of infected cells.

### Luciferase reporter assay

Luciferase reporter assays were performed using a Nano-Glo Dual-Luciferase Reporter assay system (Promega K. K.). NanoLuc plasmids (pNL1.2) containing the 5’ flanking region of the BIM sequence and pGL4.54[luc2/TK] plasmids, as an internal control, were co-transfected in SnCs. The luciferase signal was detected using a GloMAX Explorer (Promega K.K.). Luminescence of the NanoLuc signal was normalized using the Luc2 signal.

### Immunoblotting and immunoprecipitation

Protein lysates were prepared as previously described.^75^ Briefly, mouse tissue (liver and lung) or IMR90 cells were lysed in buffer containing 50 mM Tris–HCl (pH 7.5), 200 mM NaCl, 1 mM ethylenediaminetetraacetic acid (EDTA), 10% glycerol, 0.5% Triton-X100, 50 mM NaF, 1 mM dithiothreitol (DTT), 1 mM Na3VO4, 1 mM phenylmethanesulfonyl fluoride (PMSF), and a protease inhibitor cocktail (Sigma–Aldrich). BioMasher II (Nippi, Japan) was used to dissolve mouse tissue in buffer. Protein lysates were boiled in Laemmli sample buffer for denaturation. Equivalent amounts of protein were resolved by SDS-PAGE. Anti-human Chk1 (C9358, 1:3000) and anti-Actin (A4700, 1:1000) were purchased from Sigma–Aldrich. Anti-human HIF-2α (ab199, 1:500) anti-Lamin B1 (ab16048, 1:1000), anti-p21CIP1 (ab7960, 1:500), anti-Aldolase (ab169544, 1:1000), anti-PGK1 (ab38007, 1:1000), anti-ENO1 (ab155102, 1: 1000), anti-PGAM1 (ab129191, 1:1000), anti-mouse Chk1 (ab32531,1:1000) and anti-phospho-Chk1 (Ser280; ab59988, 1:1000) were from Abcam (Cambridge, UK). Anti-c-myc (9E10, 1: 200) and anti-p53 (DO-1, 1:1000) were from Santa Cruz Biotechnology (Dallas, TX). Anti-phosphor p53 (Ser15, #9284, 1:1000), anti-E2F1 (#3742, 1:1000), anti-FOXM1 (#20459, 1:500), anti-BIM (#2933, 1:500), anti-Cleaved Caspase 3 (#9664, 1:500), anti-RSK1 (#9355, 1:10000) and anti-phospho-RSK1 (Thr573, #9346, 1:1000), anti-IRF3 (#11904T,1:1000), anti-phospho-IRF3(Ser396, #4947T, 1:1000), anti-TBK1 (#3013S, 1:1000), anti-phospho-TBK1(Ser172, #5483S, 1:1000) were purchased from Cell Signaling Technology (Danvers, MA). Anti-GAPDH (MAB374, 1:3000) came from Merck Milipore (USA). Anti-p16Ink4a (No.11104, 1:500) was from Immuno-Biological Laboratories Co., Ltd (Japan). Anti-HIF-1α (NB100-105, 1:200) and anti-mouse HIF-2α (NB100-122, 1:200) were supplied by Novus Biologicals (CO, USA). Anti-mouse Chk1 (25887-1-AP, 1:1000) and anti-mouse FOXM1 (13147-1-AP, 1:1000) were purchased from Proteintech (USA). Anti-β-tublin (No.010-25043, 1:1000) was purchased from Wako (Japan). Anti-phospho-HIF-2α (Ser12) was generated in this study. Phosphopeptides (Cys-KKRSS(pS)ERRKE-OH) were subcutaneously injected with adjuvant into rabbits. After the fifth sensitization, whole blood was collected and phospho-antibodies were purified by affinity chromatography.

For immunoprecipitation assays, cell lysates were precipitated by the relevant antibody conjugated with protein G-agarose beads for 2 h. Beads were washed four times with lysis buffer, and boiled in 2x Laemmli sample buffer. Denatured immune complexes were resolved by SDS-PAGE. For detection of ubiquitinated HIF-2α proteins, a His-ubiquitin assay was performed as described previously.^75^

### SA-β-Gal staining of cultured cells and in vivo

SA-β-Gal staining was performed as previously described.^76^ Briefly, senescent or control cells were washed twice with ice-cold phosphate-buffered saline (PBS) and fixed with 2% formaldehyde and 0.2% glutaraldehyde in PBS buffer for 5 min at room temperature. Fixed cells were washed twice with PBS buffer, followed by incubation with SA-β-Gal staining solution (40 mM citric acid/Na phosphate buffer, 5 mM K_4_[Fe(CN)_6_]3H_2_O, 5 mM K_3_[Fe(CN)_6_], 150 mM NaCl, 2 mM MgCl_2_, 1 mg/mL X-gal, pH6.0) overnight at 37 °C.

SA-β-gal staining of lung tissue was performed as previously described.^77^ Tissue samples of mice lung were freshly harvested and embedded in OCT compound, then frozen at –80 °C. Sections were air-dried for 20 min and then fixed with 2% formaldehyde and 0.2% glutaraldehyde for 5 min at room temperature. After fixation, the sections were washed twice with PBS and then incubated with SA-β-gal staining solution at 37 °C overnight. Following incubation, the stained sections were imaged and mounted for further analysis. SA-β-gal staining of liver tissue was performed as previously described.^78^ Briefly, liver tissue was collected and embedded in OCT compound to prepare frozen blocks, and frozen sections were prepared. Sections were then cut at a thickness of 20 μm and fixed in 0.2% glutaraldehyde for 5 min. Frozen sections were stained by SA-β-gal staining solution at pH 4.0 for 2 h at 37 °C. The percentage of SA-β-gal–positive area was quantified using ImageJ (Fiji).

### RNA analysis

Total RNA was extracted with TRIzol (Invitrogen). For preparation of a cDNA pool, mRNA was reverse-transcribed using a ReverTra Ace qPCR RT kit (Toyobo, Japan). Real-time quantitative PCR was performed using Thunderbird SYBR qPCR mix (Toyobo). Amplification of DNA was detected with a Thermal Cycler Dice Real-Time system III (Takara Bio., Japan). Gene expression was normalized to *Rpl13a* mRNA levels and presented as values relative to controls. Primers used are shown in Supplementary Table 4.

### Detection of protein‒protein interactions in living cells

LgBiT-PGAM1- and Chk1-SmBiT-expressing vectors were introduced into MCR5-RasER, IMR90 or H1299 cells. NanoBiT signals were detected with a Nano-Glo Live Cell assay kit (Promega K.K.). The signal intensity was normalized to the cellular ATP content via a CellTiter-Glo kit (Promega K.K.).

### Measurement of glycolytic parameters

Glycolytic flux was measured as previously described.^23^ IMR90 cells were cultured in fresh MEM containing D-[3-^3^H] glucose. At 0 and 6 h, aliquots of medium were collected and deproteinated with perchloric acid. The supernatant was applied to a column filled with DOWEX 1×8 200–400 MESH Cl resin (Sigma–Aldrich). The amount of [^3^H] in the flow-through was measured with a liquid scintillation counter (LSC-6100, Hitachi Aloka Medical Ltd., Japan) as the metabolized glucose, and those counts were normalized to the protein content. Radioisotope experiments were performed at the Radioisotope Research Center of the Agency for Health, Safety & Environment, Kyoto University. The glucose and lactate concentrations in the culture media were determined via a glucose or lactate assay kit (BioVision), respectively. Glucose consumption or lactate production was normalized to the protein content of the corresponding cell lysate. 2NDBG was used for visualization of glucose uptake. Senescent or nonsenescent IMR90 cells were cultured in glass-bottom dishes (Matsunami, Japan). The cells were incubated in MEM containing 500 µM 2NDBG for 2 min. The glass-bottom dishes were washed twice with PBS, and the medium was then replaced with fresh MEM for observation.

### Microarray

For preparation of total RNA from culture cells, senescent IMR90 cells were prepared by ectopic expression of Ras G12V. After treatment with 40 µM Nutlin 3b or DMSO for 48 h, cells were collected for RNA isolation. For preparation of total RNA from mice tissues, liver, kidney, lung and WAT samples from 10 or 90 weeks-old mice were homogenized by bead crusher μT12 (Taitec Co., Ltd). Total RNA was prepared with an RNeasy kit (Qiagen, Germany). Using a Quick Amp Labeling Kit (Agilent, Santa Clara, CA), labeled cRNA was generated from total RNA. Microarray assays were performed with a SurePrint G3 Human Gene Expression 8×60k v3 microarray kit (Agilent, SUSA).

Enrichment Analysis was performed as described in the ChIP Atlas information (https://chip-atlas.org/).^79^ Among 29,451 ChIP datasets in NCBI, EBI and DDBJ, 25,112 with a threshold of significance (>500) were employed. In microarray analysis of senescent cells after Nutlin 3b treatment, 1168 genes were downregulated over 2-fold, compared to controls. Fold Enrichment in overlapping between ChIP datasets and Nutlin 3b targets are listed. Gene Set Enrichment Analysis (GSEA) was performed with software ver. 4.2.3. The microarray dataset was analyzed for deflection of the target genes of FOX family, obtained from the ChIP Atlas.

### Metabolomics analysis

For IMR90 cells, the medium was removed from culture dishes. A methanol solution was added and stirred. Milli-Q water containing 10 μM of the internal standard was added and centrifuged (2300 × *g*, 4 °C, 5 min). The extract was transferred to an ultrafiltration tube (Ultra Free MC PLHCC, HMT, Centrifugal Filter Unit 5 kDa) and centrifuged (9100 × *g*, 4 °C, 120 min). Cationic metabolites were measured with Agilent CE-TOFMS system (Agilent Technologies). Anionic metabolites were measured with Agilent CE system and Agilent 6460 TripleQuad LC/MS. The CE-TOFMS data were extracted automatically using MasterHands ver. 2.19. 0.2 (Keio University) For CE-QqQMS measurement data, automatic peak extraction was performed using MassHunter (MassHunter Quantitative Analysis B. 6.00 Agilent Technologies, Santa Clara, USA). In each software, the mass to charge ratio (m/z), migration time (migration time: MT) and peak area value were obtained as peak information. The obtained peak area values were converted to relative area values using the following equation. Relative area value = (Area value of the target peak/Area value of internal standard) × (1/protein content).

The detected peaks were checked and searched against substances registered in the HMT metabolite library based on the values of m/z and MT. The relative area values were converted to absolute quantification values using standards. For quantification, the peak area values corrected by internal standards were used, and the concentration was calculated by preparing a calibration curve consisting of three points for each metabolite.

### Mouse experiments

All the animal experiments were approved by the Animal Care and Use Committee of Kyoto University Graduate School of Medicine (ethical approval no. “Medkyo 25266” and approval for recombinant DNA experiment no. “220102”) and conducted in accordance with the institutional guidelines of Kyoto University and the national Guidelines for the Care and Use of Laboratory Animals published by the Ministry of Education, Culture, Sports, Science and Technology of Japan. All efforts were made to minimize animal suffering in accordance with Japanese government regulations, which comply with the International Guiding Principles for Biomedical Research Involving Animals by CIOMS-ICLAS. C57BL/6 mice were purchased from The Jackson Laboratory (Bar Harbor, ME, USA). For the intraperitoneal injection of Nutlin 3a or 3b into mice, Nutlin 3a or 3b was dissolved in a 1:1 mixture of PBS and polyethylane glycol 400 (PEG400). Twenty-month-old mice were intraperitoneally injected with 11.62 mg/kg Nutlin 3b every week for three months. One week after the 3-month treatment, the wire-hang test was performed. The mice were placed on a wire grid, and the grid was inverted. The grid was held 35 cm above the cage floor, and the holding time was measured. The wire-hang test was performed 3 times, and the average number of times was used for data analysis. After the wire-hang test, the mice were sacrificed for analysis.

### Generation of *Pagm1 W68A* knock-in mice

The W68A mutation of *Pgam1* gene was inserted into C57BL/6 mice using the CRISPR/Cas9 system. To achieve this, tracrRNA (5’-AGC AUA GCA AGU UAA AAU AAG GCU AGU CCG UUA UCA ACU UGA AAA AGU GGC ACC GAG UCG GUG CUU U-3’), ssODN (5’-AGA GAG CAA TCC GGA CCC TGG CGA CAG TCC TGG ATG CCA TTG A-3) and Streptococcus pyogenes Cas9 protein were transferred into fertilized eggs derived from C57BL/6 mice by electroporation. The genomic sequence in the *Pgam1* gene locus in F0 generation mice was analyzed by DNA sequencing to confirm that the tryptophan (TGG) at 68 to alanine (GCG) was substituted. To eliminate potential off-target mutations induced by the CRISPR/Cas9 system, the generated mice were backcrossed with C57BL/6 mice for at least 10 generations.

Genotyping was carried out according to the genomic PCR protocol. Specific primer sets were designed to differentiate between wild-type and *Pgam1 W68A* knock-in alleles. Primer sequences were as follows; for wild-type Fw 5’- CAC TGT GTG GGT TCT GGG G-3’, Re 5’-GGC ATC CAG GAC TGT CCA G -3’, and for W68A Fw 5’-CAC TGT GTG GGT TCT GGG G -3’, Re 5’-GGC ATC CAG GAC TGT CGC C -3’.

Mouse embryonic fibroblasts (MEFs) were prepared from 13.5-day embryos of wild-type or *Pgam1* ^*KI/KI*^ mice as described previously.^75^ After removing the head and blood organs, the embryo was placed in a plastic plate. Embryo was chopped up by surgical knife and dispersed in 0.1% trypsin. MEFs were cultured for two passages at 37 degree, and then utilized in the experiment.

### Bleomycin (BLM)-induced pulmonary fibrosis

In the bleomycin (BLM)-induced pulmonary fibrosis model, 11- to 12-weeks-old CD1 female mice were randomly assigned to either group, BLM or vehicle. BLM in PBS or PBS alone was administered intratracheally on day 0. In senolysis experiment, the drug, Nutlin 3b or 3a, was injected intraperitoneally four times per week starting on day 8 after BLM induction. Thiostrepton, a FoxM1 inhibitor, was dissolved in 20% N,N-dimethylacetamide, 75% PEG400 and 5% Tween 80, as described previously.^80^ Mice are intraperitoneally injected with thiostrepton at 20 mg/kg every other day since day 8 after BLM induction. On day 18, all mice were sacrificed, whose lungs were collected for pathological and RT-PCR analyses. Twenty weeks-old *Pgam1* ^*KI/KI*^ mice (C57BL6) were utilized for BLM-induced pulmonary fibrosis model.

### Immunohistochemical staining

For pathological analysis, a piece of liver, kidney and lung was fixed with 4% PFA for 2 days and then embedded in paraffin. Tissue sections were stained with hematoxylin-eosin staining. p21 in liver and lung were stained with anti-p21(Abcam, ab188224) and F4/80 in liver were stained with anti-F4/80 antibody (eBioscience, 14-4801) using ABC methods.^81^ F4/80, B220 and CD3 in lung were stained with anti-F4/80, anti-B220 (BD Pharmingen, 557390), and anti-CD3 antibody (Cell Signaling Technology, 99940). Colonies of immune cells with over 30 cell counts were evaluated.

### Statistical analysis

All data are expressed as the mean ± standard error of the mean (SEM). Comparisons between two independent groups were analyzed using unpaired Student’s two-tailed *t* tests. Comparisons between multiple groups were analyzed using one-way analysis of variance (ANOVA) and Dunnett’s multiple comparison test. The required sample size was determined in advance using a sample size calculation for comparing two means.^82^ Statistical analysis was performed using BellCurve (Social Survey Research Information Co., Ltd. Japan) or Excel.

## Supplementary information

Supplementary materials (Clean)

## Data Availability

The data generated in this study are available from the corresponding author upon reasonable request. The microarray data generated in this study have been deposited in the NCBI Gene Expression Omnibus repository under accession codes GSE217601 and GSE247440. The *Pgam1 W68A* mutant knock-in mice described in the paper are patent-pending (WO2024/135719), and a separate agreement may be required for provision.
