# Supplementary material for: Abrogation of aberrant glycolytic interactions eliminates senescent cells and alleviates aging-related dysfunctions
Source: Signal Transduct Target Ther. 2025 Dec 15;10:402. doi: 10.1038/s41392-025-02502-6 (PMC12702999; doi:10.1038/s41392-025-02502-6)
Supplement: Supplementary file 1 — Supplementary materials (Clean) [file 41392_2025_2502_MOESM1_ESM.docx]

Supplementary Materials for

Abrogation of aberrant glycolytic interactions eliminates senescent cells and alleviates aging-related dysfunctions

Takumi Mikawa, Masahiro Kameda, Sumiko Ikari, Eri Shibata, Shuyu Liu, Sawa Miyagawa, Koh Ono, Tomiko Ito, Akihiko Yoshizawa, Masataka Sugimoto, Shuichi Shibuya, Takahiko Shimizu, Julio Almunia, Noboru Ogiso, Gwladys Revêchon,　Alberta Palazzo, David Bernard, Hiroaki Kanda, Tomoyoshi Soga, Keiyo Takubo, Shin Morioka, Junko Sasaki, Takehiko Sasaki, Akihiro Itamoto, Takayuki Fujii, Hiroshi Seno, Nobuya Inagaki, and Hiroshi Kondoh

Correspondence to: hkondoh@kuhp.kyoto-u.ac.jp

**This PDF file includes:**

Materials and Methods

Supplementary Figures. 1 to 19

Supplementary Tables 1 to 4

Raw data of Western blots

Materials and Methods

**Reagents**

Nutlin 3b and ABT-263 were obtained from Cayman Chemical (USA). Nutlin 3a, antimycin A, Rotenone, L-laclate, etoposide and 4-hydroxy tamoxifen (4-OHT) were purchased from Sigma–Aldrich (USA). MG132 was purchased from Peptide Institute, Inc. (Japan). 2-deoxy glucose was purchased from Nacalai Tesque (Japan). 2-deoxy-2-[(7-nitro-2,1,3-benzoxadiazol-4-yl) amino]-D-glucose (2NBDG) was purchased from DOJINDO Laboratories (Japan). BI D1870, Q-VD-Oph and necrostatin were obtained from Selleck Chemicals (USA). ADZ0095 and BPTES as inhibitor for MCT4 and GLS1 were obtained from Selleck Chemicals and Sigma–Aldrich, respectively. Polyinosinic:polycytidylic acid (Poly I:C) was purchased from Sigma–Aldrich. Thiostrepton were obtained from Cayman Chemical Company (USA). Recombinant human IL6, IL1b, and TNFα was purchased from Proteintech Group Inc, (USA). Recombinant CCL2, CXCL12, and OPG were purchased from PeproTech (USA). Recombinant IGFBP 1, 2 and 5 were purchased from Abcam. Recombinant IGFBP3, 4, 6 and AREG were purchased from R&D systems (USA).

**Plasmid DNA**

NanoBiT vectors were generated using the Flexi Vector System (Promega K.K., Japan). In brief, human *PGAM1* or *Chk1* cDNAs were subcloned into pF4A plasmids. Subsequently, the PGAM1 or Chk1 gene was subcloned into N-terminal LgBiT-tagging plasmids (pFN33K) or C-terminal SmBiT-tagging plasmids (pFN36K), respectively. Finally, LgBiT-PGAM1 and Chk1-SmBiT were subcloned into pHygro MarxIV vectors. Mutated versions of Chk1 (S280A, S280D) were generated by PCR-based mutagenesis. pLNCX2 ER:ras was a gift from Masashi Narita (Addgene plasmid # 67844 ; http://n2t.net/addgene:67844 ; RRID:Addgene_67844) ^S1^. HA-HIF2α WT-pBabe-Puro and HA-HIF2α-pcDNA3 was a gift from William Kaelin (Addgene plasmid #18950; http://n2t.net/addgene:18950; RRID:Addgene_18950, Addgene plasmid #26055; http://n2t.net/addgene:26055; RRID:Addgene_26055) ^S2^. HA-HIF2α (S12A and S19A) mutants were generated by PCR-based mutagenesis. The pBabe-puro-Ras G12V plasmid was a gift from Dr. Kayoko Maehara (Kio University, Nara, Japan). pcDNA3-HA-p53, CMV-HA-Mdm2 and CMV-His-Ub vectors have been described previously ^75^. For luciferase reporter assay, several 5’ flanking region of the BIM sequence, distal1 (-5197 ~-4397 bp), distal2 (-2563~-1560 bp) and proximal one (-925~-1bp) or a series of deletion mutant of proximal region were generated by artificial gene synthesis. These fragments were subcloned into in NanoLuc plasmids (pNL1.2).

**Co-culture assay between macrophages and senescent fibroblasts**

For co-culture assay, transwell cell migration plate with 8um pore (Corning) was used. Replicative senescent or non-senescent MEFs at 0.1 million cells / mL were seeded on the bottom of the lower well, while 1 million cells / mL of isolated peritoneal macrophages (PMs) or Raw264.7 cells were seeded in the upper chamber. Cells were cultured in DMEM for 20 hours and macrophages migrated through the membrane were stained with Crystal violet (Sigma). Positive staining cells were counted under a microscope. Raw264.7 cells were obtained from ECACC (91062702）.

**Chromatin immunoprecipitation (ChIP)-qPCR assay**

Chromatin immunoprecipitation (ChIP) was performed using the SimpleChIP Enzymatic Chromatin IP Kit (Cell Signaling Technology, #9003) according to the manufacturer’s instructions. Briefly, RasG12V-induced senescent or non-senescent IMR90 cells were fixed by 1% formaldehyde for 10 min. After fixation, cells were harvested using a cell scraper and processed to isolate nuclei following the manufacturer’s protocol. The isolated nuclei were treated with micrococcal nuclease to fragment the chromatin, followed by sonication to disrupt the nuclear membrane. The resulting chromatin was immunoprecipitated using 5μg anti-HIF2 antibody (NOVUS, NB100-122) or normal rabbit IgG (CST, #2729P). DNA was purified and analyzed by quantitative PCR. Primers targeting the FOXM1 promoter region were designed based on a previous report ^S3^. FOXM1 Promoter 1; fwd 5’- AGC AGA CGA TCG TTC ACT GT -3’, Rev 5’- TCC CCT TTT CAA AGC TCG GC -3’. FOXM1 Promoter 2; fwd 5’- GGC CCT TGG TCA GGG AAT AG -3’, Rev 5’- TTG ATG GTG GGT TGG ATG GG-3’. Actin promoter; fwd 5’- AGC GCG GCT ACA GCT TCA -3’, Rev 5’- CGT AGC ACA GCT TCT CCT TAA TGT-3’.

**LC-MS/MS Analysis of Nutlin 3b**

For preparation of plasma samples, mouse plasma (10 µL) was mixed with 990 µL of methanol, vortexed, and centrifuged at 10,000 × g for 10 min at 4 °C. An aliquot of 100 µL of the supernatant was transferred to a glass tube and spiked with 10 pmol of ketoconazole as an internal standard. Subsequently, 250 µL of methanol and 800 µL of chloroform were added, followed by vortexing. After the addition of 400 µL of ultrapure water and 50 µL of 1 M sodium chloride, the mixture was vortexed for 2 min and centrifuged at 1,200 × g for 4 min at room temperature. The lower organic phase was collected, evaporated under a nitrogen stream, and reconstituted in 75 µL of methanol and 25 µL of ultrapure water. A 10 µL aliquot was injected into the LC-MS/MS system. For preparation of tissue samples (liver and brain), tissue samples (10 mg) were homogenized in 100 µL of chloroform/methanol (1:1, v/v) using a BioMasher II homogenizer (Nippi, Japan). The homogenate was mixed with 900 µL of chloroform/methanol (1:1, v/v), vortexed, and centrifuged at 10,000 × g for 10 min at 4 °C. A 100 µL aliquot of the supernatant was transferred to a 1.5 mL tube and diluted with 900 µL of methanol. From this, 100 µL was transferred to a glass tube, spiked with 10 pmol of ketoconazole, and processed identically to plasma samples. Ketoconazole was purchased from Selleck Biotechnology (Japan). Methanol, chloroform, ultrapure water, acetonitrile, 1 M sodium chloride solution, and 1 M ammonium acetate solution were obtained from Fujifilm Wako Pure Chemical (Japan).

Quantification of Nutlin 3b was performed using reverse-phase liquid chromatography coupled to tandem mass spectrometry (LC-MS/MS), based on a previously published method ^S4^ with minor modifications. The LC-MS/MS system consisted of a QTRAP 5500 mass spectrometer (AB SCIEX, USA) interfaced with a Nexera X2 HPLC system (Shimadzu, Japan) and an HTC PAL autosampler (CTC Analytics, Switzerland). Chromatographic separation was performed using a COSMOCORE 2.6C18 column (2.1 × 150 mm, 2.6 µm; Nacalai Tesque, Japan), maintained at 60 °C. The mobile phase consisted of: Mobile phase A: isopropanol/acetonitrile/1 M ammonium acetate (160:40:1, v/v/v), Mobile phase B: acetonitrile/ultrapure water/1 M ammonium acetate (160:40:1, v/v/v). The gradient program was as follows: 0–1 min: 30% A / 70% B, 1–3 min: linear gradient to 90% A / 10% B, 3–7.5 min: held at 90% A / 10% B, 7.5–12 min: re-equilibrated to 30% A / 70% B. Detection was carried out in positive ion mode using multiple reaction monitoring (MRM). The MRM transitions were: Nutlin 3b: m/z 581.164 → 99.064 (collision energy: 85 V), Ketoconazole (internal standard): m/z 531.149 → 82.053 (collision energy: 89 V).

The drug half-life was calculated using the exponential decay model, C(t)=C_0_ e^−^*^k^*^t^ , where *C_0_* is the initial concentration, *k* is the elimination rate constant, and *t* is time.

**Toxicity assessment in mice**

For short-term toxicity assessment of drugs, young C57BL/6 mice at 10 weeks old were intraperitoneally injected once at 50, 100, or 200mg/kg of Nutlin 3a or 3b. After the injection of drugs at 100mg or 200mg/kg, the survival, body weights, and blood parameters of mice were evaluated within 24 hours. After one-shot injection of Nutlin 3b at 50 or 100mg/kg into mice, seven days follow-up were performed for measurement of body weights and blood parameters. Blood parameters were analysed by Fuji DRI-CHEM NX700i (Fuji Film, Japan). For long-term toxicity assessment, Nutlin 3b at 11.62 mg/kg was intraperitoneally injected into young C57BL/6 mice at 10 weeks old every week for three months. Each group comprises six mice (female n=3 and male n=3). Complete blood counts were obtained by collecting blood from the retro-orbital sinus of mice using EDTA-2K–treated capillary tubes, and measurements were performed with a PCE-310 hematology analyzer. Blood parameters test, histopathological and RT-PCR analysis of tissues were performed at the end of the study.

**ELISA assay**

The SASP concentration of the culture medium was measured using an AuthentiKine IL6 or IL1 ELISA assay kit (Proteintech Group Inc). In brief, appropriately diluted medium was added to plates precoated with capture antibodies. Then biotin-labeled IL6 or IL1b antibodies were reacted to target proteins, and streptavidin-HRP was attached to biotin. TMB was used as the HRP substrate for colorimetric determination.

[**Immunofluorescent staining**](https://lsd-project.jp/weblsd/c/begin/immunofluorescent%20staining)

Immunofluorescent staining of γH2AX was described previously ^75^. Briefly, IMR90 cells were treated with 40 μM Nutlin 3a or Nutlin 3b for 72 h. Pre-permeabilised cells were fixed with 3.7% paraformaldehyde. After permeabilization of cell membranes, cells were treated with blocking buffer (0.1% skim milk, 0.1% BSA in PBS) and primary antibody (anti-γ-H2AX phospho-Ser139 antibody). Subsequently, cells were treated with secondary antibody solution (Alexa Fluor 555). Nuclei were stained with DAPI. Images were recorded using a fluorescence microscope (IX-73, Olympus, Japan).


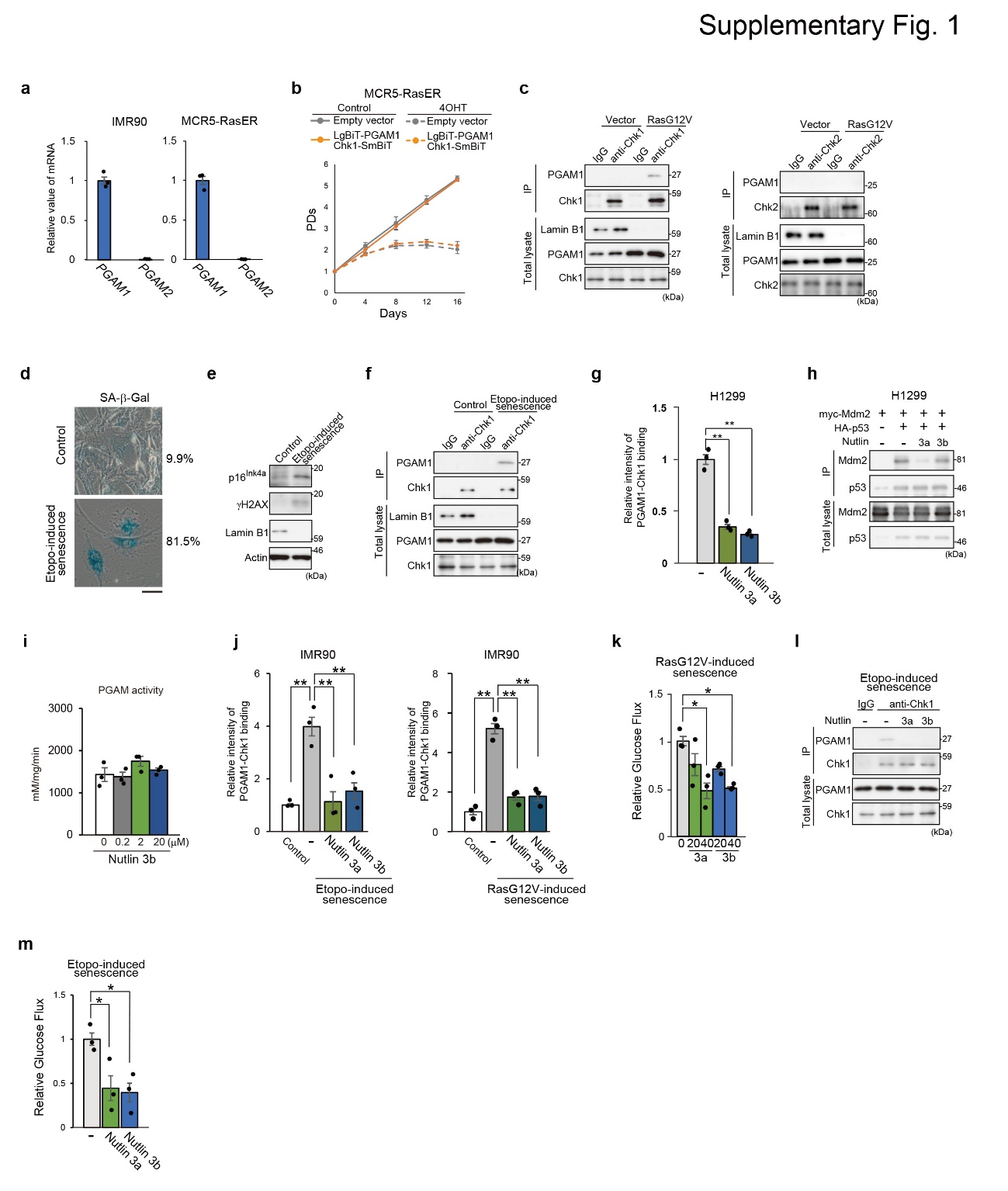


**Supplementary Figure 1. PGAM1-Chk1 interaction in senescent human primary fibroblasts and lung cancer cells (Relevant to Figure 1).**

**a.** Expression profiles for PGAM1 and 2 in human fibroblasts, IMR90 (left panel) and MCR5-RasER (right). Relative values for indicated mRNAs evaluated by RT-PCR are shown after normalization against *RPL13a* mRNA levels (n=3, biological replicates). **b.** Growth curves of MCR5-RasER cells with or without LgBiT-tagged PGAM1 and SmBiT-tagged Chk1. With or without tamoxifen treatment, proliferative potentials are shown as numbers of population doublings (n=3, biological replicates). **c.** Immunoprecipitation assay for endogenous PGAM1 and Chk1 or Chk2 proteins (left and right panels, respectively) in Ras G12V-expressing senescent cells (SnCs). Senescent and non-senescent cells were prepared by retroviral infection with Ras G12V expression vectors or empty vectors, respectively. Lysates of collected cells were immunoprecipitated with anti-Chk1 or anti-Chk2 antibody, followed by western blotting using anti-PGAM1 antibody. (**d.-f.**) IMR90 cells, human primary fibroblasts, were exposed to DNA-damaging etoposide (100 μM) for 2 days, followed by stress-induced senescence. Senescent features were evaluated. Percentages of SA-β GAL staining (**d.**) and protein levels of p16^Ink4a^, Lamin B1, and γH2AX (**e.**) were shown. Bar indicates 100 μm. **f.** Immunoprecipitation assay for endogenous PGAM1 and Chk1 proteins in etoposide-induced SnCs. Senescent and control cells were collected after 6h treatment with MG132. Lysates were immunoprecipitated with anti-Chk1 antibody. (**g. and h.**) Effects of Nutlin 3a and 3b in H1299 lung cancer cell lines. **g.** NanoBiT assay to detect PGAM1-Chk1 binding in cancer cells. LgBiT-PGAM1 and Chk1-SmBiT were retrovirally introduced into H1299 lung cancer cells. After 48 h treatment with Nutlin 3a or 3b, cells were collected for NanoBiT assay (n=3, biological replicates). **h.** Effect of Nutlin 3a and 3b on p53-Mdm2 interaction. Immunoprecipitation was performed to detect p53-Mdm2 interaction in H1299 cancer cells. HA-p53 and myc-Mdm2 were introduced into H1299 cells. After 48 h of treatment with Nutlin 3a or 3b, cells were collected for immunoprecipitation assay. **i.** PGAM enzymatic activity in the presence of Nutlin 3b. Recommbinant PGAM1 proteins were applied for its enzymatic assay in the presence of Nutlin 3b at the indicated dosages. **j.** NanoBiT assay between PGAM and Chk1 in SnCs during Nutlin-3a and -3b treatment. IMR90 cells expressing PGAM-Chk1-NanoBiT were prepared, which were treated with etoposide (left panel) or with oncogenic stress (right) to induce premature senescence. PGAM-Chk1-NanoBiT was evaluated in these SnCs after treatment with Nutlin-3a or -3b. **k.** Glucose flux was measured in oncogene-induced senescent IMR90 cells with or without Nutlin 3a or 3b treatment (n=3, biological replicates). (**l. and m.**) Effects of Nutlin 3a and 3b in Etoposide-induced senescent IMR90 cells. **l.** Effects of Nutlin 3a and 3b on PGAM1-Chk1 interaction in Etoposide-induced senescence. Stress-induced senescent IMR90 cells were treated with Nutlin 3a or 3b for 48 h. Interactions between endogenous PGAM1 and Chk1 proteins were evaluated by immunoprecipitation assay. **m**. Glucose flux was measured in Etoposide-induced SnCs with or without Nutlin 3a or 3b treatment (n=3, biological replicates). Data represent the mean ± SEM. Single (*) and double (**) asterisks indicate statistical significance of p<0.05 and p<0.01, respectively. Statistical analyses were determined by one-way analysis of variance (ANOVA) and Dunnett’s multiple comparison test.

**
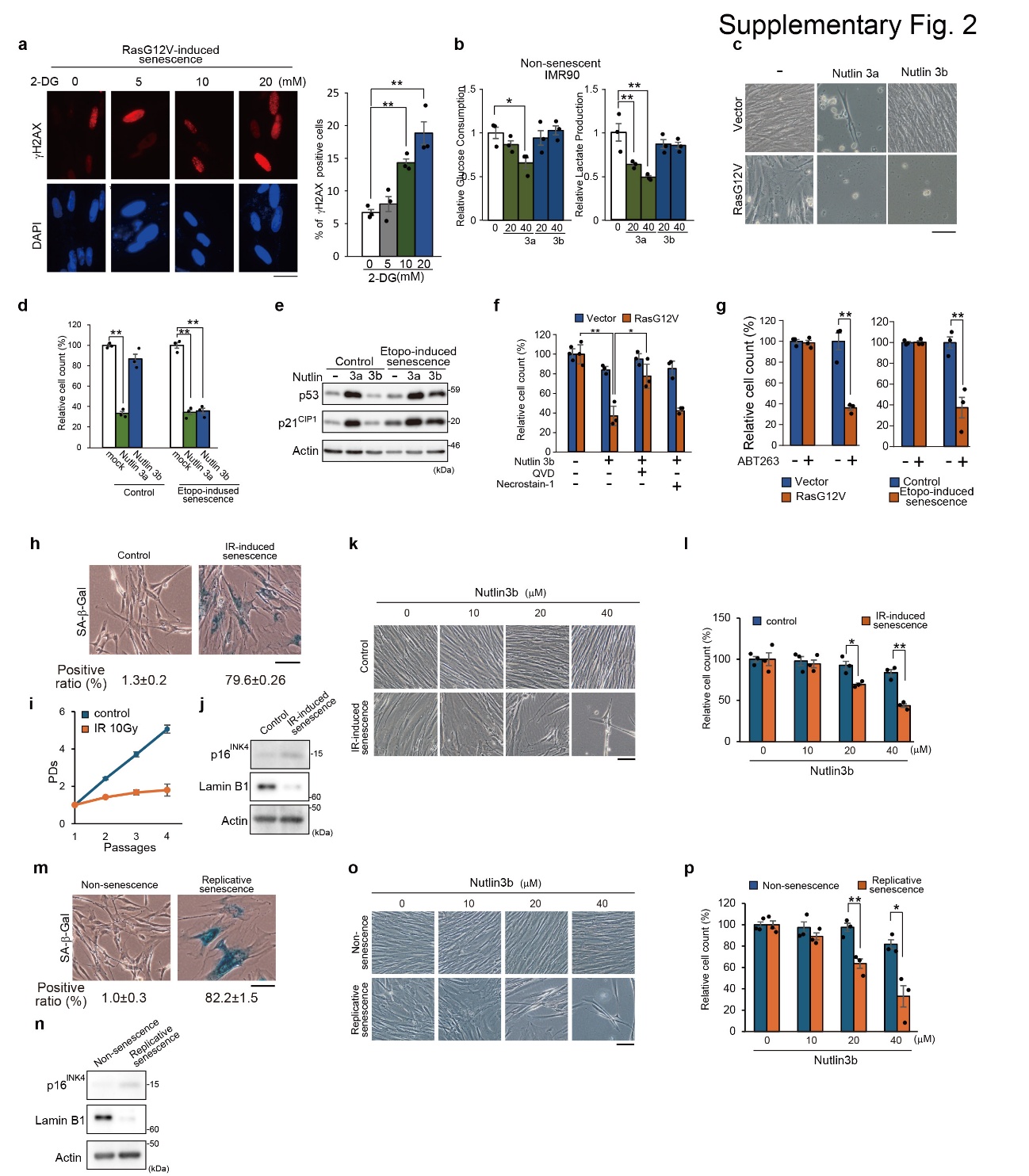
**

**Supplementary Figure 2. Senolytic effects of Nutlin 3b (Relevant to Figure 2).**

**a.** Detection of phosphorylated γH2AX at Ser-139 as a DNA damage marker. Ras G12 V-induced senescent cells were treated with 2DG. γH2AX-positive cells were counted in indicated cells (right panel). Bar indicates 20 μm. Data are representative of two independent experiments. **b.** Glucose consumption and lactate production were evaluated in non-senescent control cells with Nutlin 3a (green) or 3b (blue) treatment (n=3 biological replicates). **c.** Representative pictures of indicated cells with 40 μM Nutlin 3a, or 3b. Bar indicates 100 μm. **(d. e.)** The effect of Nutlin 3a and 3b against human primary fibroblasts during DNA-damage induced senescence. Senescent and non-senescent cells were prepared with or without etoposide treatment. These cells were exposed to Nutlin 3a or 3b for 96 h. **d.** Cell survival was assessed in indicated cells (green; Nutlin 3a, blue; Nutlin 3b) (n=3, biological replicates). **e.** p53 and p21 ^CIP1^ levels are shown in indicated cells with Nutlin 3a or 3b treatment. Data are representative of two independent experiments. **f.** Apoptosis inhibitor alleviates senolysis induced by Nutlin 3b. QVD; an inhibitor of apoptosis, Necrostatin-1; an inhibitor of necrosis (n=3, biological replicates). **g.** Senolytic effect of ABT263 on senescent cells by OIS (left panel) or by DNA damage (right panel). (**h.**-**l.**) The effect of Nutlin 3b on irradiation (IR)-induced senescence. IR-induced SnCs and non-senescent control cells were prepared. Cells were treated with Nutlin 3b. **h.** Representative pictures of SA-β-Gal staining. Averages of positivity of SA-β-Gal staining are shown. **i.** Growth curves of IR-induced SnCs. **j.** Western blotting for p16^Ink4a^ and lamin B1. **k.** Representative pictures of cells after Nutlin 3b treatment. **l.** Relative cell counts under indicated concentrations of Nutlin 3b. (**m.**-**p.**) The effect of Nutlin 3b on replicative senescence. **m.** Representative pictures of SA-β-Gal staining. Averages of positivity of SA-β-Gal staining are shown. **n.** Western blotting for p16^Ink4a^ and lamin B1. **o.** Representative pictures of cells after Nutlin 3b treatment. **p.** Relative cell counts under indicated concentrations of Nutlin 3b. Data represent the mean ± SEM. Single (*) and double (**) asterisks indicate statistical significance of p<0.05 and p<0.01, respectively. Statistical analyses were determined by unpaired Student’s two-tailed t-tests (g. and i.) or one-way analysis of variance (ANOVA) and Dunnett’s multiple comparison test.

**
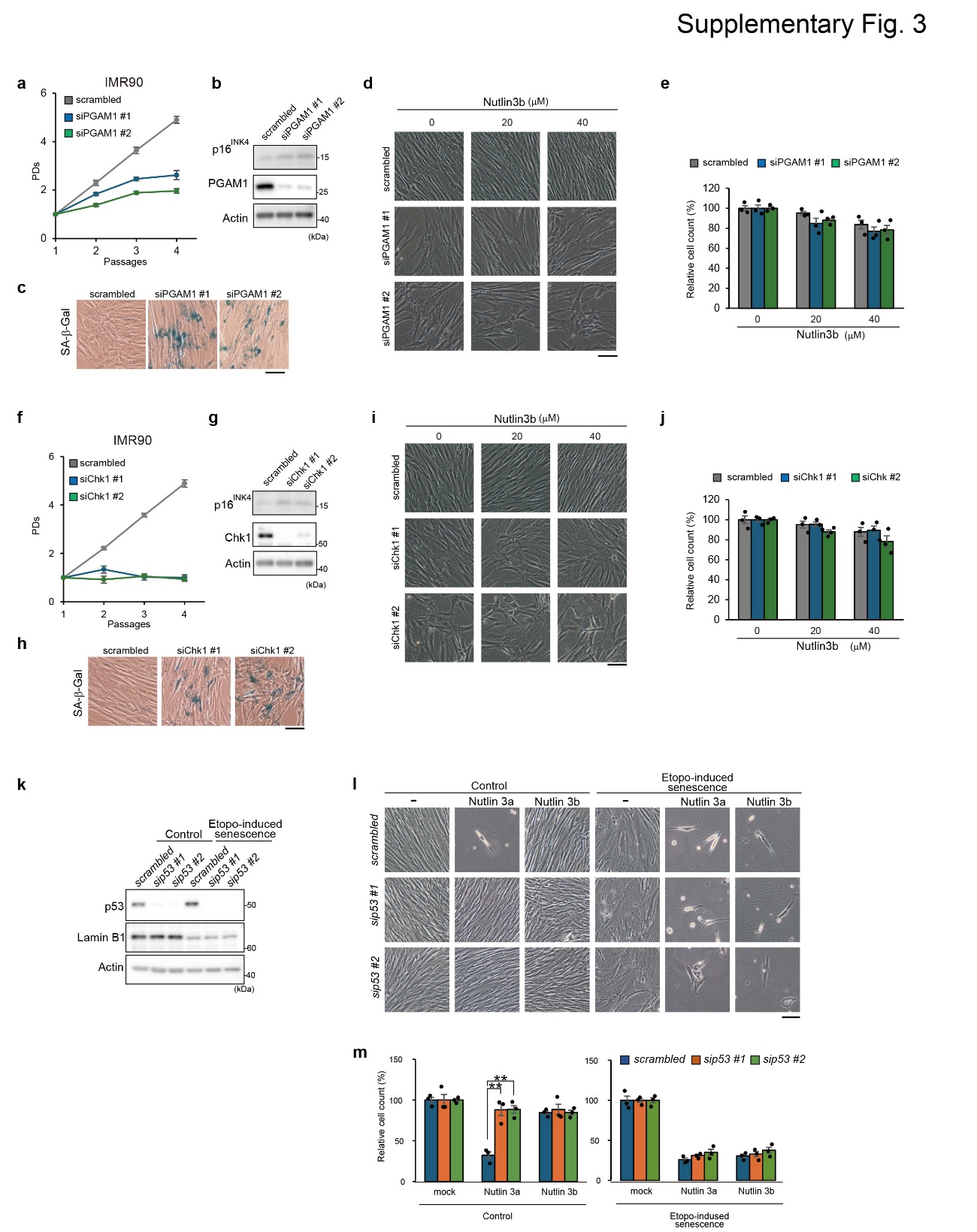
**

**Supplementary Figure 3. Senolytic effects of Nutlin 3b on PGAM1-, Chk1-, or p53-knockdown (KD) SnCs (Relevant to Figure 2).**

(**a.**-**j.**) The effect of Nutlin 3b on SnCs provoked by PGAM1- or Chk1- knockdown (KD). (**a.**-**e.**) SnCs induced by PGAM1-KD were prepared. **a.** Growth curves after Nutlin 3b treatment. **b.** Western blotting of p16^Ink4a^ and PGAM1. **c.** SA-β-Gal staining of SnCs. Averages of positivity of the staining are shown. (**d.**-**e.**) These SnCs were treated with Nutlin 3b. Representative pictures (**d.**) and cell counts (**e.**) of indicated cells after Nutlin 3b treatment. (**f.**-**j.**) SnCs induced by Chk1-KD were prepared. **f.** Growth curves after Nutlin 3b treatment. **g.** Western blotting of p16 and Chk1. **h.** SA-β-Gal staining of SnCs. Averages of positivity of the staining are shown. Representative pictures (**i.**) and cell counts (**j.**) of indicated cells after Nutlin 3b treatment. (**k.**-**m.**) The effect of p53 knockdown (KD) on Nutlin 3a or 3b-treated SnCs. **k.** Western blotting of p53 and Lamin B1 in p53-KD SnCs. **l.** Representative pictures of p53-KD SnCs after treatment of Nutlin 3a or 3b. **m.** Cell counts of indicated cells after Nutlin 3a or 3b treatment. Data represent the mean ± SEM. Single (*) and double (**) asterisks indicate statistical significance of p<0.05 and p<0.01, respectively. Statistical analyses were determined by unpaired Student’s two-tailed t-tests or one-way analysis of variance (ANOVA) and Dunnett’s multiple comparison test.

**
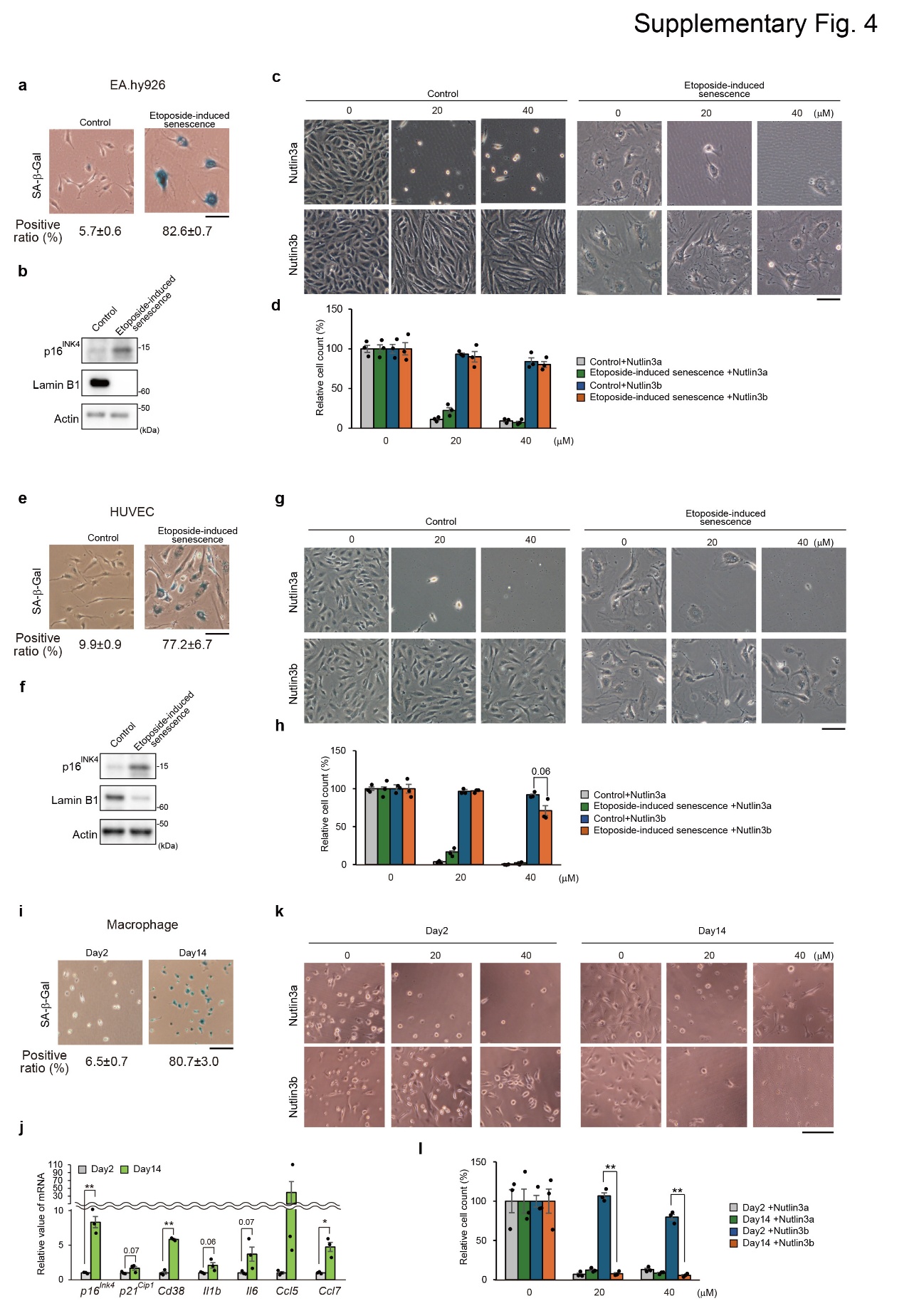
**

**Supplementary Figure 4. The effects of Nutlin 3a or 3b on SnCs of vascular endothelial cells and macrophages (Relevant to Figure 2).**

(**a.**-**j.**) The effect of Nutlin 3a or 3b on senescent human vascular endothelial cells; EA.hy926 (**a.**-**d.**) and HUVEC (**e.**-**h.**), respectively. (**a.**-**d.**) DNA damage-induce SnCs of EA.hy926 were prepared by etoposide treatment. **a.** SA-β-Gal staining. Averages of positivity of the staining are shown. **b.** Western blotting of p16^Ink4a^ and lamin B1. **c.** Representative pictures of cells after Nutlin 3a or 3b treatment. **d.** Cell counts of indicated cells after Nutlin 3a or 3b treatment. (**e.**-**h.**) DNA damage-induce SnCs of HUVEC were prepared. **e.** SA-β-Gal staining. Averages of positivity of the staining are shown. **f.** Western blotting of p16^Ink4a^ and lamin B1. **g.** Representative pictures of cells after Nutlin 3a or 3b treatment. **h.** Cell counts of indicated cells after Nutlin 3a or 3b treatment. (**i.**-**l.**) The effect of Nutlin 3a or 3b on senescent macrophage cells. Intraperitoneal primary macrophages were collected from young mice, which reached replicative senescence after 14 days culture *in vitro*. **i.** SA-β-Gal staining of macrophages. Averages of positivity of the staining are shown. **j.** Evaluation of mRNAs for senescent parameters by RT-PCR. Representative pictures (**k.**) and cell counts (**l.**) of indicated cells after Nutlin 3a or 3b treatment. Data represent the mean ± SEM. Single (*) and double (**) asterisks indicate statistical significance of p<0.05 and p<0.01, respectively. Statistical analyses were determined by unpaired Student’s two-tailed t-tests or one-way analysis of variance (ANOVA) and Dunnett’s multiple comparison test.

**
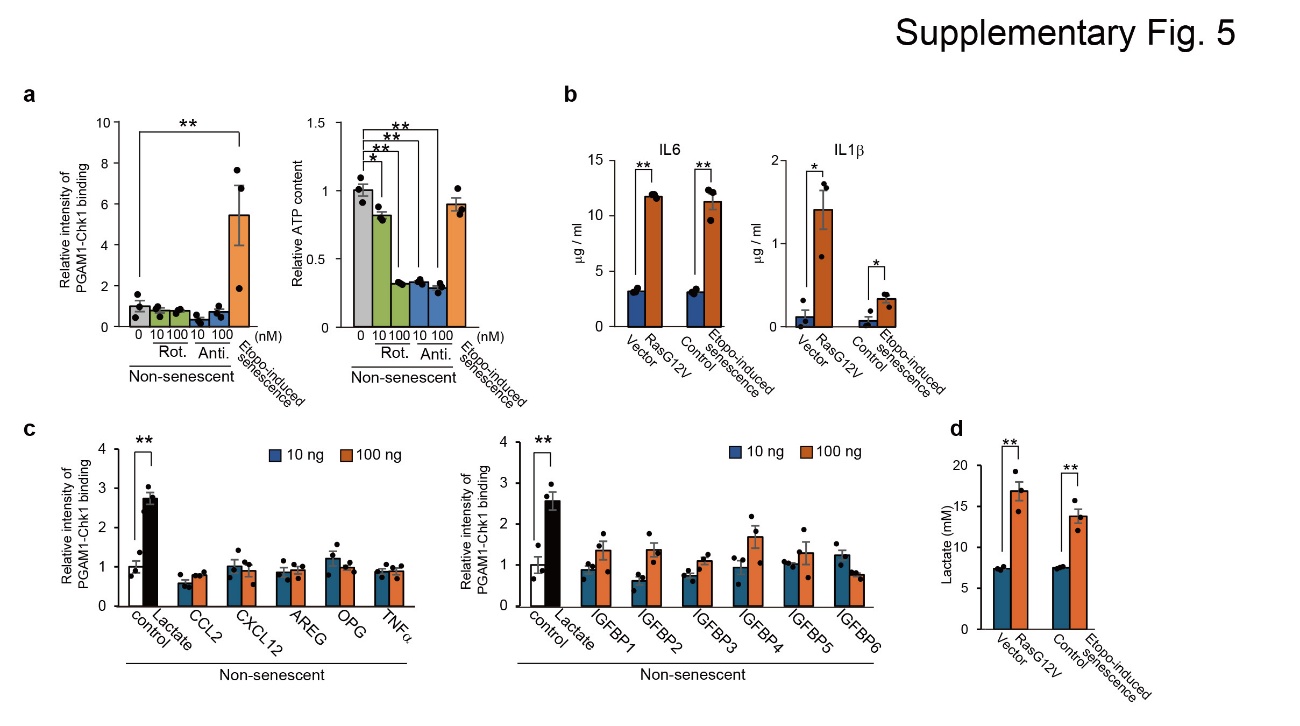
**

**Supplementary Figure 5. NanoBiT assay for PGAM1-Chk1 binding under mitochondrial inhibitors or SASP factors (Relevant to Figure 2).**

**a.** NanoBiT assay to detect PGAM1-Chk1 binding in non-senescent IMR90 cells (n=3, biological replicates). After 24 h treatment with rotenone (Rot.) or antimycin (Anti.), cells were collected for NanoBiT assay (left panel). ATP levels of indicated cells were measured by CellTiter-Glo (right panel). These values were normalized by CellTiter-Fluor. **b.** ELISA assay evaluated the levels of IL6 and IL1b in culture medium from indicated cells. **c.** The effects of SASP factors on PGAM-Chk1 binding. Non-senescent cells expressing PGAM-Chk1-NanoBiT were prepared and treated with indicated recombinant SASP proteins or lactate. NanoBiT assay were performed. **d.** Evaluation of lactate concentration in the culture of senescent and non-senescent cells. Data represent the mean ± SEM. Single (*) and double (**) asterisks indicate statistical significance of p<0.05 and p<0.01, respectively. Statistical analyses were determined by unpaired Student’s two-tailed t-tests or one-way analysis of variance (ANOVA) and Dunnett’s multiple comparison test.

**
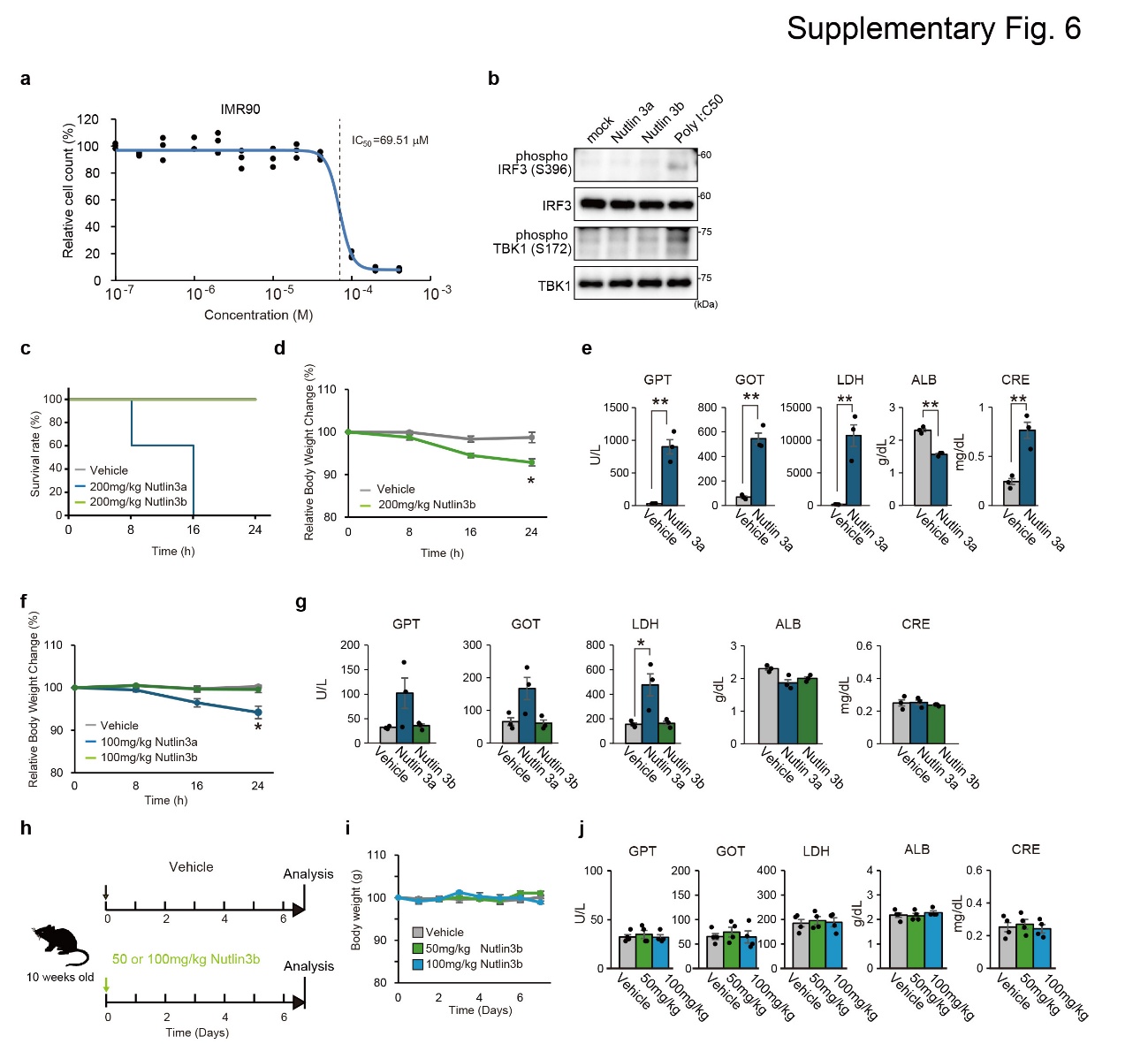
**

**Supplementary Figure 6. Short-term toxicity assessments of Nutlin 3b (Relevant to Figure 3).**

**a.** IC50 of Nutlin-3b in IMR90 cells. Primary IMR90 cells are exposed to increasing amounts of Nutlin 3b. IC50 of Nutlin 3b is evaluated. **b.** The effect of Nutlin 3a and 3b on DNA sensing immune pathway in non-senescent cells. Cells were treated with polyinosinic:polycytidylic acid (Poly I:C), stimulatot for toll-like receptor 3 (TLR3), followed by activation of cGAS-STING pathway. Phosphorylation of IRF3 and TBK1 proteins were evaluated by western blotting. (**c.**-**j.**) Short-term treatment of young mice at ten weeks old by Nutlin 3a or 3b. Mice were injected once with high-dose drugs; 200mg/kg (**c.**-**e.**) and 50 or 100mg/kg (**f.**-**j.**). **c.** Survival of mice within 24 hours after injection of drugs at 200mg/kg. Each group comprises mice with n=5. **d.** Body weights of mice after injection of Nutlin 3a or 3b at 200mg/kg. **e.** Blood parameters of mice after injection of Nutlin 3a at 200mg/kg. Each group comprises mice with n=3. (**f.**-**g.**) Comparison of one-shot treatment with Nutlin 3a and 3b at 100mg/kg. Each group comprises mice with n=3. **f.** Body weights of mice after injection of drugs at 100mg/kg. **g.** Comparison of blood parameters of mice. (**h.**-**j.**) Outcome of one-shot injection of Nutlin 3b at 50 or 100mg/kg. Each group comprises mice with n=4. **h.** Protocol of mice injected with Nutlin 3b at 50 or 100mg/kg. **i.** Body weights during seven days after injection. **j.** Blood parameters of mice after injection of Nutlin 3b at 50 or 100mg/kg. Data represent the mean ± SEM. Single (*) and double (**) asterisks indicate statistical significance of p<0.05 and p<0.01, respectively. Statistical analyses were determined by unpaired Student’s two-tailed t-tests or one-way analysis of variance (ANOVA) and Dunnett’s multiple comparison test.

**
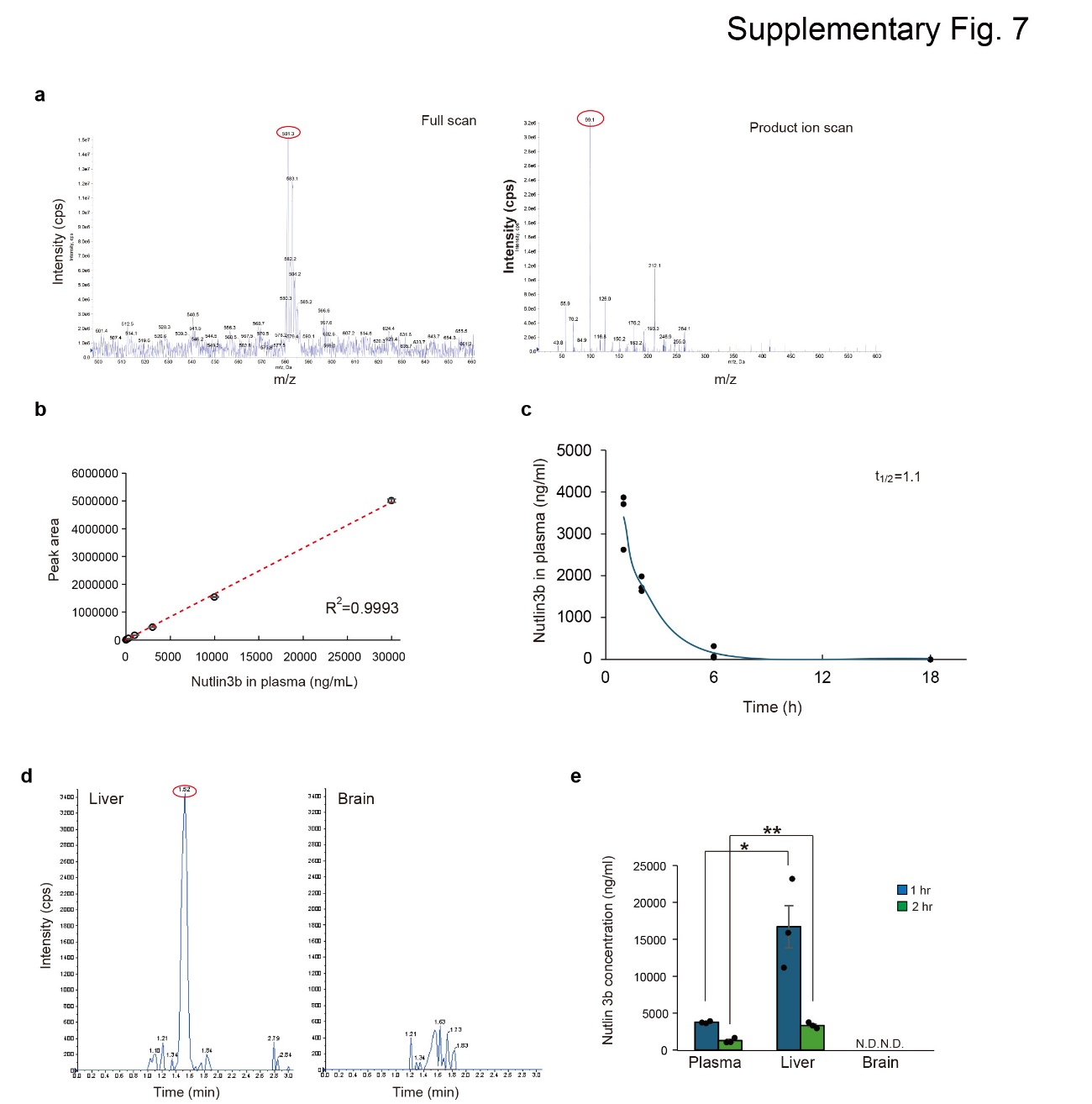
**

**Supplementary Figure 7. Evaluation of the pharmacokinetics of Nutlin 3b using LC-MS/MS (Relevant to Figure 3).**

**a.** Indentification of Nutlin 3b by mass spectrometry. The left panel shows the full scan spectrum, and the right panel shows the product ion scan spectrum. **b.** Linear regression analysis of peak areas versus the concentrations of Nutlin 3b spiked into plasma. **c.** Evaluation of plasma Nutlin 3b levels after intraperitoneal injection (11.62 mg/kg) into mice (n=3). Blood samples were collected at the indicated time points. **d.** Detection of the LC-MS/MS peak corresponding to Nutlin 3b in liver and brain tissues. A peak at 1.5 minutes corresponding to Nutlin 3b was detected in 1 μg of liver tissue, whereas no significant peak was observed in 100 μg of brain tissue. **e.** Comparison of Nutlin 3b levels in plasma, liver, and brain of mice after injection. N.D.; not detected. Data represent the mean ± SEM. Single (*) and double (**) asterisks indicate statistical significance of p<0.05 and p<0.01, respectively. Statistical analyses were determined by unpaired Student’s two-tailed t-tests.

**
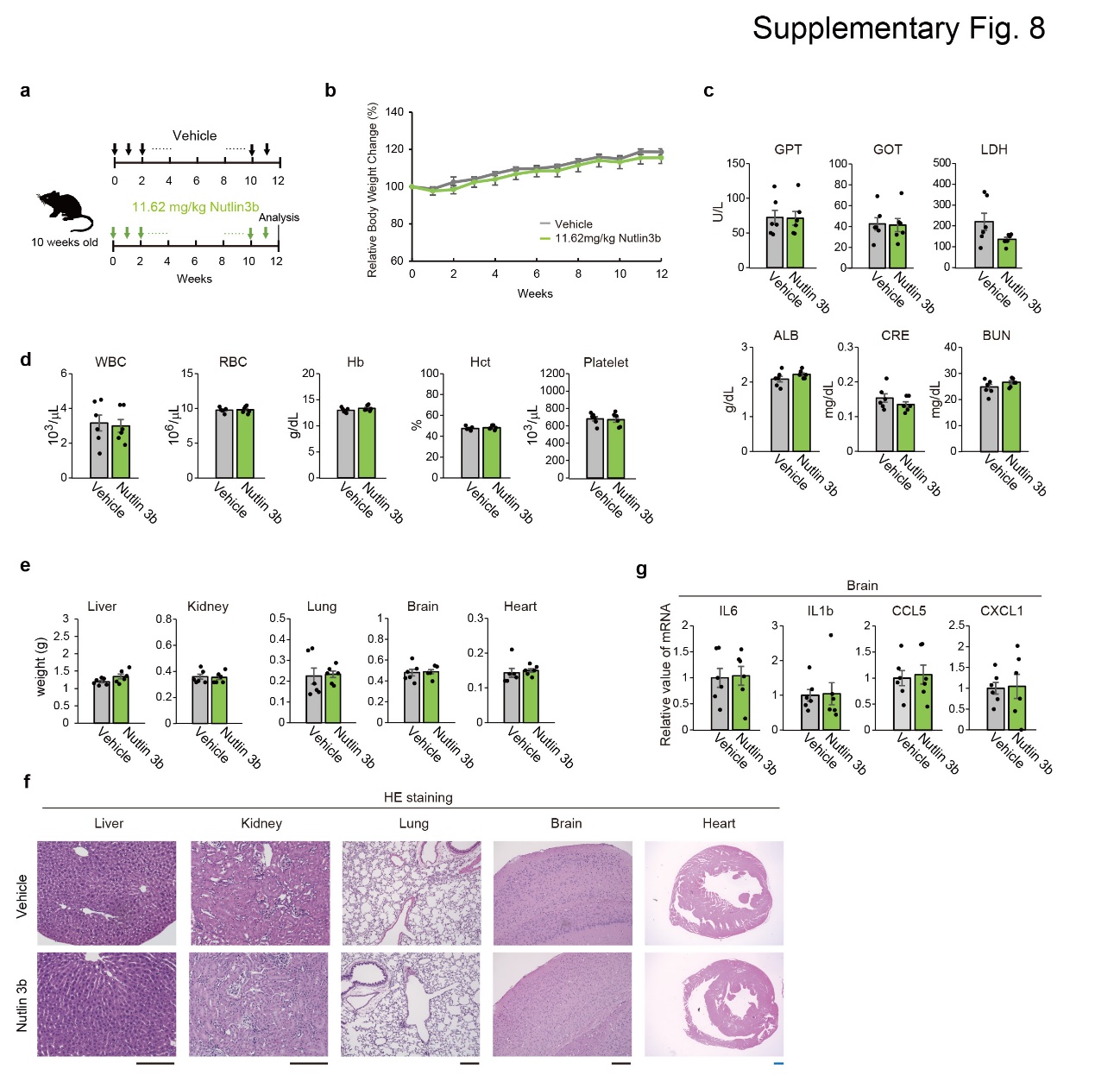
**

**Supplementary Figure 8. Long-term toxicity assessments of Nutlin 3b (Relevant to Figure 3).**

**a.** Protocol of the injection of Nutlin 3b (11.62 mg/kg) into young mice every week for three months. Each group comprises six mice (female n=3 and male n=3). **b.** Body weights of mice during Nutlin 3b treatment for three months. (**c.**-**d.**) Blood parameters of mice after long-term treatment of Nutlin 3b. **c.** Biochemical examination of blood. **d.** Blood cell counts. **e.** The comparison of tissue weights between vehicle and Nutlin 3b treated after three-month assessment**.** **f.** Representative pictures of HE staining of tissues after long-term treatment. Black and blue bar indicate 200 μm and 500 μm, respectively. **g.** RT-PCR analysis of SASP factors in brain after Nutlin 3b treatment for three months.


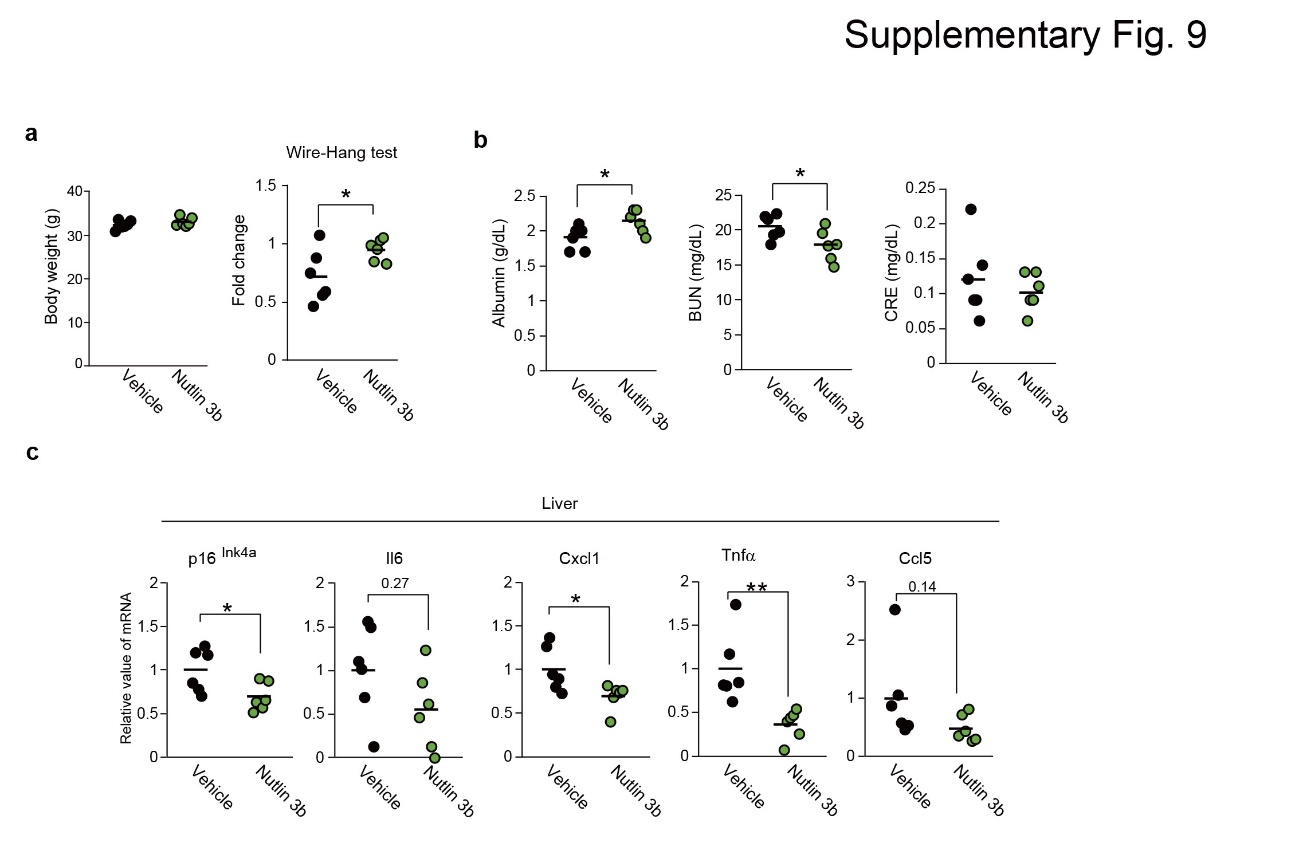


**Supplementary Figure 9. Administration of Nutlin 3b in aged mice (Relevant to Figure 3).**

Administration of Nutlin 3b in aged male mice for three months. Several physiological parameters were assessed one week after Nutlin 3b treatment (n=6, mice). **a.** Body weight and wire-hang test in aged male mice with or without Nutlin 3b treatment. **b.** Blood parameters in aged male mice with or without Nutlin-3b treatment. Plasma albumin (left panel), lactate BUN (middle) and BUN creatinine (CRE) levels (right panel).　**c.** Assessment of mRNA levels of p16^Ink4a^ and proinflammatory factors in liver tissues with or without Nutlin 3b treatment. Data represents the mean ± SEM. Single (*) and double (**) asterisks indicate statistical significance of p<0.05 and p<0.01, respectively. Statistical analyses were determined using unpaired Student’s two-tailed t-tests.

**
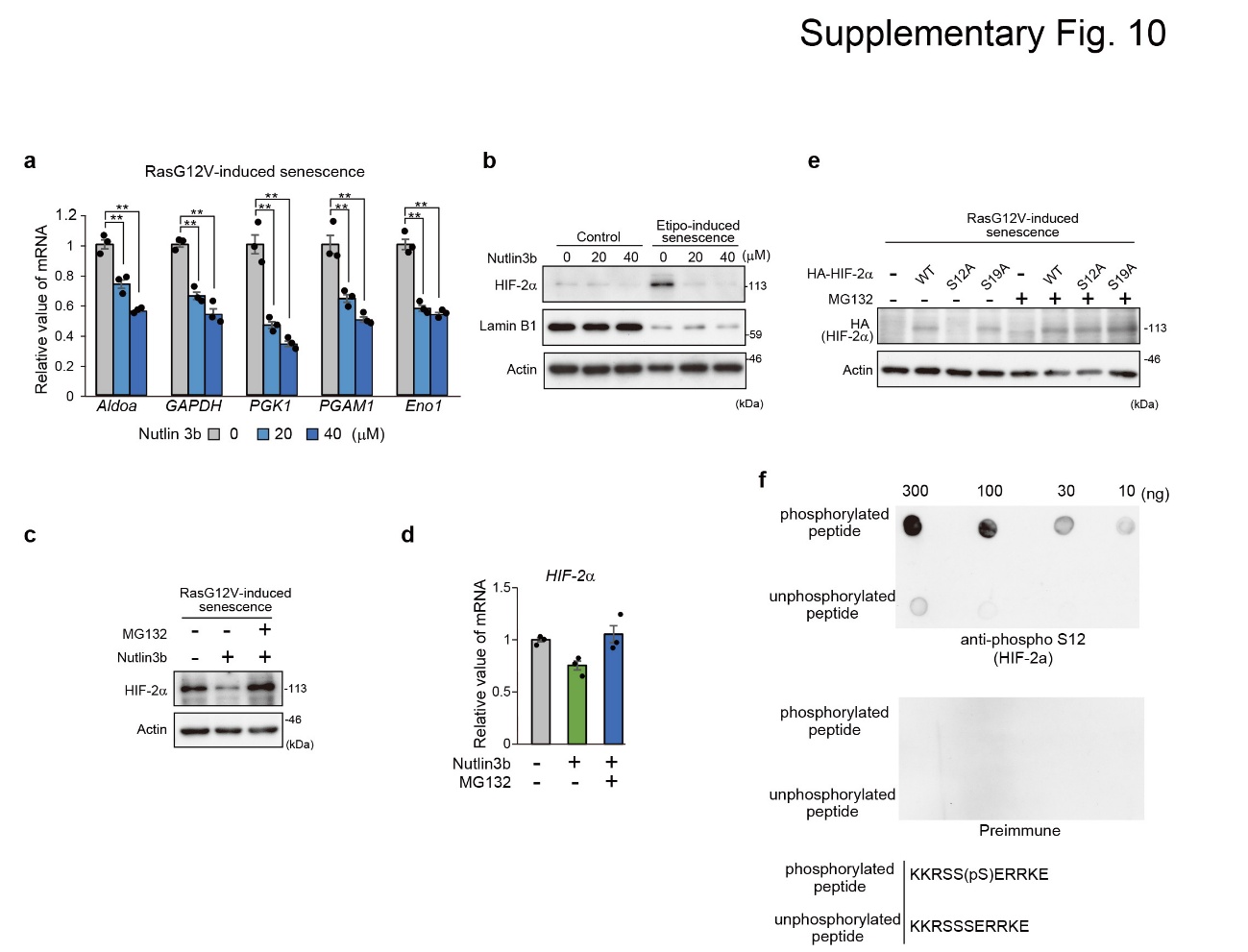
**

**Supplementary Figure 10. The interference to PGAM1-Chk1 binding downregulates HIF-2α protein (Relevant to Figure 4).**

**a.** Assessment of mRNA levels for glycolytic enzymes after Nutlin 3b treatment. Senescent cells were prepared as described in Figure 4A (n=3, biological replicates). **b.** The effect of Nutlin 3b on HIF-2α protein in etoposide-induced SnCs. Protein levels of HIF-2α are shown by western blot. Senescent and control cells were treated with Nutlin 3b for 48 h. (**c, d.**) The effect of Nutlin 3b on HIF-2α protein. In addition to Nutlin3b, SnCs were treated with or without MG132, a proteasome inhibitor. **c.** The effect of Nutlin 3b on HIF-2α protein with or without MG132. **d.** HIF-2α mRNA levels were evaluated in SnCs (n=3, biological replicates). **e.** Evaluation of HIF-2α proteins with or without mutations at Ser-12 or Ser-19. Indicated versions of HA-tagged HIF-2α (WT, S12A or S19A) were ectopically expressed in SnCs. Cells with or without MG132 treatment were collected for western blotting. **f.** Validation of anti-phospho-S12-HIF-2α antibody. Dot blot analysis by anti-phospho-S12 antibody (upper panel) or by pre-immune serum (lower panel). Different concentrations of indicated peptides were dot plotted. Data are representative of two independent experiments. Data represent the mean ± SEM. Single (*) and double (**) asterisks indicate statistical significance of p<0.05 and p<0.005, respectively. Statistical analyses were performed using one-way analysis of variance (ANOVA) and Dunnett’s multiple comparison test.


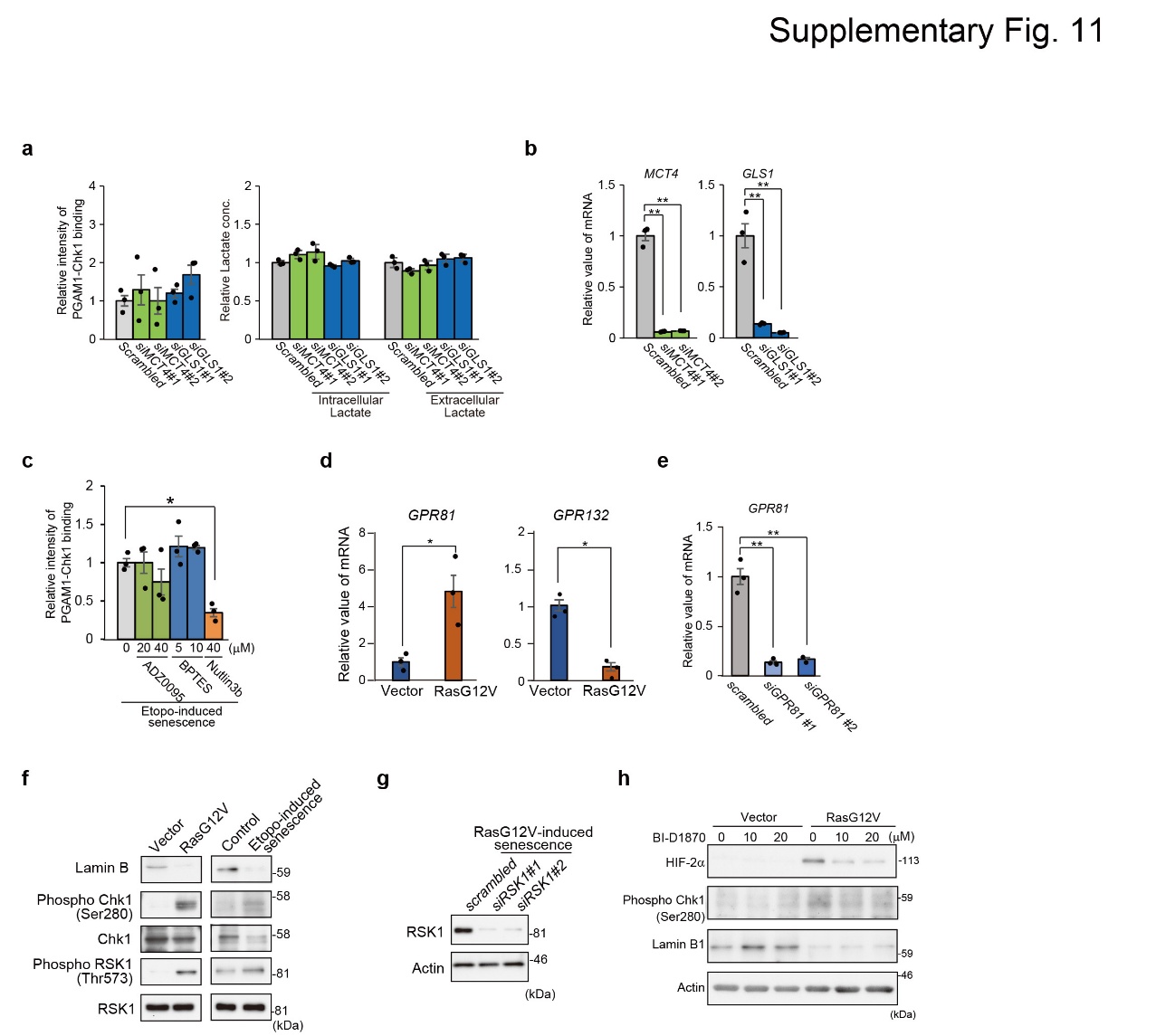


**Supplementary Figure 11. Lactate promotes PGAM1-Chk1 binding (Relevant to Figure 4).**

(**a.**-**c.**) The effect of inhibiting MCT4 (lactate uptake transporter) or GLS1 (glutaminase 1) on PGAM-Chk1 interaction. SnCs expressing PGAM-Chk1-NanoBiT were prepared, which were transfected with indicated specific siRNAs (**a.**-**b.**) or treated with indicated inhibitors (**c.**). **a.** PGAM-Chk1-NanoBiT assay (left panel) and lactate evaluation in their medium (right). **b.** RT-PCR quantification of the indicated mRNA levels after the knockdown by MCT4 or GLS1 siRNA. **c.** The effect of inhibitor of MCT4 or GLS1 on PGAM-Chk1 interaction. SnCs with PGAM-Chk1-NanoBiT were treated with indicated drugs; ADZ0095 and BPTES as inhibitor for MCT4 and GLS1, respectively. **d.** Comparison of GPR81 and GPR132 mRNA between control and SnCs by RT-PCR (n=3, biological replicates). **e.** GPR81 mRNA levels were evaluated after its siRNA knockdown in indicated cells of Fig. 4H. **f.** Activation of RSK1 kinase in SnCs. Senescence was induced by oncogenic Ras (left panels) or by DNA damage (right panels). Western blot probed by the antibodies against Chk1, RSK1, phospho-Chk1-S280, and phospho-RSK1-Thr573 in indicated cells. Data are representative of two independent experiments. **g.** The effect of RSK1 knockdown in oncogene-induced SnCs. The indicated extracts of Fig. 4K were probed by indicated antibodies. **h.** Inactivation of Rsk1 kinase downregulates HIF-2α protein and Chk1 phosphorylation in S280 in SnCs. Control or senescent cells were exposed to BI D1870, an inhibitor of Rsk1. All western blots were repeated twice and representative data are shown. Data represent the mean ± SEM. Single (*) and double (**) asterisks indicate statistical significance of p<0.05 and p<0.01, respectively. Statistical analyses were performed with unpaired Student’s two-tailed t-tests or one-way analysis of variance (ANOVA) and Dunnett’s multiple comparison test.

**
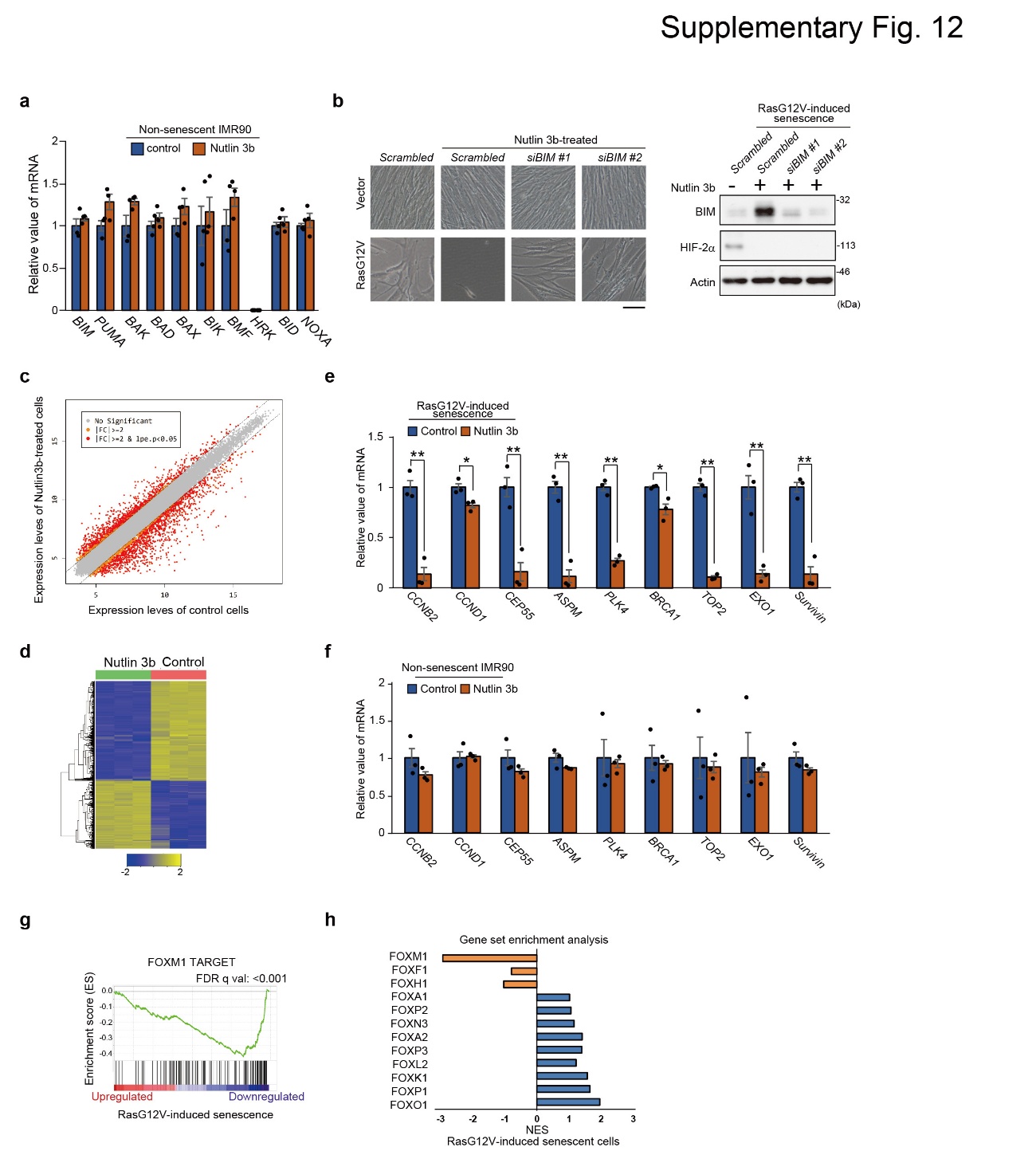
**

**Supplementary Figure 12. PGAM1-Chk1 antagonist suppresses FOXM1 in senescence (Relevant to Figure 5).**

**a.** Assessment of the pro-apoptotic BH family in early passage cells after Nutlin 3b treatment using RT-PCR analysis (n=3, biological replicates). **b.** Representative pictures of BIM-knockdown cells (left panel). Bar indicates 100 μm (n=3, biological replicates). Validation of BIM knockdown (right panel). Western blotting was performed for the extract from indicated cells. **c.** Comparison by microarray analysis between control and Nutlin 3b treatment in SnCs (n=3, biological replicates). Gray dots are genes with no statistical significance. Genes with FC≥2 or with FC≥2 & lpe p>0.05 are presented as orange or red dots, respectively. **d.** Two-way, hierarchical clustering heatmap using Z-scores of normalized values. 2,105 probes with FC≥2 & lpe p>0.05 are presented. Upregulated and downregulated mRNAs are shown in yellow and blue, respectively. (**e. and f.)** The profile of mRNAs for FOXM1 targets after Nutlin 3b treatment in SnCs (**e**) and in non-senescent cells (**f**); CCNB2, CCND1, CEP55, ASPM, PLK4, BRCA1, TOP2, EXO1and Survivin (n=3, biological replicates). **g.** Gene set enrichment analysis (GSEA) for FOXM1 targets in Nutlin 3b-treated SnCs. **h.** Gene set enrichment analysis (GSEA) for FOX family proteins. NES values for each FOX member are shown. Data represent the mean ± SEM. Single (*) and double (**) asterisks indicate statistical significance of p<0.05 and p<0.01, respectively. Statistical analyses were determined using unpaired Student’s two-tailed t-tests.

**
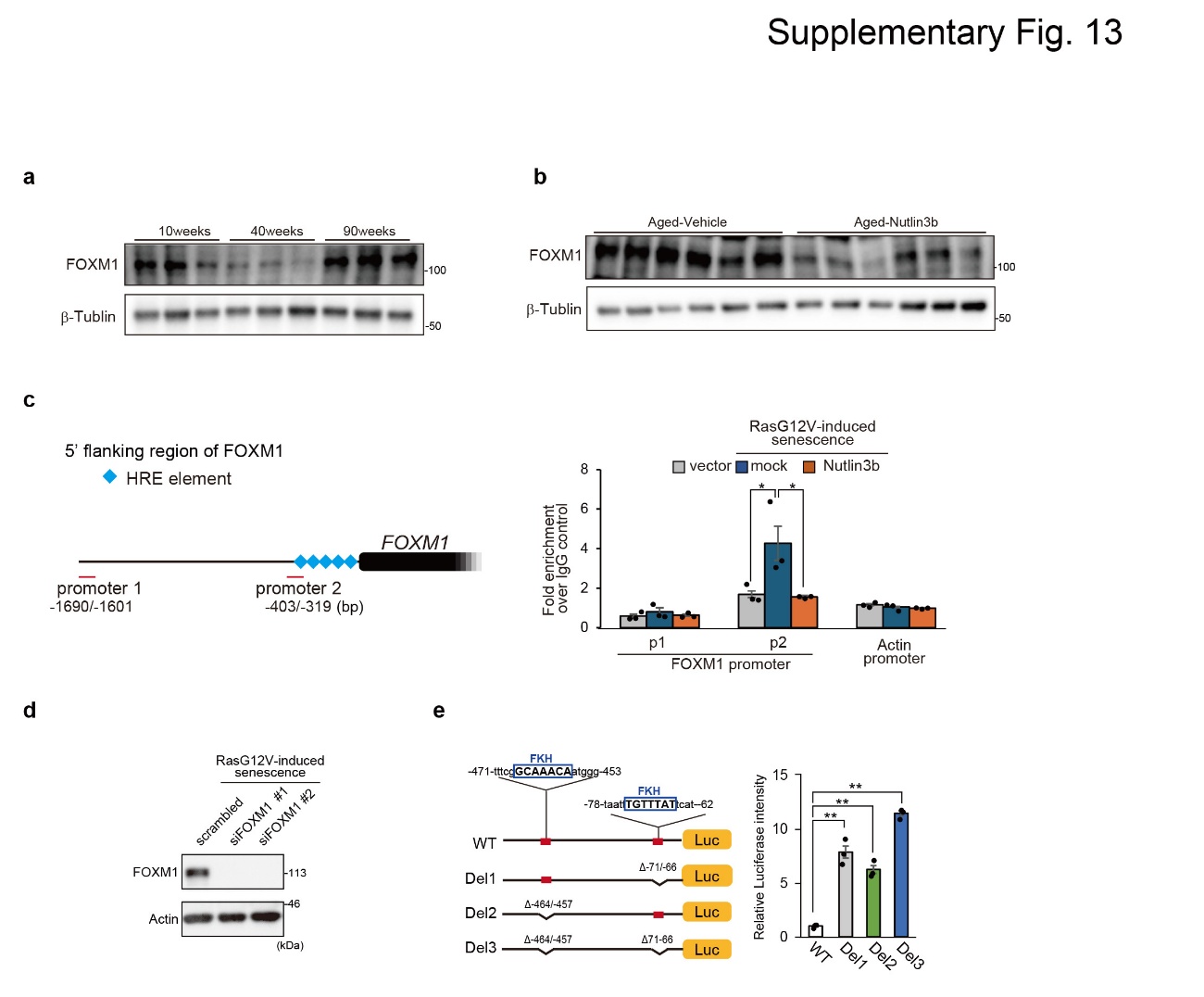
**

**Supplementary Figure 13. Pro-apoptotic BIM is provoked in senolysis by a PGAM1-Chk1 antagonist. (Relevant to Figure 5).**

(**a.**-**b.**) Evaluation of FOXM1 proteins in young and aged liver of mice with or without Nutlin 3b treatment. **a.** Comparison of FoxM1 protein levels in livers among young (10 weeks), middle-aged (40 weeks), and old (90 weeks) mice. **b.** Western blotting of FoxM1 in aged livers from vehicle- or Nutlin 3b-treated mice. **c.** Chromatin immunoprecipitation (ChIP)-qPCR assay of HIF-2α binding against FoxM1 promoter. HREs (Hypoxia responsive element) are located at about 500 bp upstream of FoxM1 ORF (left panel). Primer sets were designed for the promoter of FoxM1 and Actin; promoter-1 and -2 (p1 and p2) sets for 1690 bp and 400bp of upstream of FoxM1 promoter, respectively. Non-senescent and senescent cells (SnCs) were prepared. The qPCR assay by these primer sets were performed against indicated samples from ChIP by anti HIF-2α antibody or IgG control (right panel). **d.** Validation of FOXM1 knockdown in senescent cells. Western blotting was performed. Data are representative of two independent experiments. **e.** Luciferase assay of FKH mutants promoters in SnCs. Indicated promoters of WT and FKH mutants were generated by PCT-based mutagenesis (left panel). Indicated reporter plasmids were transfected in SnCs for luciferase assay (right panel). Data are representative of two independent experiments. Data represent the mean ± SEM. Single (*) and double (**) asterisks indicate statistical significance of p<0.05 and p<0.01, respectively. Statistical analyses were performed using one-way analysis of variance (ANOVA) and Dunnett’s multiple comparison test.

**
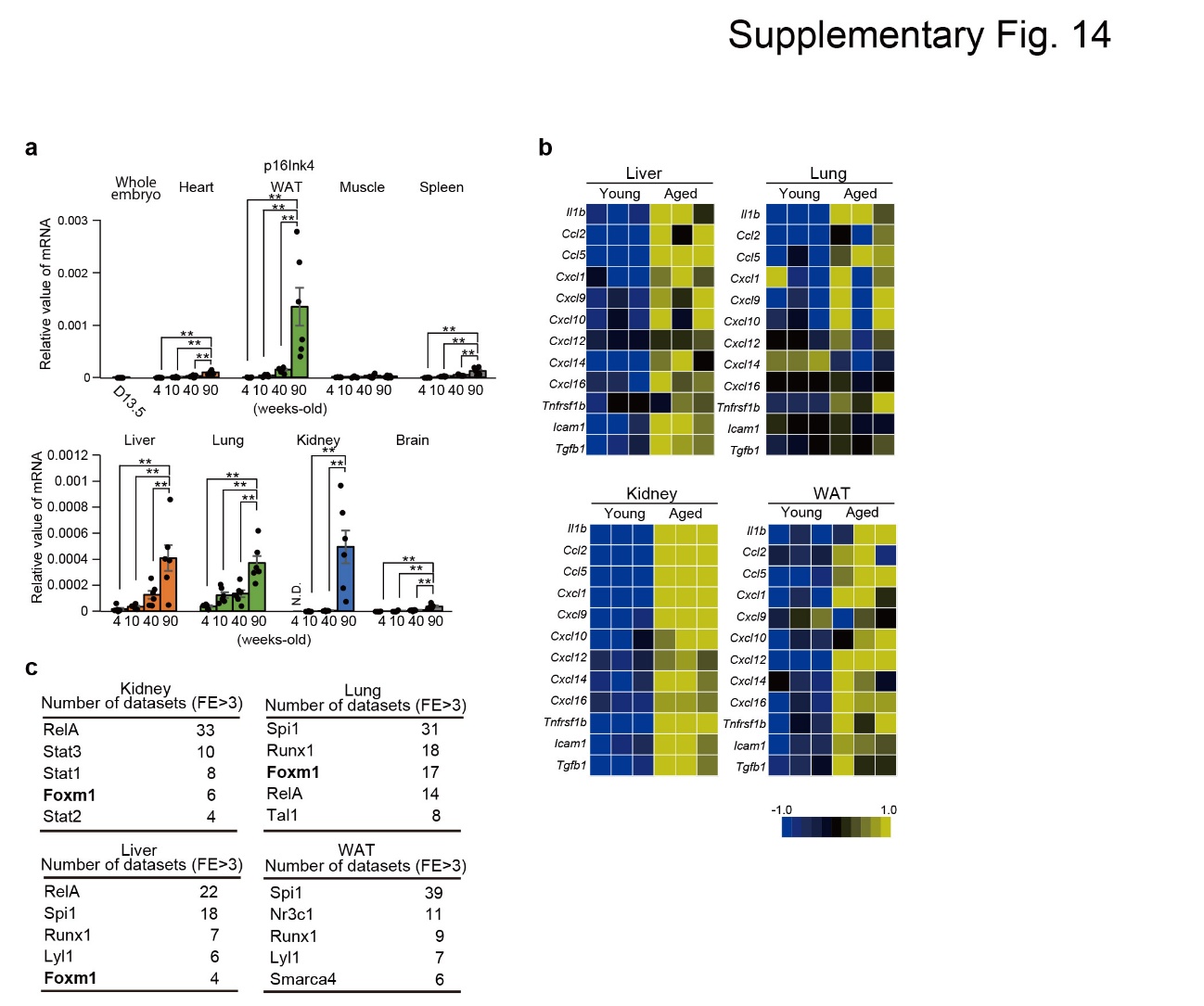
**

**Supplementary Figure 14. FOXM1 targets are upregulated in aged tissues. (Relevant to Figure 6).**

**a.** The *in vivo* profiles of p16^Ink4a^ mRNA in several tissues of 4, 10, 40, and 90 week-old mice, in addition to embryo at day 13.5. Indicated tissues are collected for RNA extraction. **b.** The heatmap analysis of SASP genes between young and aged mice. Indicated tissues were collected for transcriptomic analysis. **c.** Top5 transcriptional factors in indicated tissues were listed, according to high hit scores by fold enrichment analysis of comparative transcriptomic data. Data represent the mean ± SEM. Single (*) and double (**) asterisks indicate statistical significance of p<0.05 and p<0.01, respectively. Statistical analyses were performed using one-way analysis of variance (ANOVA) and Dunnett’s multiple comparison test.

**
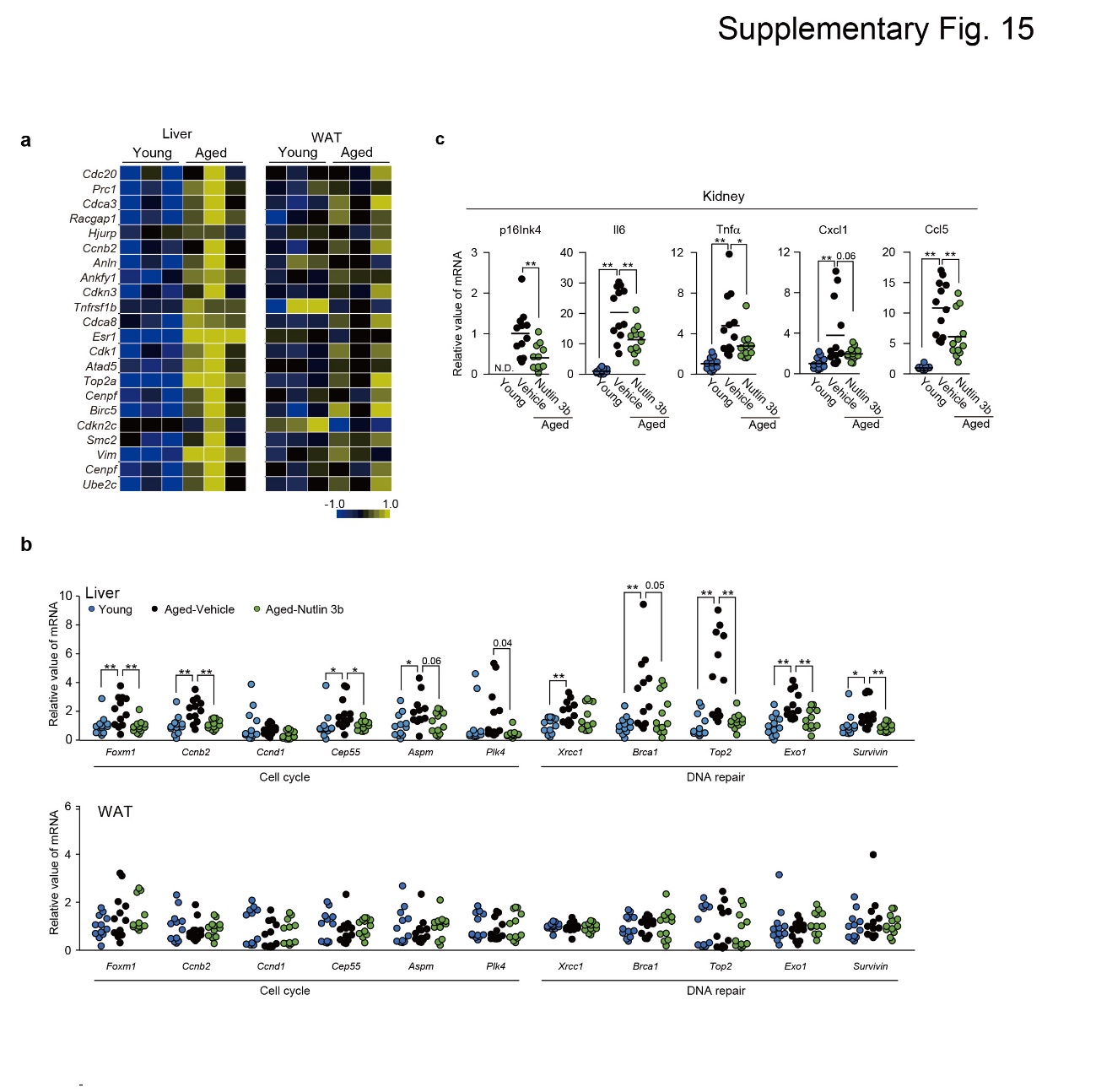
**

**Supplementary Figure 15. FOXM1 targets are upregulated in aged tissues. (Relevant to Figure 6).**

**a.** The heatmap analysis of Foxm1 target genes in liver and WAT between young and aged mice, relevant to Fig.6c. **b.** RT-PCR assessment of Foxm1 target genes, including cell cycle and DNA repair genes, in aged liver and WAT (upper and lower panel) after Nutlin 3b treatment, relevant to Fig. 6d. Tissues were collected from young, aged and Nutlin 3b-treated aged mice. **c**. Comparison of mRNA levels for p16^Ink4a^ and SASP factors (IL6, Tnfα, Ccxl1, and Ccl5), relevant to Fig. 6e. Kidney extracts from indicated mice were analyzed by RT-PCR. Data represent the mean ± SEM. Single (*) and double (**) asterisks indicate statistical significance of p<0.05 and p<0.01, respectively. Statistical analyses were performed using one-way analysis of variance (ANOVA) and Dunnett’s multiple comparison test.

**
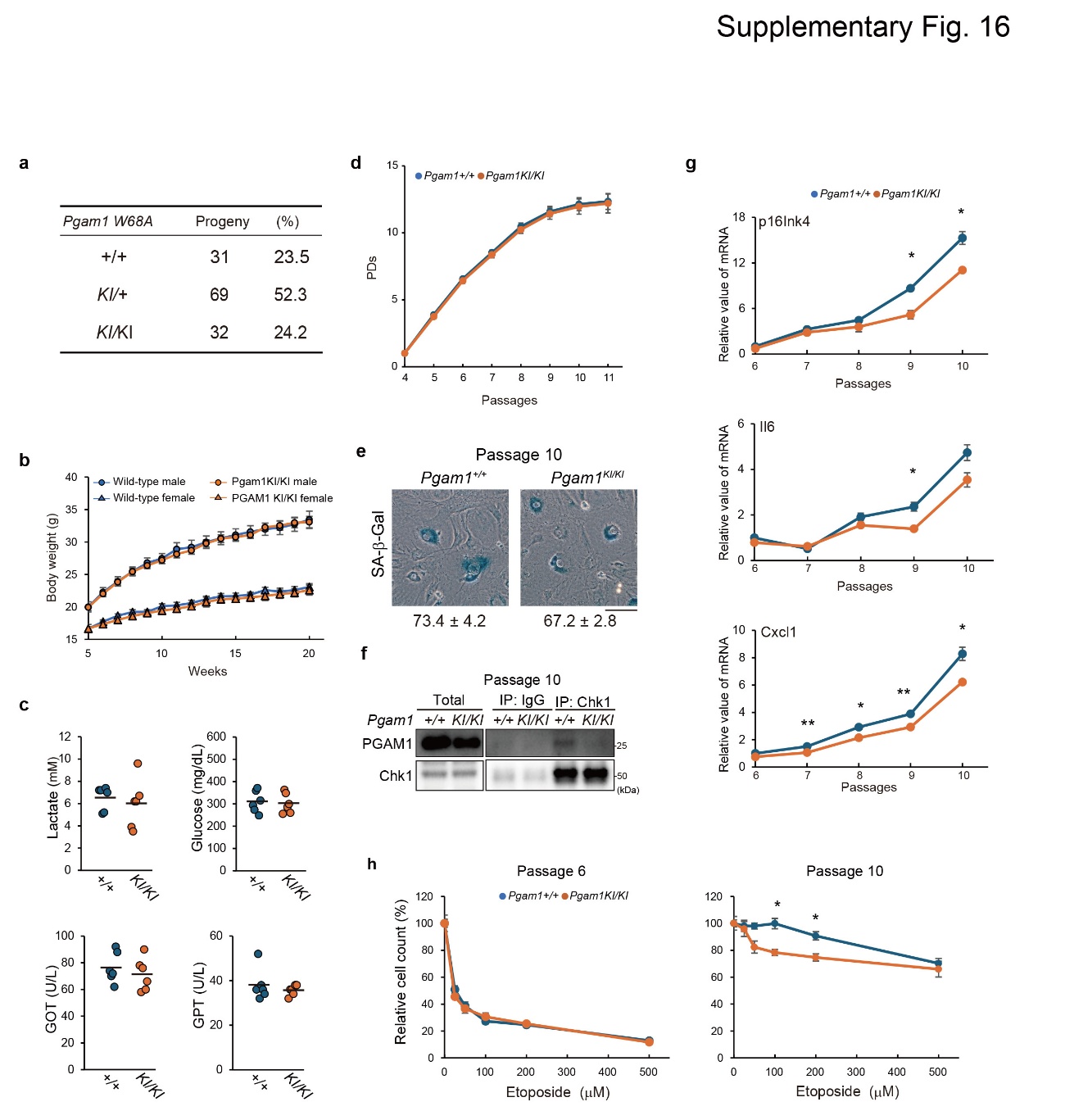
**

**Supplementary Figure 16. Characterization of MEFs from *Pgam1 ^KI/KI^* mice. (Relevant to Figure 7).**

(**a.**-**c.**) Construction of *Pgam1 W68A* knock-in (KI) mice by using the CRISPR/Cas9 system. **a.** Summary of *Pgam1* genotypes for 132 progenies from crosses between *Pgam1 ^KI/+^*mice. +/+; wild-type, KI/+; heterozygous KI, KI/KI; homozygous KI. **b.** Body weight of indicated mice until 20 weeks-old. **c.** Evaluation of indicated blood parameters in *Pgam1 ^KI/KI^* mice. **d.** Growth curves of MEFs from *Pgam1 ^+/+^* and *^KI/KI^* mice. Proliferative potentials are shown as numbers of population doublings (n=3, biological replicates). **e.** SA-β-Gal staining of wild-type or *Pgam1^KI/KI^* MEFs at passage 10. Bar indicates 100 μm **f**. Immunoprecipitation assay detection of the interactions between endogenous PGAM1 and Chk1 proteins in indicated MEFs. Wild-type or *Pgam1^KI/KI^* MEFs at passage 10 were collected after 6-h treatment with MG132. Indicated lysates were immunoprecipitated with anti-Chk1 antibody. **g.** Comparison of mRNA levels for p16^Ink4a^ and SASP factors (Il6 and Cxcl1) between wild-type and *Pgam1^KI/KI^* MEFs at indicated passages. **h.** Comparison of survival ability under DNA damage at early or senescent stage; passage 6 (left panel) and ten (right panel), respectively. Wild-type or *Pgam1^KI/KI^* MEFs were treated with etoposide at the indicated concentrations for 24 hours. Data represent the mean ± SEM. Single (*) and double (**) asterisks indicate statistical significance of p<0.05 and p<0.01, respectively. Statistical analyses were performed using unpaired Student’s two-tailed t-tests.

**
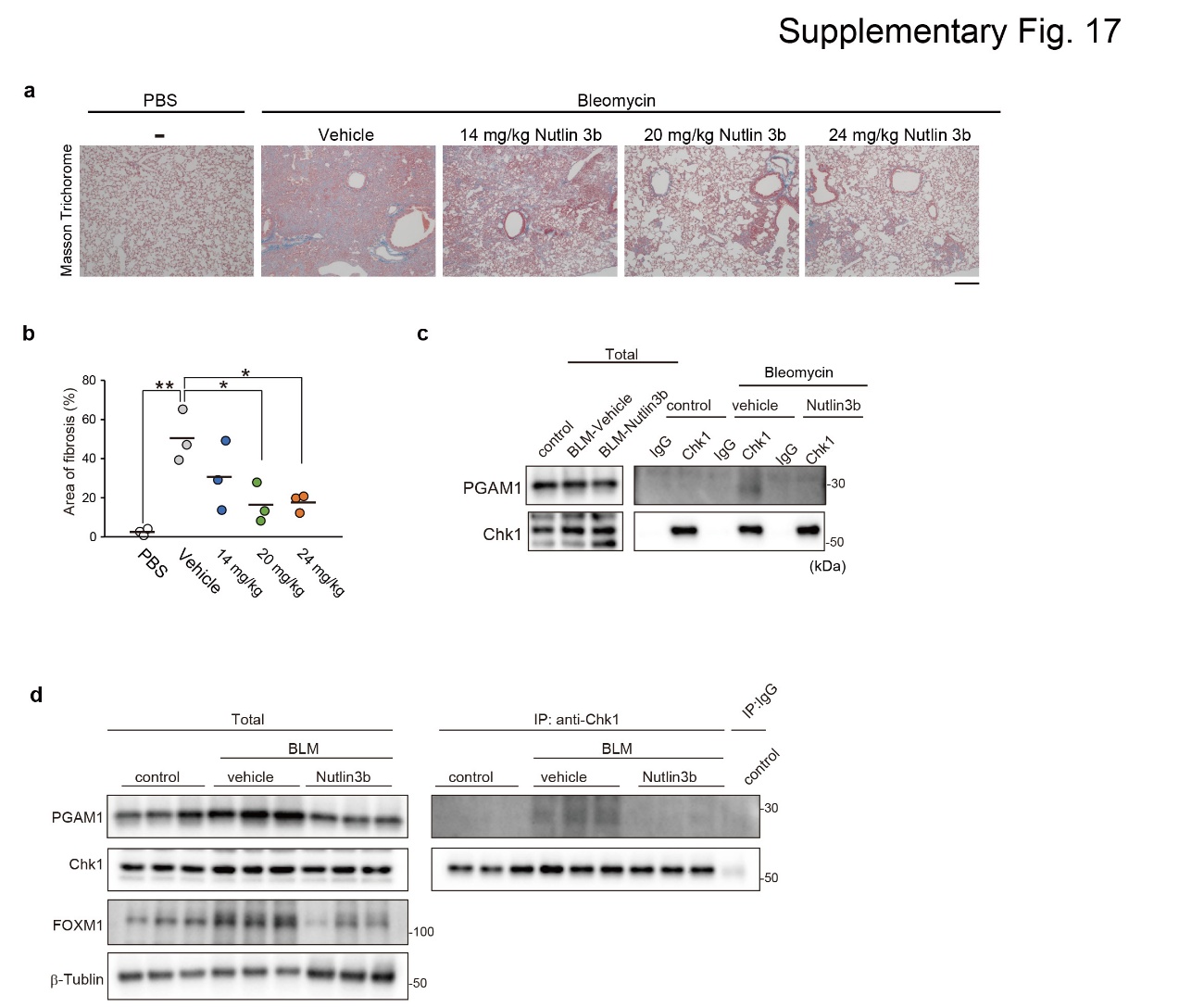
**

**Supplementary Figure 17. Verification of the therapeutic effects of Nutlin 3b in a beomycin-induced pulmonary fibrosis model (Relevant to Figure 7).**

2.5mg/kg bleomycin was administered intratracheally to CD1 mice (**a**-**b**). Indicated concentrations of Nutlin 3b were administered intraperitoneally four times a week from Day 8. Each group comprises mice with n=3. **a**. Representative images of lungs by masson’s trichrome staining, from mice treated with increasing concentrations of Nutlin 3b. Bar indicates 250 μm **b.** Quantification of the fibrotic area in lungs after Nutlin 3b treatment. **c.** Immunoprecipitation to detect PGAM1-Chk1 interactions in fibrosis-induced lungs with or without Nutlin 3b treatment. **d.** Western blotting of FoxM1 in fibrosis-induced lungs from vehicle- or Nutlin 3b-treated mice and from control (each n=3) (left panel). The same samples were applied for immunoprecipitation assay to detect PGAM1-Chk1 interactions (right panels). Data represent the mean ± SEM. Single (*) and double (**) asterisks indicate statistical significance of p<0.05 and p<0.01, respectively. Statistical analyses were performed using one-way analysis of variance (ANOVA) and Dunnett’s multiple comparison test.

**
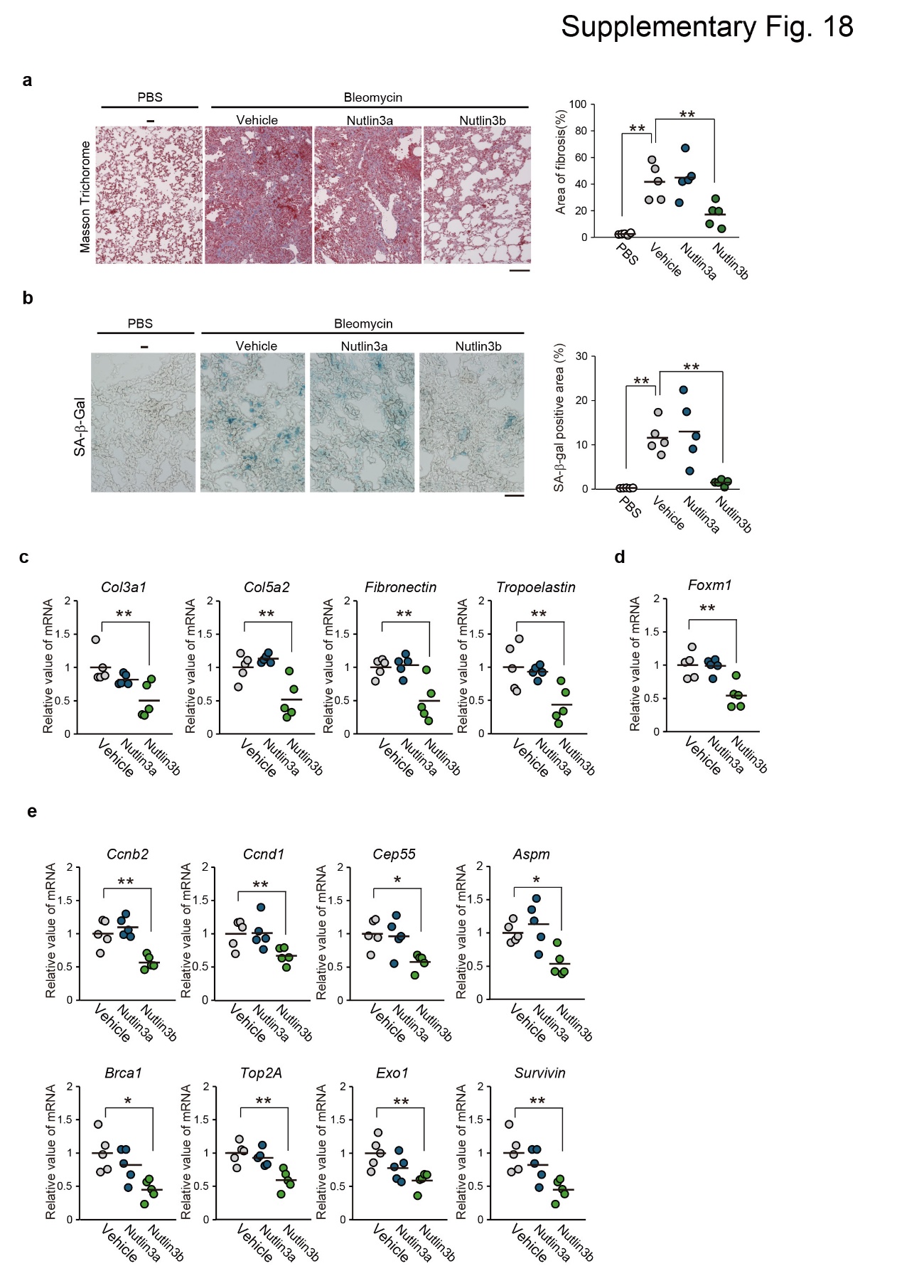
**

**Supplementary Figure 18. Comparison of the therapeutic effects of Nutlin 3a and 3b in a beomycin-induced pulmonary fibrosis model (Relevant to Figure 7).**

Comparison of Nutlin 3a and 3b against lung fibrosis model. Each group comprises mice with n=5. **a**. Representative images of lungs by masson’s trichrome staining, from mice treated with Nutlin 3a or 3b (left panels). Bar indicates 100 μm. Right panel indicates the quantification of the fibrotic area in lungs after Nutlin 3a or 3b treatment. **b.** SA-β-Gal stainings of lungs under Nutlin 3a or 3b treatment in lung fibrosis model. Representative pictures (left panels) and positivity of SA-β-Gal staining (right). Bar indicates 100 μm. **c.** The comparison of mRNAs for fibrotic parameters by RT-PCR between Nutlin 3a and 3b treated group. (**d.**-**e.**) Comparison of mRNAs for FoxM1 (**d.**) and its downstream targets (**e.**) in lungs under Nutlin 3a or 3b in lung fibrosis model. Data represent the mean ± SEM. Single (*) and double (**) asterisks indicate statistical significance of p<0.05 and p<0.01, respectively. Statistical analyses were performed using one-way analysis of variance (ANOVA) and Dunnett’s multiple comparison test.

**
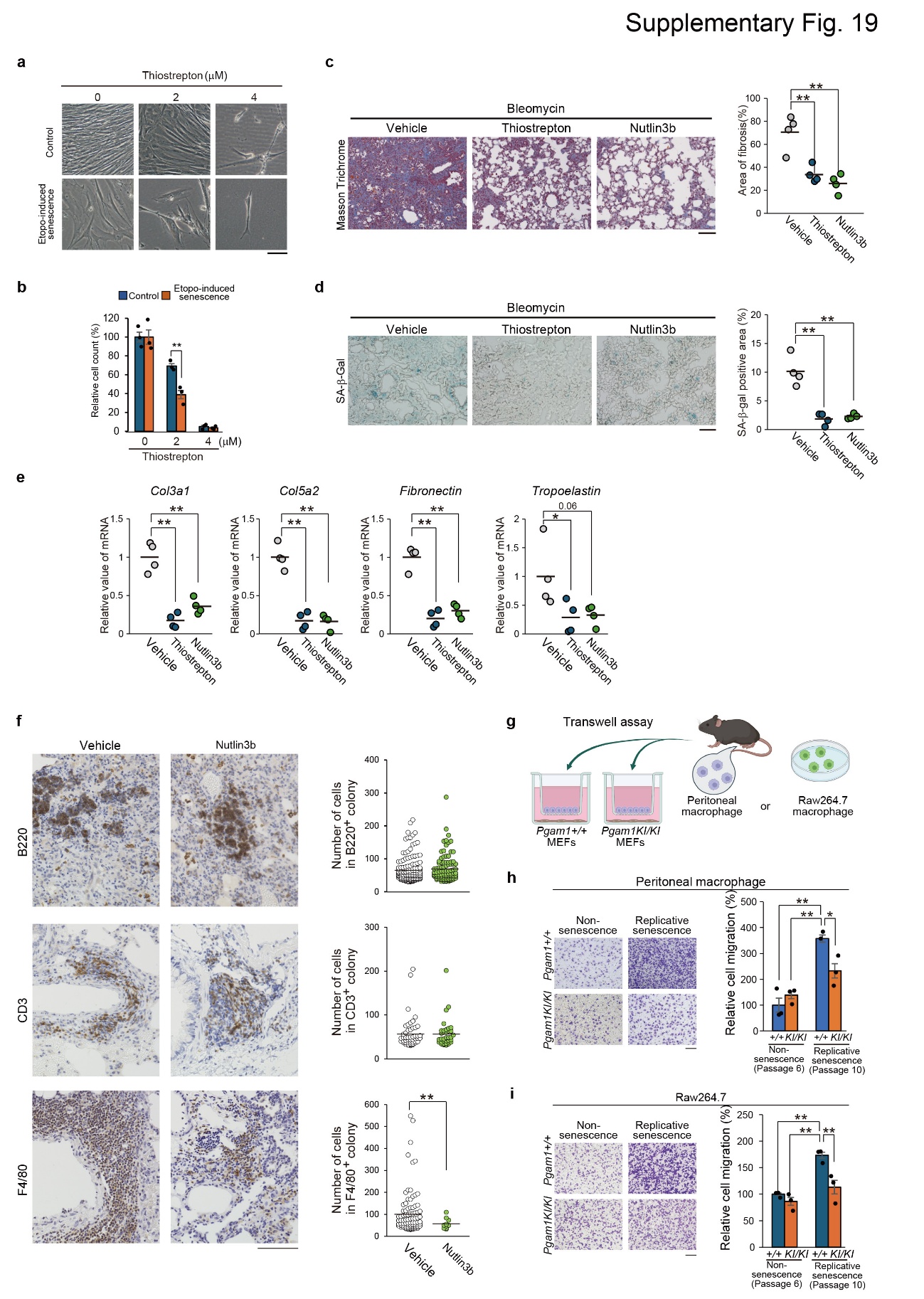
**

**Supplementary Figure 19. Verification of the therapeutic effects of Nutlin 3b in a beomycin-induced pulmonary fibrosis model (Relevant to Figure 7).**

(**a.**-**e.**) The effect of FOXM1 inhibitor, thiostrepton, *in vitro* (**a. -b.**) and *in vivo* (**c.**-**e.**). (**a. -b.**) The *in vitro* effect of inhibitor for FoxM1 on SnCs. SnCs and non-senescent cells were prepared. Representative pictures (**a.**) and cell counts of indicated cells (**b.**) after treatment by thiostrepton. (**c.**-**e.**) Mice with bleomycin-induce lung fibrosis were treated with thiostrepton or Nutlin 3b. Each group comprises mice with n=4. **c.** Representative images of lungs by masson’s trichrome staining, from mice treated with thiostrepton or Nutlin 3b (left panels). Bar indicates 100 μm. Right panel indicates the quantification of the fibrotic area in lungs after thiostrepton or Nutlin 3b treatment. **d.** SA-β-Gal staining of lung after drug treatment. Bar indicates 100 μm. **e.** Evaluation of mRNAs for fibrotic parameters by RT-PCR. **f.** Immunohistochemical analysis of immune cells, including B cell, T cell and macrophage, in lung fibrosis model after Nutlin 3b treatment. B220 antibody for B cells, CD3 antibody for T cells, and F4/80 antibody for macrophages, are utilized, respectively. Representative pictures (left panels) and cell numbers of indicated colonies (right). (**g.**-**i.**) Co-culture assay between macrophages and senescent fibroblasts. **g.** Diagram of co-culture assay. Primary MEFs from WT or *Pgam1 ^KI/KI^* mice were isolated. Replicative senescent MEFs were prepared. Young or replicative senescent MEFs were co-cultured with primary peritoneal macrophages or with Raw264.7 macrophages. (**h.**-**i.**) Young or replicative senescent MEFs were co-cultured with primary peritoneal macrophages (**h.**) or with Raw264.7 macrophages (**i.**). Representative pictures (left panels) and cell migration counts of macrophages (right). Data represent the mean ± SEM. Single (*) and double (**) asterisks indicate statistical significance of p<0.05 and p<0.01, respectively. Statistical analyses were performed using one-way analysis of variance (ANOVA) and Dunnett’s multiple comparison test.

**
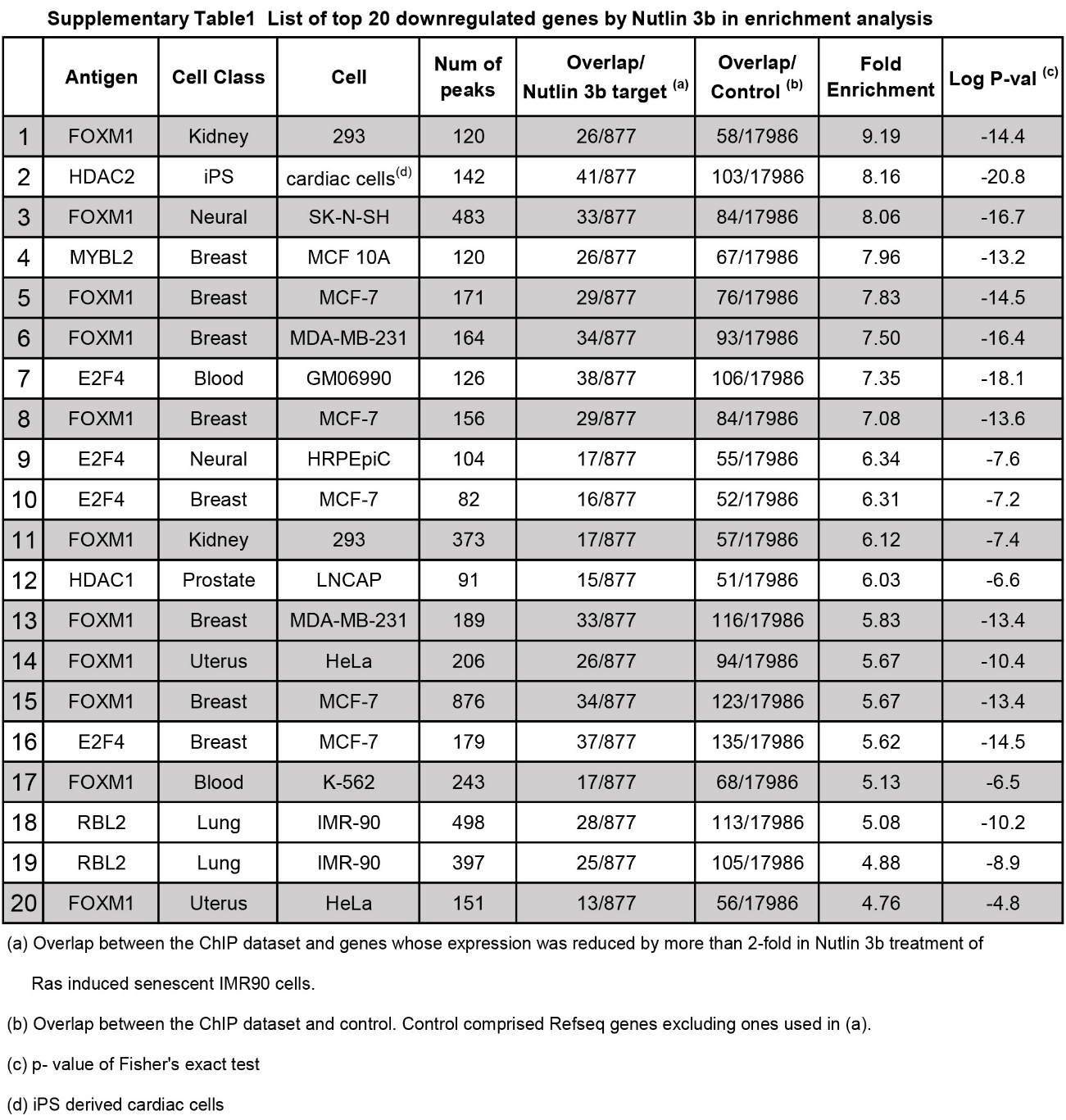
**

**Supplementary Table 1. Enrichment analysis by comparison between 9,442 ChIP datasets and 1,168 genes downregulated by Nutlin 3b demonstrated by microarray analysis.**

25,112 ChIP (chromatin immunoprecipitation) datasets in National Center for Biotechnology Information (NCBI), European Bioinformatics Institute (EBI) and DNA Data Bank of Japan (DDBJ) comprise 903 antigens (transcriptional factors and epigenetic regulators). Among these datasets, 9,442 ChIP datasets demonstrated >10 overlapping genes with information on 1,168 downregulated genes (>2-fold) by Nutlin 3b in senescent cells. Enrichment analysis was performed, based on the comparison between 9,442 ChIP datasets and microarray information (Fig. S12c). Top 20 transcriptional factors were listed, according to high hit scores by fold enrichment analysis of comparative transcriptomic data.

**
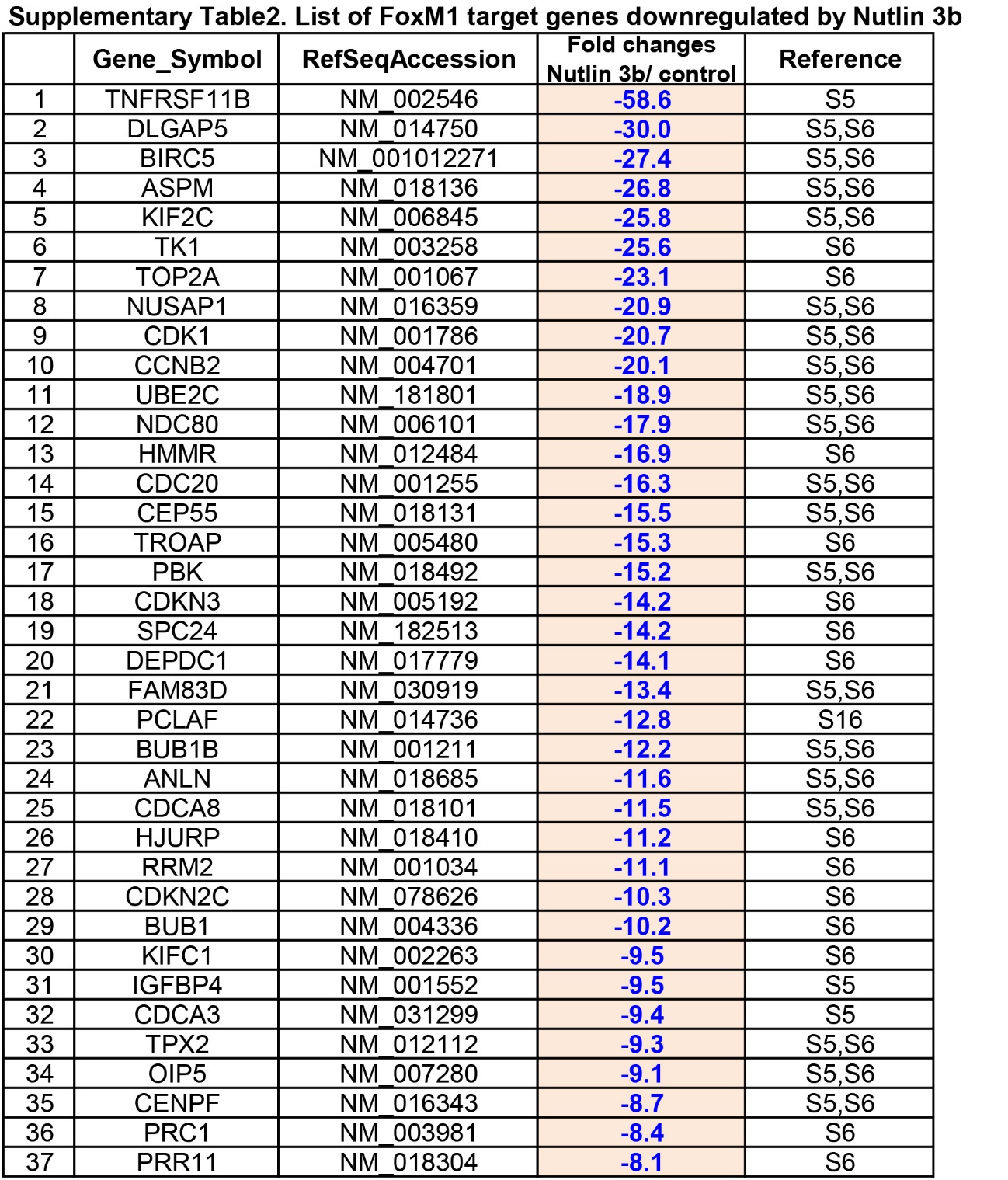
**

**Supplementary** **Table 2. List of FOXM1 target genes downregulated by Nutlin 3b.**

Among the 150 most downregulated genes (<-8.1-fold) by Nutlin 3b in SnCs, FOXM1 target genes were listed based on indicated references.

**
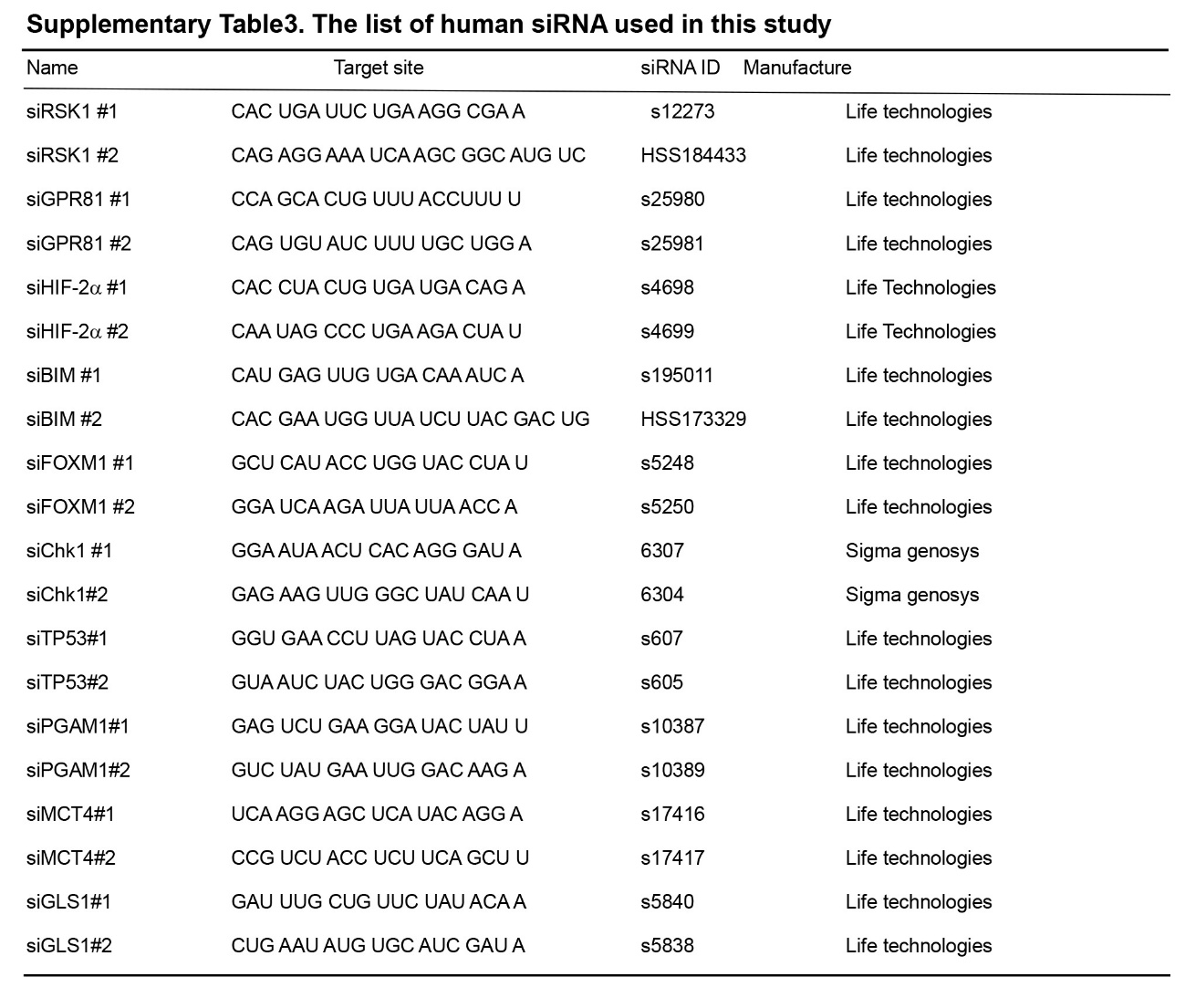
**

**Supplementary Table 3. List of human siRNAs used in this study.**

Target site, siRNA IDs, and manufacturers for each target gene are described.


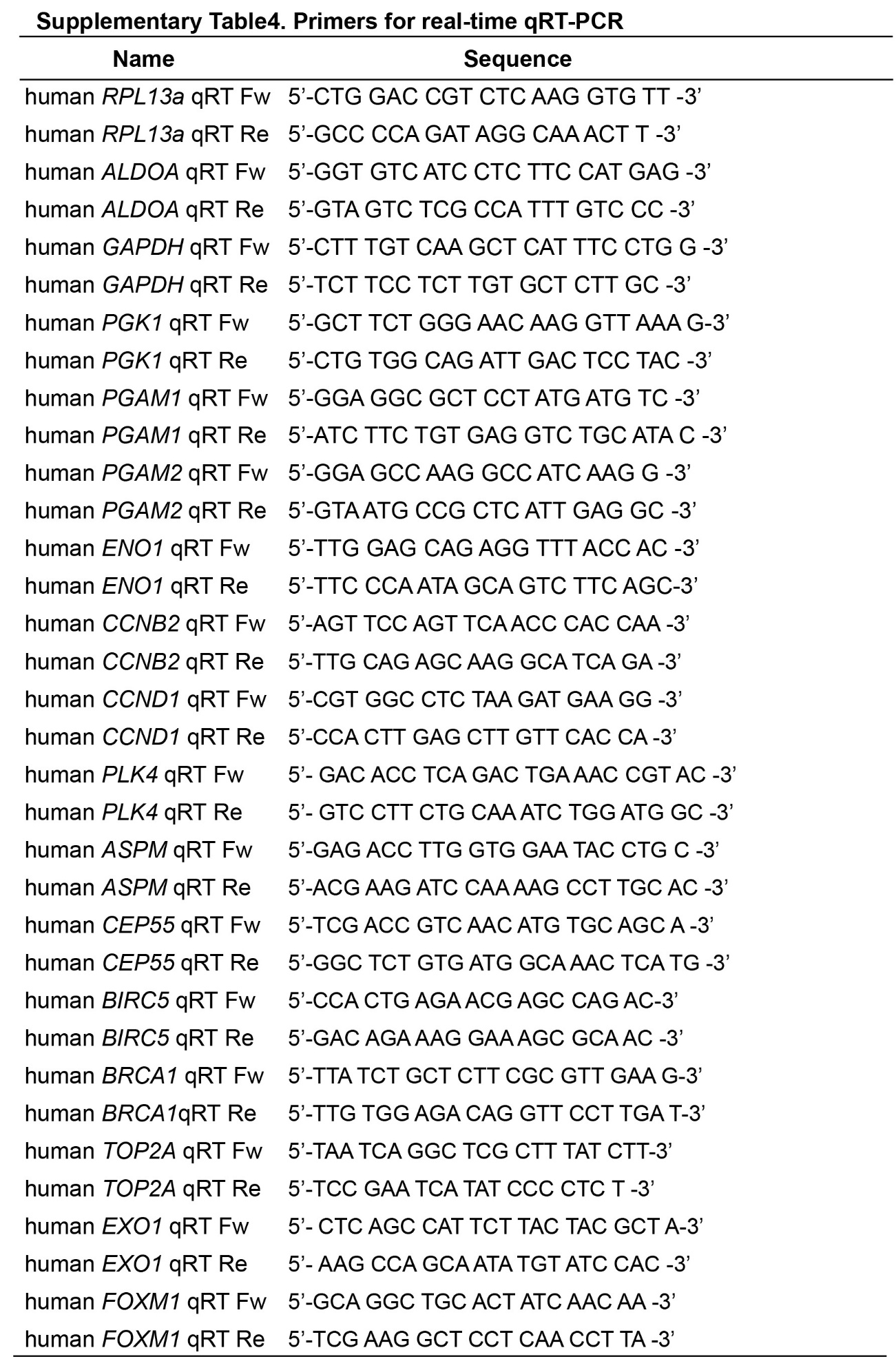


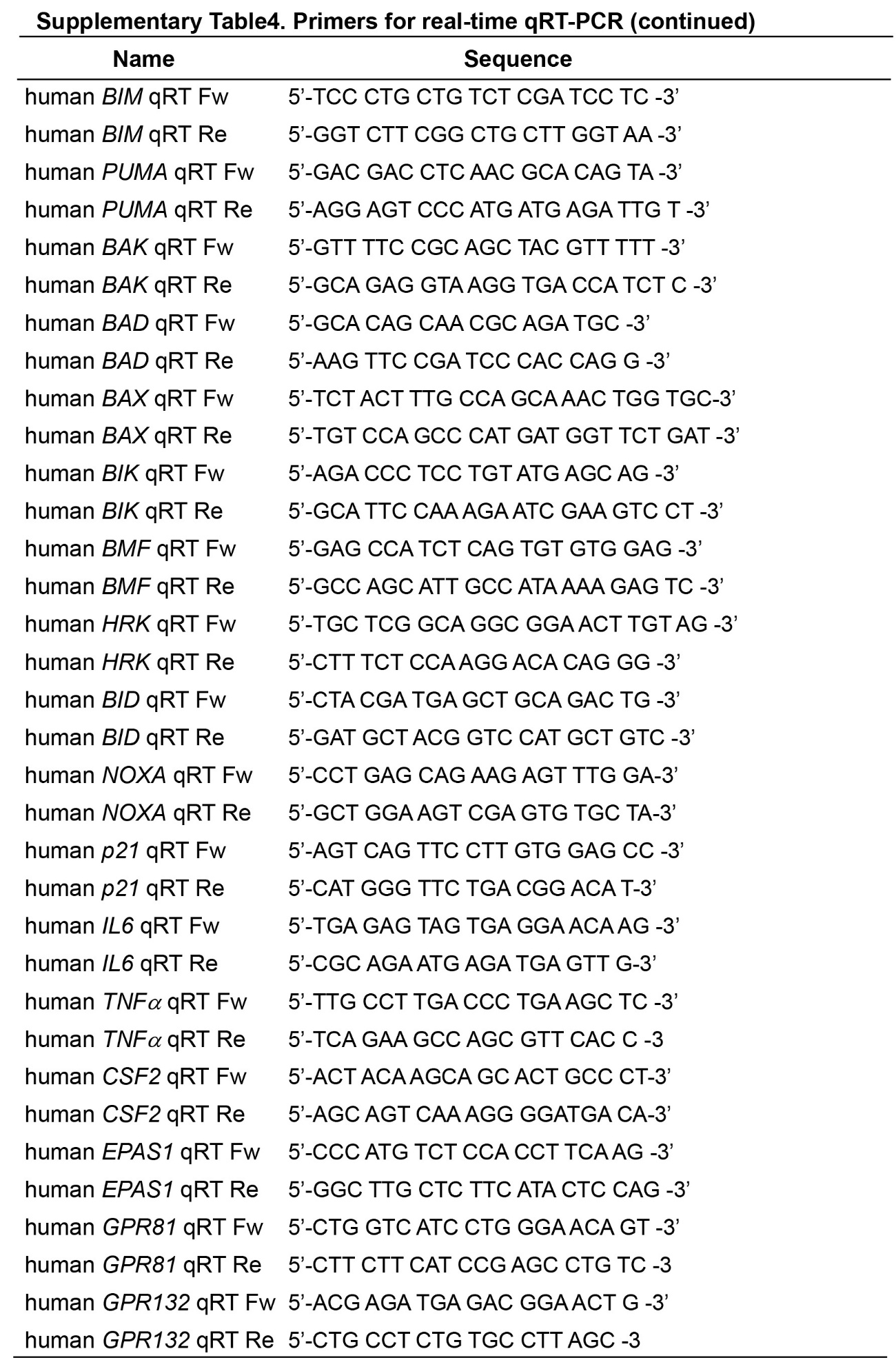


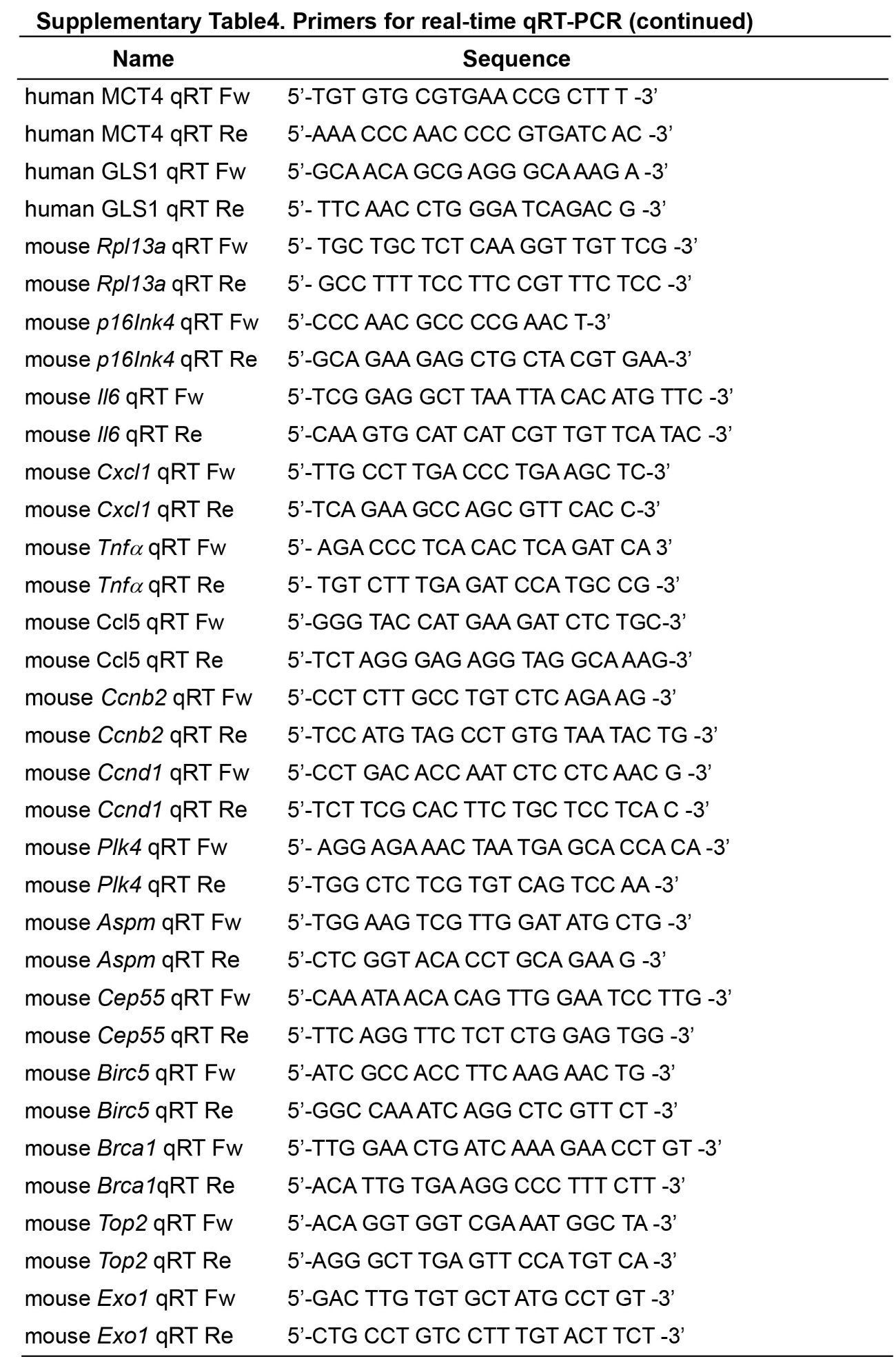


**
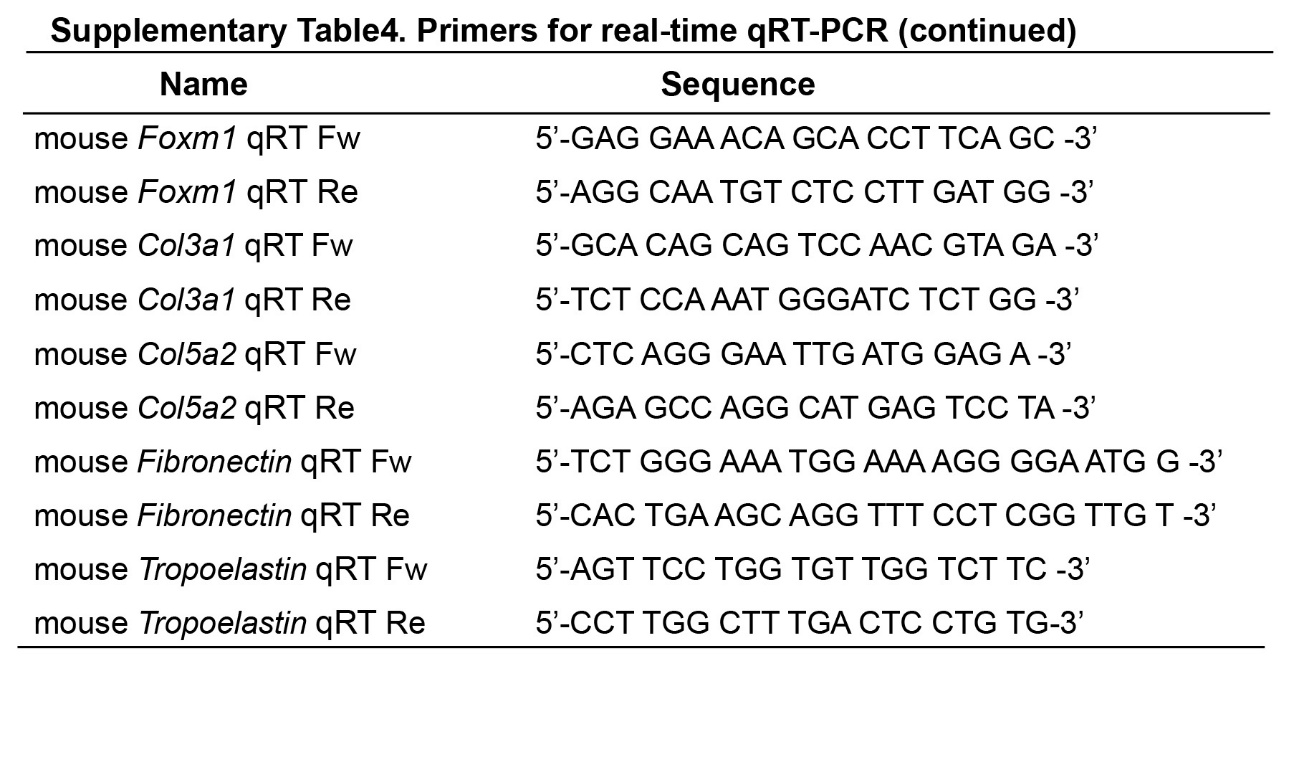
**

**Supplementary Table 4. List of primers for RT-PCR.**

Relevant sequences for each target gene are described.


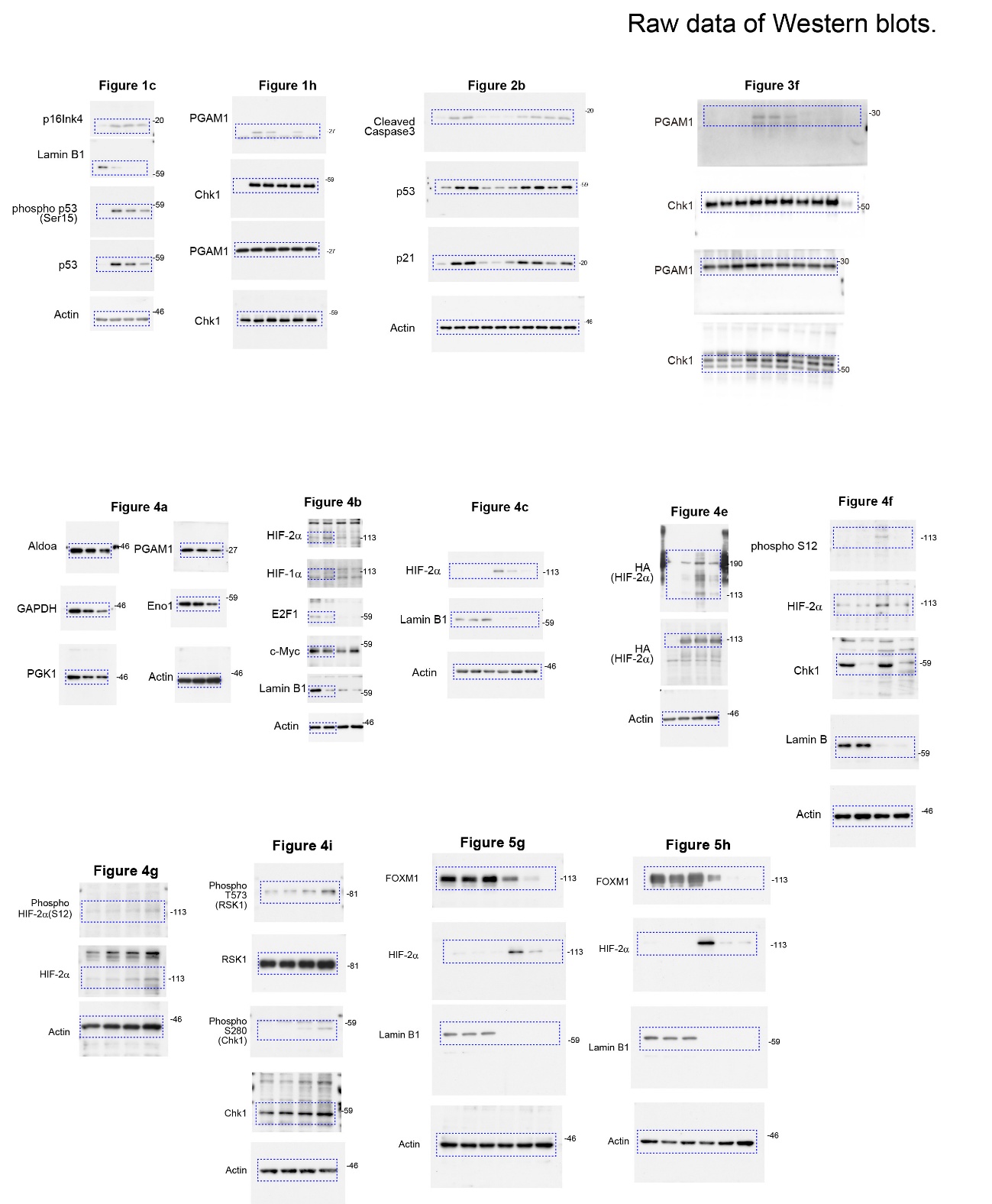


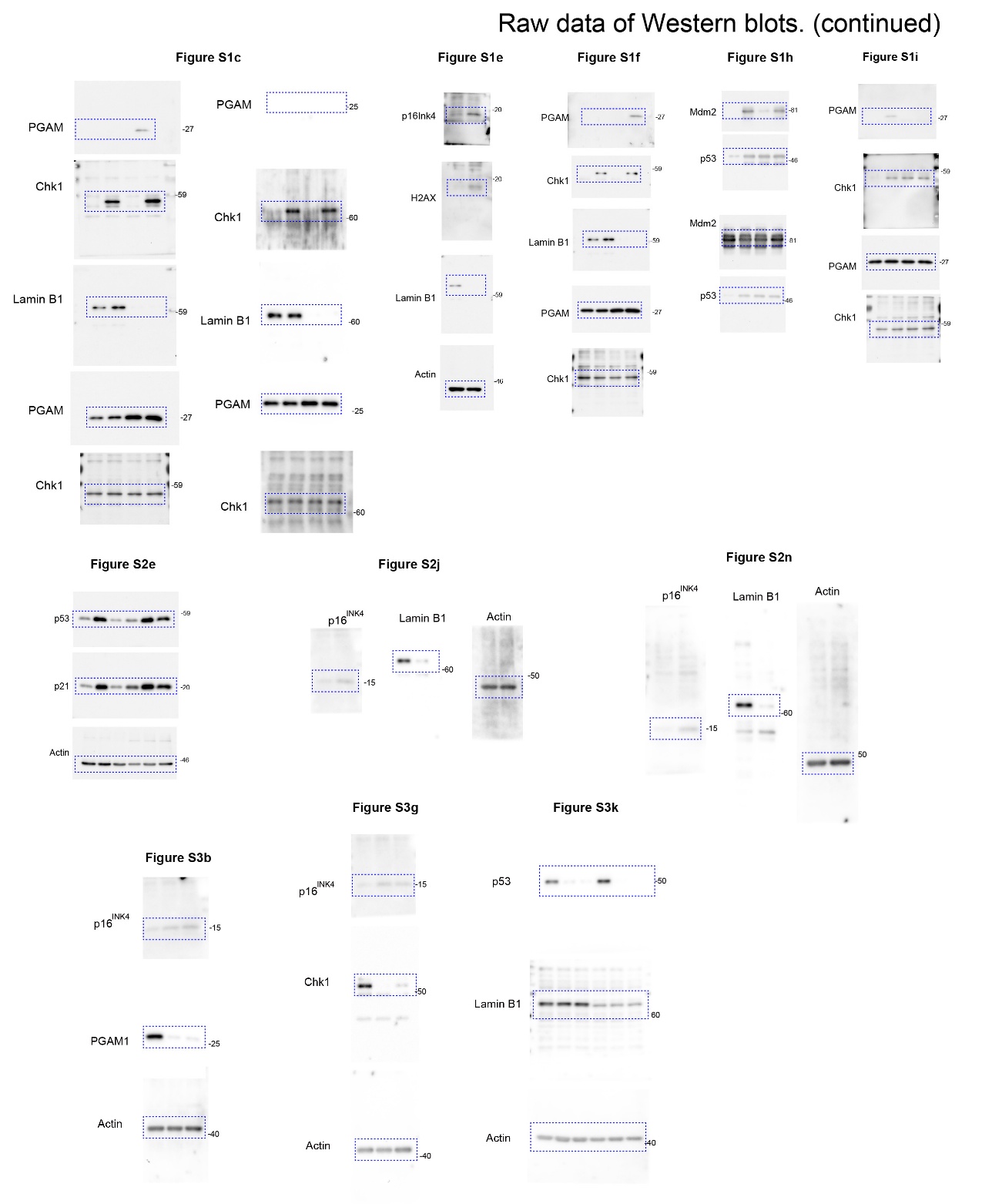


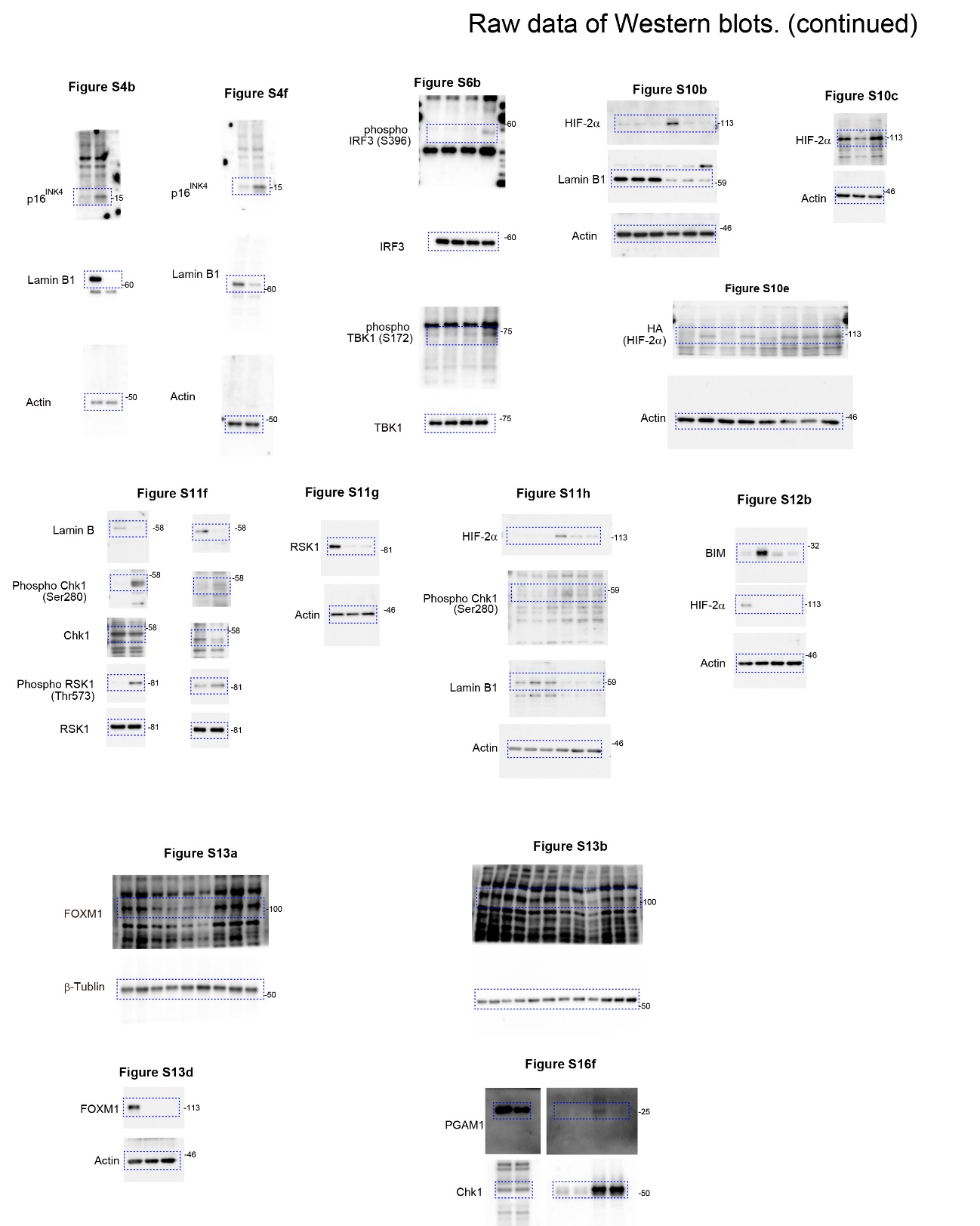


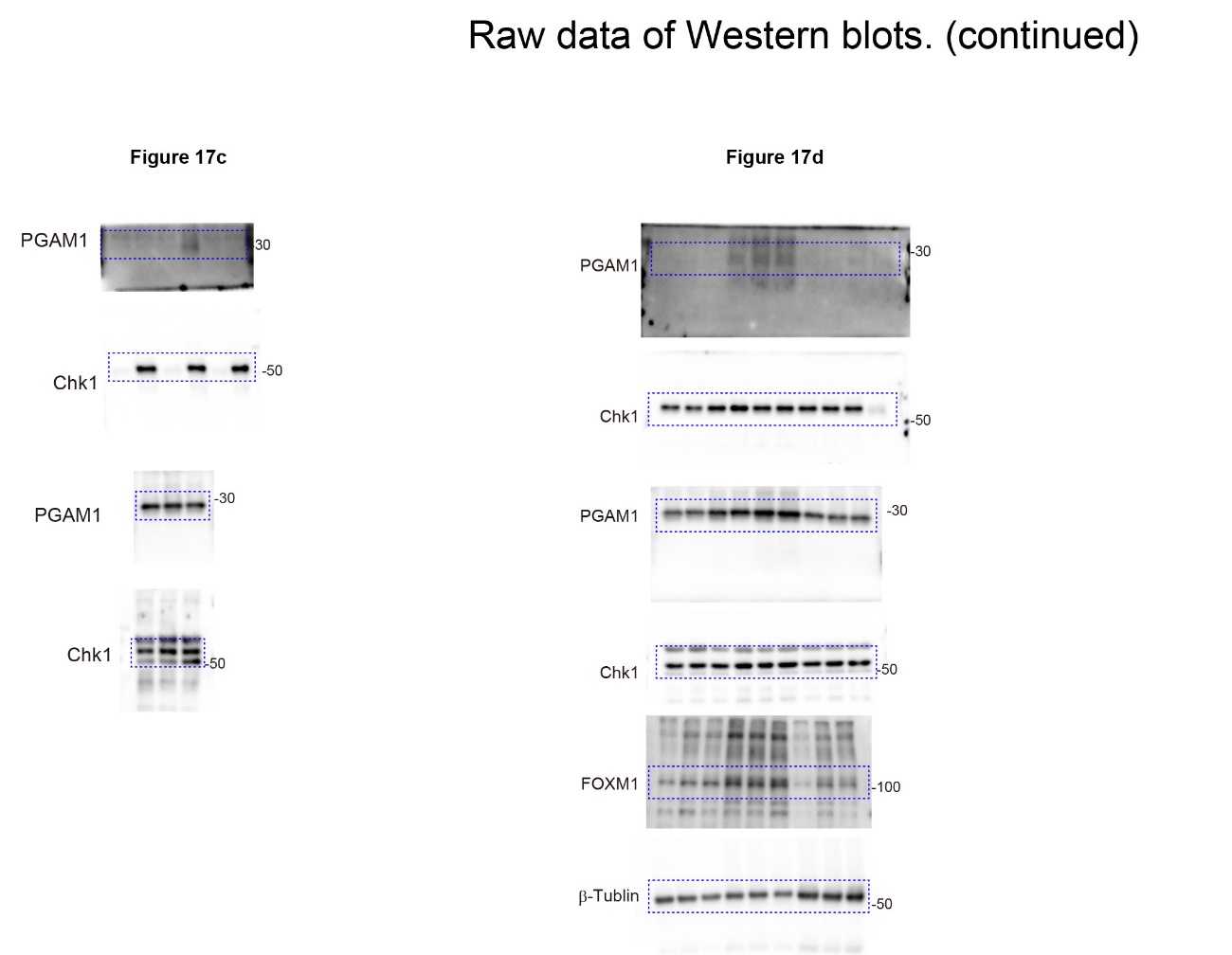


**Supplemental References**

S1 Young, A. R. *et al.* Autophagy mediates the mitotic senescence transition. *Genes Dev* **23**, 798-803 (2009).

S2 Yan, Q. *et al*. The hypoxia-inducible factor 2alpha N-terminal and C-terminal transactivation domains cooperate to promote renal tumorigenesis in vivo. *Mol Cell Biol* **27**, 2092-102 (2007).

S3 Bai, C., Liu, X., Qiu, C. & Zheng, J. FoxM1 is regulated by both HIF-1alpha and HIF-2alpha and contributes to gastrointestinal stromal tumor progression. Gastric Cancer 22, 91-103 (2019).

S 4 Morioka, S. et al. A mass spectrometric method for in-depth profiling of phosphoinositide regioisomers and their disease-associated regulation. Nat Commun 13, 83 (2022).

S5 Macedo, J. C. *et al.* FoxM1 repression during human aging leads to mitotic decline and aneuploidy-driven full senescence. *Nat Commun* **9**, 2834 (2018).

S6 ChIP Atlas https://chip-atlas.org
